# BIOCOM-PIPE: a new user-friendly metabarcoding pipeline for the characterization of microbial diversity from 16S, 18S and 23S rRNA gene amplicons

**DOI:** 10.1186/s12859-020-03829-3

**Published:** 2020-10-31

**Authors:** Christophe Djemiel, Samuel Dequiedt, Battle Karimi, Aurélien Cottin, Thibault Girier, Yassin El Djoudi, Patrick Wincker, Mélanie Lelièvre, Samuel Mondy, Nicolas Chemidlin Prévost-Bouré, Pierre-Alain Maron, Lionel Ranjard, Sébastien Terrat

**Affiliations:** 1 Agroécologie, AgroSup Dijon, INRAE, Univ. Bourgogne, Univ. Bourgogne Franche-Comté, 21000 Dijon, France; 2 CEA/Institut de Biologie François Jacob/Génoscope, 2, Rue Gaston Crémieux, CP5706, 91057 Evry Cedex, France; 3 Agroécologie - Plateforme GenoSol, BP 86510, 21000 Dijon, France

**Keywords:** Ecology, Metabarcoding, Bacterial, Archaeal, Fungal, Photosynthetic microeukaryotes, ReClustOR, France, Land-use

## Abstract

**Background:**

The ability to compare samples or studies easily using metabarcoding so as to better interpret microbial ecology results is an upcoming challenge. A growing number of metabarcoding pipelines are available, each with its own benefits and limitations. However, very few have been developed to offer the opportunity to characterize various microbial communities (e.g., archaea, bacteria, fungi, photosynthetic microeukaryotes) with the same tool.

**Results:**

BIOCOM-PIPE is a flexible and independent suite of tools for processing data from high-throughput sequencing technologies, Roche 454 and Illumina platforms, and focused on the diversity of archaeal, bacterial, fungal, and photosynthetic microeukaryote amplicons. Various original methods were implemented in BIOCOM-PIPE to (1) remove chimeras based on read abundance, (2) align sequences with structure-based alignments of RNA homologs using covariance models, and (3) a post-clustering tool (ReClustOR) to improve OTUs consistency based on a reference OTU database. The comparison with two other pipelines (FROGS and mothur) and Amplicon Sequence Variant definition highlighted that BIOCOM-PIPE was better at discriminating land use groups.

**Conclusions:**

The BIOCOM-PIPE pipeline makes it possible to analyze 16S, 18S and 23S rRNA genes in the same packaged tool. The new post-clustering approach defines a biological database from previously analyzed samples and performs post-clustering of reads with this reference database by using open-reference clustering. This makes it easier to compare projects from various sequencing runs, and increased the congruence among results. For all users, the pipeline was developed to allow for adding or modifying the components, the databases and the bioinformatics tools easily, giving high modularity for each analysis.

## Background

Interest in microbial ecology has been revived in the last 2 decades largely thanks to meta-omics approaches and more specifically to metabarcoding based on high-throughput DNA sequencing. This has substantially improved knowledge on the interactions of microorganisms with their environment and also with one another. This approach has become a reference for characterizing biodiversity at a reasonable cost [1].

The “Big Data” phenomenon has emerged as a powerful informative source in microbial ecology, but this field requires technical and financial support to be explored. Among others, the datasets generated by second- and now third-generation DNA sequencing require the ongoing development of algorithms, software, pipelines and standard operating procedures for data analysis, interpretation and reproducibility [1, 2]. Many popular (e.g., mothur, QIIME, FROGS) or new (sl1P, iMAP, ANCHOR) pipelines are currently dedicated to the analysis of ribosomal DNA sequence data and are becoming more and more accessible to scientists from various fields [3–8]. The choice to use one tool among others remains very variable and often constrained by different parameters (e.g., computer infrastructure, available staff, clustering method, database availability). In fact, each pipeline provides a full set of tools often associated with its own chimera detection and/or OTU clustering methods, and with only one or a few reference taxonomy databases available. One of the key steps is the detection of chimeric sequences, and various methods exist that may impact on the downstream analysis [9]. The story is the same for clustering sequences into OTUs, another key step: microbial structure or diversity can be impacted depending on the chosen approach [10, 11]. However, one of the upcoming challenges is to make it easier to compare datasets from different projects. Otherwise it is not reliable to compare the results of a given study with those of other studies [12]. We propose a new user-friendly pipeline called BIOCOM-PIPE (initially referred to GnS-PIPE in previous studies [13]), developed to be used by biologists as well as computer scientists. More precisely, BIOCOM-PIPE also relies on the expertise of the GenoSol platform^1^ to conduct biological validations for defined bioinformatic steps. BIOCOM-PIPE performs diversity analysis of prokaryotic (archaea, bacteria) and eukaryotic (fungi, photosynthetic microeukaryotes) organisms and is particularly effective for large datasets such as the RMQS, Tara Oceans and Earth or Human Microbiome projects [14–16]. BIOCOM-PIPE is completely modular, as the user can choose one or several available modules, tools, or programs, with default or expert parameters. Moreover, this pipeline integrates ReClustOR, an innovative approach aimed at defining a biological reference database from a set of analyzed samples, relying on a post-clustering tool that improves the reliability of OTU-based results and analyses [17]. Lastly, a comparison of different pipelines dedicated to analyze amplicon sequence data (mothur and FROGS) and ASV definition was performed using several datasets (a simulated, an artificial and a real dataset) to highlight differences in terms of chimera detection, clustering method and consistency, and taxonomic assignment.

## Methods

### Workflow: a brief description of the BIOCOM-PIPE pipeline

**Fig. 1:**
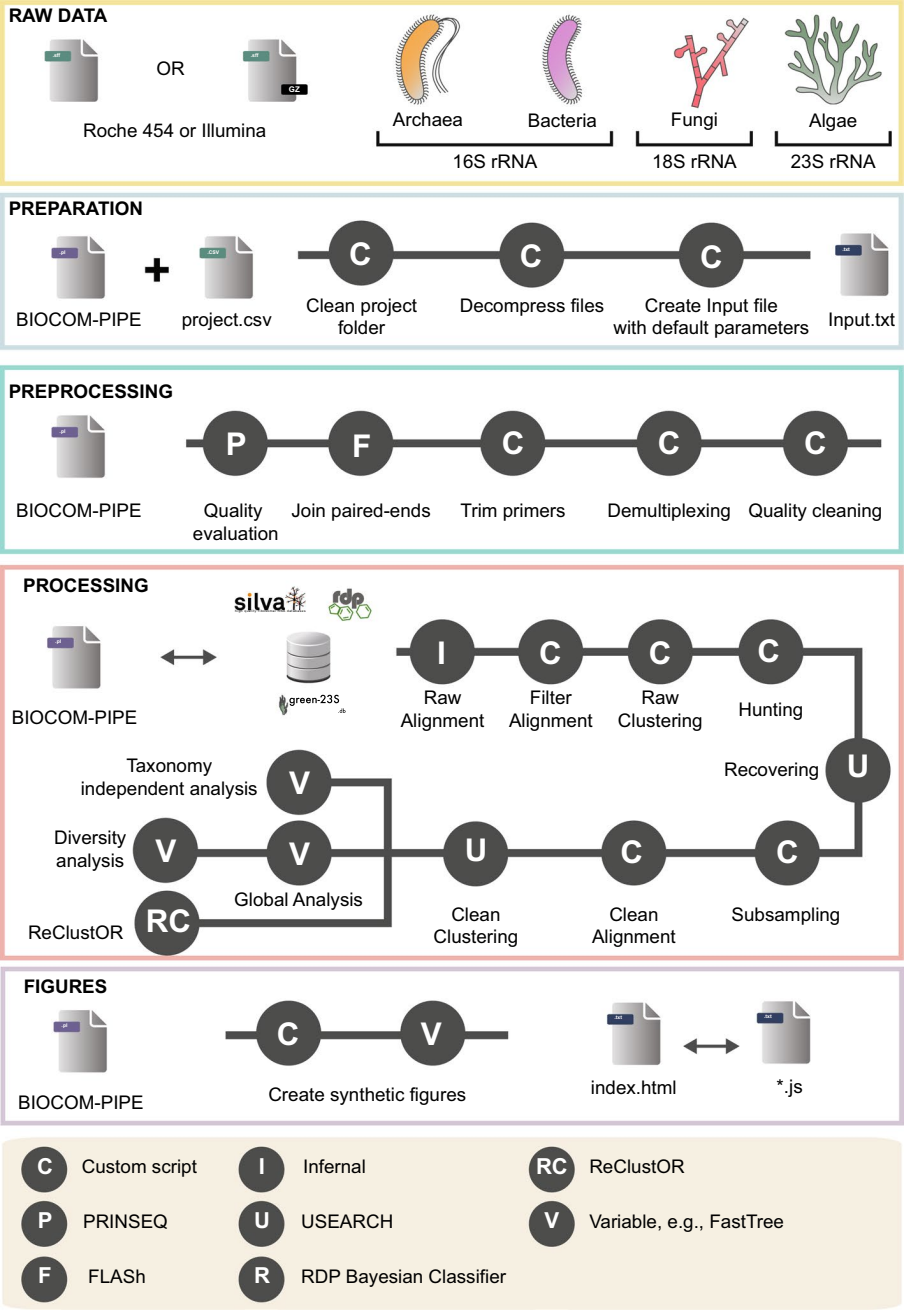
Schematic illustration of the BIOCOM-PIPE pipeline. The initial step in the analysis consists in checking uniqueness and preparing the files required to run the pipeline (light blue box). It is followed by a pre-processing step to check the quality of reads. Read pairs are demultiplexed if necessary, merged, and primers are trimmed (green box). The next key step consists in processing the clean reads by aligning them with a reference structure, hunting-recovering chimeras, clustering and post-clustering, and ending with diversity analysis (red box). The last step consists in generating a complete website providing all the results to be presented in tabular and graphics forms (purple box)

BIOCOM-PIPE is a bioinformatic pipeline defined to characterize various amplicon sequence datasets from 16S, 18S and 23S marker genes. It includes steps dedicated to clean the raw data files, pre-process the raw reads to reduce sequencing and PCR errors, and process the cleaned reads to define OTUs and classify all sequences with a taxonomic assignment (Fig. 1). Many tools and methods were included, such as PRINSEQ [18], FLASH [19], Usearch [20], FastTree, Infernal [21], RDP Bayesian Classifier [22], UniFrac [23] and ReClustOR [17]. The main results are available through a local website with arrays and dynamic graphics for better scalability and usability (Additional file 1: Doc S1). The complete pipeline with associated taxonomic databases can be downloaded from the Zenodo repository (10.5281/zenodo.3678129).

### Implementation

BIOCOM-PIPE was mainly developed in PERL (v5.16.0), except some specific components of the workflow written in Python (v2.7) or in C language and encompassing various tools (Additional file 2: Figure S1). These third-party programs must be installed (and potentially compiled for some of them) in specific folders. Dedicated links to these tools with validated versions and specific installation procedures are also directly available from the Zenodo repository (10.5281/zenodo.3678129). A virtual machine encompassing the complete pipeline into an UBUNTU system is also available (10.5281/zenodo.3945964).

A dataset example is made available to understand BIOCOM-PIPE and check the installation process (Additional file 1: Doc S1, see Tutorial section). Moreover, all input and expected output files for this example are also available (https://doi.org/10.5281/zenodo.3947784). All steps are optimized and/or parallelized, and automatically integrated in BIOCOM-PIPE. It is noteworthy that amplicon reads from both Roche 454 or Illumina sequencing technologies can be treated (Fasta reads from other sources too). Moreover, this pipeline is completely modular, so that amplicon reads can be in paired ends or not, with or without multiplex identifiers (managing single or dual index) and/or primers, and in dereplicated reads or not. The integrated taxonomic databases were filtered based on standards detailed in supplementary materials (Additional file 1: Doc S1).

### Bioinformatics analysis: robustness and reproducibility

For better flexibility of the setting-up analysis, this pipeline was designed to be used with command lines in UNIX environments, but it can also run on POSIX-compatible platforms, such as MacOS/X. However, to facilitate its use for scientists unfamiliar with command lines, it can be used by completing a project file (project.csv, (Additional file 1: Doc S1)) and by running the pipeline. The information in the project file is then retrieved to launch the pipeline with default parameters. For traceability reasons of current projects but also for manageability, this project file is mandatory to have the configuration file generated (Input.txt) (Additional file 1: Doc S1). The project file is a simple tabulated file located in the Project_file subfolder (which will also contain the generated summary files and the local website) (Additional file 3: Figure S2).

It is also mandatory to define the steps to be performed (an example is available in Additional file 1: Doc S1). This Input.txt file lists all the available steps, with associated parameters, and can be handled easily, even for users not fluent in bioinformatic. For example, to manage the workflow definition easily, all steps have one common parameter represented by one question, “Step to do [yes–no]?” (Additional file 1: Doc S1). Each parameter can be defined using an answer to a question asked in this configuration file. For each step, the user has access to one or several text file reports integrating both user choices and results, also provided by the local website. Consequently, when the analysis is complete, the user can choose between two alternatives, either by retrieving the summary files from the folder tree "Summary_files" or by using the local website generated in the “website” folder (Additional file 3: Figure S2). This way the user can effectively access to results and parameters with tables or interactive charts (Additional file 1: Doc S1).

### Step 1: preparation files

As detailed above, the preparation files represent a first step. Although this step is not systematically required, it is strongly recommended to run it to: (1) clean the project directory, if previous analyses have been performed but not deleted, (2) extract the raw data if datasets were compressed, and (3) create the default configuration file depending on the molecular marker (16S, 18S or 23S) and the used sequencing technology (Roche or Illumina).

### Step 2: data pre-processing

Pre-processing includes several components that can be combined or launched independently (Fig. 1). Firstly, the initial trimming of raw reads can be performed using PRINSEQ [18]. It permits to efficiently check and prepare datasets prior downstream analyses, and obviously improve the merging of paired-end reads using FLASH [19]. Following this merging (if necessary), the demultiplexing tool can be applied, and all reads without an existing multiplex identifier will be deleted for further steps. Then, all selected raw reads can be preprocessed, and low-quality reads can be deleted based on their minimum length, their number of ambiguities (Ns), and their primer sequences depending on user choices selected in the configuration file. The last step of data pre-processing is automatically applied to save computing time and consists in rigorous dereplication (i.e., clustering of strictly identical sequences).

### Step 3: “hunting–recovering” concept: chimera detection and filtering process

The “hunting–recovering” process encompasses four different steps (global alignment, clustering, hunting, and recovering). It is devoted to efficiently identify and delete reads with high-error rates as well as potential chimeric reads (Fig. 1). This step relies on the clustering of reads to define OTUs and then check for chimeras in low-abundance OTUs.

The clustering step of BIOCOM-PIPE relies on global alignment of all reads with Infernal [21], a program developed to create multiple sequence- and structure-based alignments of RNA homologs using covariance models. Then, all reads are clustered into OTUs using a homemade de novo clustering approach similar to CrunchClust [17, 24]. Briefly, this clustering is based on a greedy strategy in which OTUs are constructed incrementally by comparing an abundance-ordered list of input reads against a representative set of already chosen sequences. An OTU is defined by the most abundant read, known as the centroid, and each read in the OTU must display similarity (computed using Levenshtein distance) above the pre-defined identity threshold with the centroid. Moreover, global alignment provides a more intuitive handling of sequencing errors, such as homopolymer errors (from 454 pyrosequencing reads for example) that are easily detected thanks to the secondary-structure-aware aligner. Therefore, such homopolymer errors can be easily ignored during the clustering step if needed. A step is dedicated to the filtering of sequences based on the quality of alignment against the chosen rRNA structure.

In order to identify the chimeras produced during PCR amplification [25], various tools based on reference or de novo approaches (e.g., ChimeraSlayer [26], Perseus [27], UCHIME [28] or VSEARCH [29]) have already been developed.

In BIOCOM-PIPE, an alternative approach was implemented, named “Hunting–Recovering”. The “Hunting” step removes the rarest OTUs according to a defined threshold. For example, if an OTU is represented by 0.01% of reads or less (1 read out of 10,000 reads in total), it will be deleted only if it is unique (defined as a single singleton). It is noteworthy that all OTUs with one read will always be deleted during this step. Then, the “Recovering” step can be applied to check the quality of all discarded reads after the “Hunting” step, based on their taxonomic assignments. More precisely, all discarded reads are compared with a dedicated reference database (e.g. SILVA, RDP) [30, 31] using similarity approaches with USEARCH [20], and kept only if their identity is higher than the defined threshold at one specific taxonomic level. The user can choose to (1) keep all non-clustered singleton reads (no hunting-recovering step), or (2) delete them (only the hunting step is computed), close to some filters advised [32], or (3) carefully check singleton reads using the “Hunting–Recovering” process (both steps are selected). The stringency of this chimera detection can be completely optimized by each user, depending on used steps, chosen taxonomic databases and/or defined parameters.

Lastly, a rarefying step can be applied to standardize the numbers of high-quality reads per sample, based on user choice. This step is recommended before subsequent analyses, to compare the community composition (alpha-diversity) of various samples efficiently [33].

### Taxonomic analysis

The BIOCOM-PIPE taxonomic affiliation strategy is based on the complete classification of all high-quality reads, not just one representative read for each OTU. This step is performed using either the RDP classifier tool [22] or USEARCH [20] against SILVA (16S/18S) [30], Greengenes (16S) [34] or µgreen (23S) [35] (https://zenodo.org/record/3387214#.Xk5eABrQjUI) databases (Fig. 1). For the Archaea and Bacteria domains (16S rRNA) and the Fungi kingdom (18S rRNA), two releases of SILVA (r114 and r132) are available. These databases were filtered based on standards detailed in supplementary materials (Additional file 1: Doc S1). For photosynthetic microeukaryotes (23S rRNA), µgreen-db r1.1 is also available (https://zenodo.org/record/3387214#.XZdIHOnVLUJ).

### Step 4: global analysis

Global analysis is an essential step to fully analyze the treated dataset. It consists in performing multiple sequence alignment based on sequence similarities and rRNA secondary structure from previous steps. This makes it possible to compute (1) a global OTU matrix, (2) the taxonomic assignment of OTUs, (3) a de novo phylogenetic tree based on global alignment with all reads or the representative read for each OTU, and (4) UniFrac phylogenetic distances.

### Step 5: OTU-based analysis

For α-diversity analyses based on OTUs, a summary file can be generated with the most common metrics (e.g., Chao1, ACE, Bootstrap, Shannon, Simpson, Evenness). We also provide rarefaction (describing the number of OTUs observed as a function of the sampling effort) and rank abundance (describing the number of sequences distributed across the OTUs) curves.

For β-diversity measurements, one pairwise distance matrix (Bray–Curtis) is computed from the global analysis. It is also possible to use phylogenetic metrics (UniFrac) [23, 36]. To do this, BIOCOM-PIPE integrates the PycoGent package to compute UniFrac pairwise distances and phylogenetic distances (also called PD’s) [37]. For tree-based distance metrics, the phylogeny can be automatically generated with FastTree tool [38] based on global alignment from Infernal [21].

## Benchmarking

### Simulated and artificial datasets

To highlight the robustness and efficiency of BIOCOM-PIPE, two specific datasets were analyzed: a simulated dataset, and a defined microbial mock community. The simulated dataset was defined in silico with synthetic communities from three different environments: the human gut, the ocean and the soil [39]. Representative *genera* were selected after identifying the 80 most abundant genera across publicly available metagenomes from human gut, ocean, and soil. This dataset represents various regions (V1–V2, V3–V4, etc.) of the 16S rRNA gene and is composed of two subsets of sample for each environment with two levels of diversity (100 species, or 500 species) and two levels of sequencing depth (10,000 or 200,000 reads). To be close to our environmental dataset, only the V3-V4 hypervariable regions of the bacterial 16S rRNA gene as if from Illumina platform, representing 24 different samples.

The artificial dataset is a microbial community from ZymoBIOMICS. More precisely, it is a mock community consisting of eight bacterial and two fungal strains. It includes three easy-to-lyse Gram-negative bacteria (*e.g. Escherichia coli*), five tough-to-lyse Gram-positive bacteria (*e.g. Listeria monocytogenes*), and two tough-to-lyse yeasts (*e.g. Cryptococcus neoformans*). The 16S/18S rRNA sequences (FASTA format) and genomes (FASTA format) of these strains are available at: https://s3.amazonaws.com/zymo-files/BioPool/ZymoBIOMICS.STD.refseq.v2.zip. The amplicon library construction is described in [13]. The V3–V4 regions of the 16S rRNA genes generated using Illumina MiSeq technology (4 and 3 replicates from two independent runs) and were deposited into a specific ZENODO repository (https://doi.org/10.5281/zenodo.3947843).

### The illustrative application

To achieve benchmarking and illustrate the different steps of the pipelines, we used also a recently released large dataset of soil samples originating from the RMQS project (French Soil Quality Monitoring Network) collected between 2000 and 2009, which covers the whole French territory [13].

**Fig. 2:**
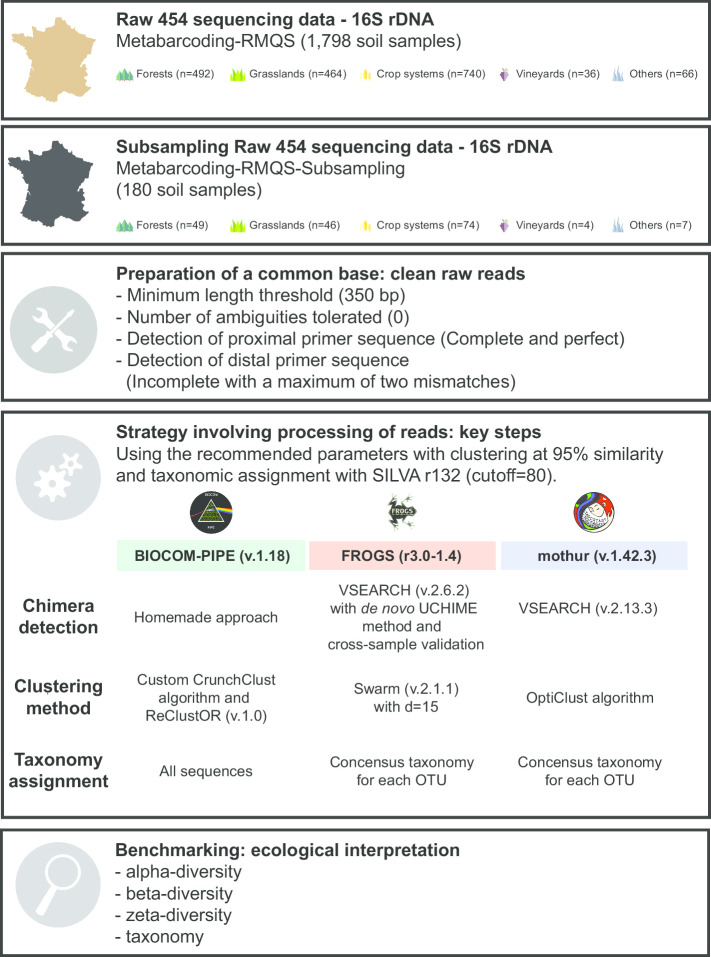
Schematic overview of the different steps of the metabarcoding study, with soil samples from the RMQS project as an example

For practical reasons linked to computing time, and for this work to be reproducible by the large majority of the scientific community, we used a subsampling of 180 soil samples out of a total of 1798. For each of the four main land use types and the “others” group (which contained various minor land use types), we randomly retrieved 10% of its samples to maintain the same representativeness as in the RMQS (crop systems: 74, forests: 49, grasslands: 46, vineyards: 4, and others: 7) (Fig. 2). To check if this subsampling fully reflected the RMQS, soil texture was examined, and the samples were found homogeneously distributed across the soil textural triangle (Additional file 4: Figure S3). The amplicon library construction is described in [13]. The V3–V4 regions of the 16S rRNA genes generated using 454-pyrosequencing technology were downloaded from the EBI database system under project accession No PRJEB21351 (Fig. 2).

### Metabarcoding benchmarking

Two pipelines were selected for benchmarking,* i.e*., mothur (v.1.42.3) and FROGS (r3), with distinct approaches to characterize the soil microbial communities (Fig. 2). Raw reads were cleaned with a common base and then clustered at: 97% and 95% for the simulated dataset, and at 95% only for other datasets for BIOCOM-PIPE based on a homemade clustering tool similar to CrunchClust [17] and on mothur based on opticlust algorithm [40]. Amplicon sequence variants (or ASV) were also used to describe the studied microbial communities. Indeed, the recent, simple and fast method called ASV or exact sequence variant (ESV) defines OTUs from a 100% sequence similarity threshold (zero-radius OTUs or ZOTUs), and provides the highest possible biological resolution. We chose to use the ASV from BIOCOM-PIPE and not from a dedicated tool like DADA2 [41], as the 180 samples selected from the real dataset were encompassed into 80 libraries of 454-sequencing, and DADA2 needs to learn the Error rates of the 80 libraries, a quite time-consuming step. With FROGS clustering based on Swarm tool, a distance fixed to d = 15 was used for the RMQS dataset and the mock community, as close to the 95% identity threshold as possible typically assigned to the same genus [42, 43]. For the simulated dataset, both distances (default and d = 15) were applied. Subsampling and rarefying data are important steps, and we set subsampling to 8000 sequences per sample based on the smallest sample whatever the pipeline for the RMQS dataset. The subsampling was set to 10,000 sequences per sample for the simulated and artificial datasets. All commands specific to each pipeline are detailed in Additional file 5: Doc S2. All reads (or representatives of OTUs) were taxonomically assigned using the SILVA r132 database. The runs were processed using a Galaxy instance of the Toulouse Midi-Pyrenees GenoToul bioinformatics platform to run FROGS pipeline, and a Ubuntu-based system (Ubuntu 18#04, Intel(R) Xeon(R) CPU E5-2640 v4 @ 2.40 GHz) to run both BIOCOM-PIPE and mothur pipelines.

### Statistical analyses

Statistical analyses were performed with R software (v.3.5.1). Alpha-diversity (OTU richness, Shannon diversity and Inverse Simpson indexes) and beta-diversity (Bray–Curtis dissimilarity index) were calculated. The microbial beta-diversities were estimated from the abundance of OTU-based Bray–Curtis distances and visualized using non-metric multidimensional scaling (NMDS) with the *metaMDS* function from the vegan package (v2.5–6) [44]. To determine the relationship between the global microbial community structures based on Bray–Curtis matrices, Mantel tests were performed with 999 permutations for the tests of significance based on Pearson’s rank correlation. Non-parametric PERMANOVA tests were run with *adonis* function (permutations fixed to 999) present in the vegan package, and also multilevel pairwise comparisons between land use with the wrapper function *pairwise Adonis* (https://github.com/pmartinezarbizu/pairwiseAdonis) [45]. To check the homogeneity of dispersions (variances) when comparing land use groups, a multivariate test was applied using *betadisper,* and supplemented with a permutational test to get a test statistic using *permutest* [46].

## Results

### Analysis of simulated and artificial datasets

The simulated dataset was constructed to be close to a realistic scenario [39], this is why they randomly mutated 2% of positions of each 16S rRNA sequence retrieved after extracting the needed sub-region. Firstly, it is noteworthy that some samples did not pass all filters with FROGS, mainly due to its approach for chimera detection (Additional file 6: Figure S4 A). The results after chimera detection, normalization and OTUs definition are close for the majority of samples, whatever the pipeline (Additional file 6: Figure S4 A), except FROGS, with a higher richness with a clustering threshold at 95%. For example, the total number of OTUs for samples with low diversity are close to 100 OTUs, whatever the defined similarity threshold. Taxonomic assessment results indicated also that the pipeline type had little influence (Additional file 6: Figure S4 B), whatever the sequencing depth, the clustering threshold, or the sample origin. Altogether, the observed diversity pattern reflects the two levels of diversity defined in silico for each environment, whatever the pipeline.

The artificial dataset composed of eight bacterial and two fungal strains, encompassed 179 sequences of 16S, for a total of 10 OTUs at 95% of similarity expected (data not shown). Firstly, a high number of chimeras are detected, whatever the pipeline (Additional file 7: Figure S5 A). Mothur detected the highest number of chimeras, followed by FROGS, and BIOCOM-PIPE (with the hunting step only). After normalization and removal of singletons, the observed richness was higher than the expected one, whatever the pipeline (Additional file 7: Figure S5 B). More precisely, the number of OTUs was close to 30 for FROGS, followed by mothur (ranging from 60 to 90 OTUs) and BIOCOM-PIPE (ranging from 100 to 127 OTUs). Taxonomic assessment results indicated firstly differences between the two independent runs, and secondly that FROGS was unable to detect some strains (from *Listeria* and *Enterococcus genera*), contrary to BIOCOM-PIPE and mothur (Additional file 7: Figure S5 C). Moreover, some *genera* were more detected with BIOCOM-PIPE and mothur (*i.e. Bacillus* or *Lactobacillus*) and with FROGS (*i.e. Staphylococcus*), or detected with a very low abundance compared to the theoretical one (*i.e. Salmonella* or *Escherichia*).

### Computational resource requirement and processing time of the real dataset

In terms of resource requirements and processing time, it is obvious that the ASV definition was the most efficient, compared to mothur or BIOCOM-PIPE. Then, as FROGS was available using a Galaxy specific instance of the Toulouse Midi-Pyrenees GenoToul bioinformatics platform, it was faster to treat the whole dataset, but difficult to compare its processing time to other pipelines. Mothur needed less processing time (10 h) than BIOCOM-PIPE (30 h), mainly due to the INFERNAL alignment and the ReClustOR post-clustering steps.

### Chimera detection, clustering and OTU composition of the real dataset

**Fig. 3:**
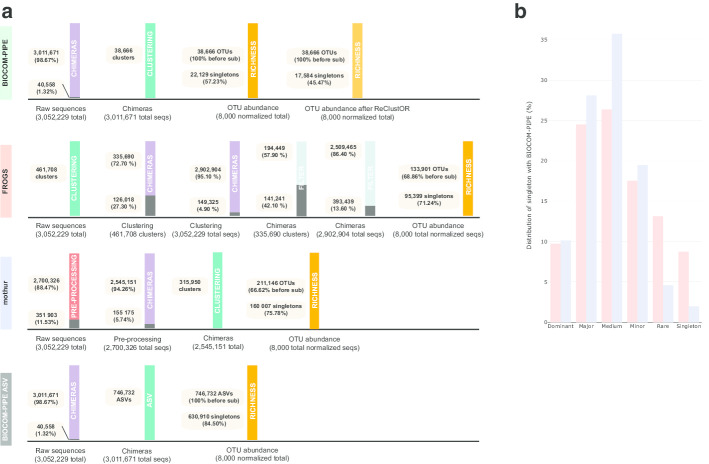
**a** Schematic benchmarking of the main steps of metabarcoding pipelines using an RMQS subsample. For all pipelines, purple color represents the chimera removal step, green represents the clustering step (or the ASV definition for BIOCOM-PIPE ASV), and dark orange represents ASV/OTU richness after rarefying the number of sequences for each sample to the smallest sample size. For BIOCOM-PIPE, the light orange color represents the specific post-clustering step. For FROGS, the light blue color represents a specific filtering step. For mothur, the red color represents the pre-processing step. Finally, the dark gray color represents the sequences to be removed for the next step. **b** Distribution of singletons defined with FROGS and mothur into OTUs defined with BIOCOM-PIPE. All singletons defined with FROGS (in pink) and mothur (in blue) were checked to define to which class of OTU they were associated with BIOCOM-PIPE. Six classes of OTUs were defined (dominant > 0.5% of the total sequencing depth; 0.5% > medium ≥ 0.05%; 0.05% > minor ≥ 0.005%; 0.005% > rare ≥ 0.0005%; rarest > 0.00005%; and singletons with one sequence into one OTU)

The 180 selected samples contained 3,052,229 total sequences after the preparation of raw sequences for further benchmarking analysis (Figs. 2, 3). The results of the chimera detection step indicated that the mothur approach removed more sequences (5–6% of the total dataset) than the homemade approach from BIOCOM-PIPE (around 1% of total sequences). For the FROGS pipeline, the clustering step was performed before chimera deletion, and around 30% of OTUs were removed (Fig. 3a).

The total clusters differed depending on the clustering approach. The ASV definition produced the highest number of clusters (746,732), followed by FROGS (461,708), mothur (315,950) and BIOCOM-PIPE (38,666) (10 times less than FROGS) (Fig. 3a).

**Fig. 4:**
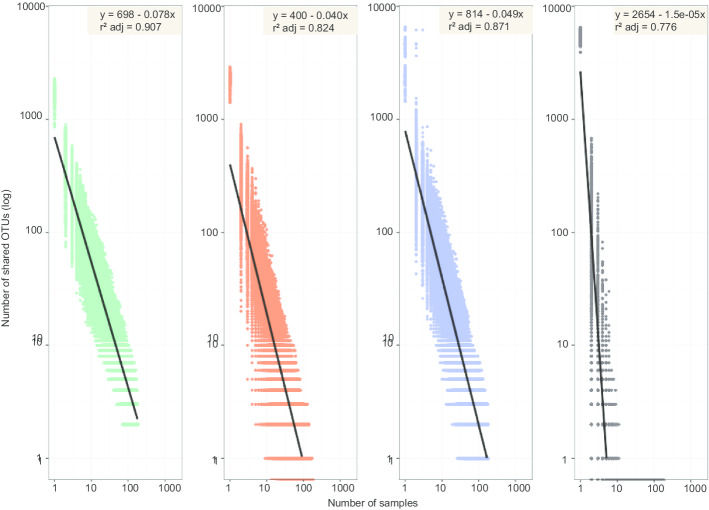
Number of OTUs shared by a given number of samples according to **a** BIOCOM-PIPE, **b** FROGS, **c** mothur, **d** ASV (BIOCOM-PIPE). The distribution is shown on a logarithmic scale on the two axes

Following the various steps specific to each pipeline and the subsampling step to normalize the samples based on the smallest sample, OTU richness greatly varied depending on the tools. It is important to note that the smallest sample would have enabled us to set subsampling to 10,000 sequences per sample with BIOCOM-PIPE. However, we could only set it 8000 sequences with the other two pipelines, so we kept that value to compare the pipelines. OTU richness was greatest with mothur (211,146 OTUs), then FROGS (133,901 OTUs) and finally BIOCOM-PIPE (38,666 OTUs) (Fig. 3a). The mean number of OTUs per sample was 2289 (min = 1448; max = 3257; delta = 1809) with mothur, 2182 (min = 1418; max = 2908; delta = 1490) with FROGS, and 1641 (min = 858; max = 2293; delta = 1435) with BIOCOM-PIPE. We also studied the number of singletons among total OTUs. BIOCOM-PIPE had the lowest number of singletons after the ReClustOR step, (around 45% of total sequences) versus around 70% and 75% for FROGS and mothur, respectively (Fig. 3a). Indeed, a high number of singletons defined with FROGS and/or mothur were reassigned to higher OTUs with BIOCOM-PIPE (Fig. 3b). Only 9% of singletons were shared between FROGS and BIOCOM-PIPE, and 2% between mothur and BIOCOM-PIPE. Furthermore, BIOCOM-PIPE found more shared OTUs than mothur, FROGS or the ASV did when the number of samples increased (Fig. 4).

### Alpha-diversity analysis of the real dataset

**Fig. 5:**
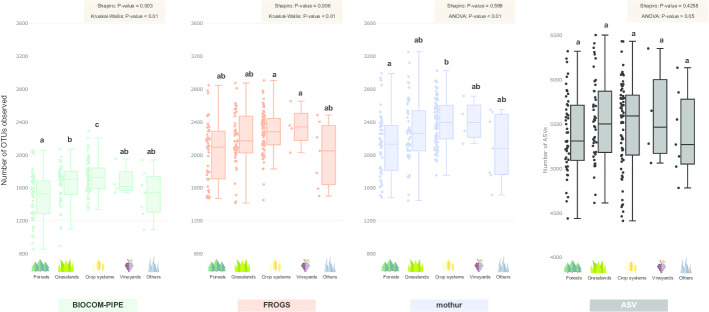
Boxplots of OTU richness observed for each land use type of the RMQS subsample. The samples were rarefied at 8000 sequences for FROGS, mothur, BIOCOM-PIPE and ASV. The circles represent the different samples for each land use group. Letters above boxplots represent pairwise comparisons between land use types using a wrapper function of *adonis* (~ Permanova) from the 'vegan' package

A significant difference between land uses was observed between the pipelines regarding OTU richness and Inverse Simpson metrics, whereas no difference was observed between FROGS and mothur regarding the Shannon index metric (Fig. 5, Additional file 8: Figures S6–S7). Land uses were compared independently within each pipeline in order to identify their potential effects (Fig. 5, Additional file 8: Figures S6–S7). The number of OTUs was smaller in BIOCOM-PIPE, but it allowed for a better discrimination of groups (forests < grasslands < crop systems < vineyards ≤ “others”) compared to other pipelines (Fig. 5). The Shannon index showed no significant difference between land use whatever the pipeline (Additional file 8: Figure S6). Lastly, as regards the 1/Simpson index, only FROGS and ASVs discriminated some of the defined groups (Additional file 8: Figure S7).

### Beta-diversity analysis of the real dataset

**Fig. 6:**
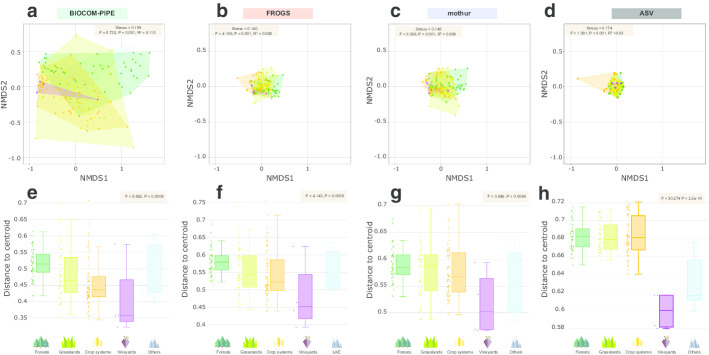
Non-metric multidimensional scaling of the RMQS subsample dataset using Bray–Curtis dissimilarities according to **a** BIOCOM-PIPE, **b** FROGS, **c** mothur, and **d** ASV definition, and boxplots of distances to centroids according to **d** BIOCOM-PIPE, **e** FROGS, **f** mothur, and ASV definition (**g**). Stress values between 0.1 and 0.2 provide useable representations. Colors refer to five different land use types: dark green = forest; light green = grasslands; orange = crop systems; purple = vineyards; light blue = “others”. Significance codes for each treatment were assigned based on the results of pairwise comparisons using adonis (Additional file 7: Table S2)

The NMDS stress values ranged between 0.109 (BICOM-PIPE) and 0.174 (ASV); therefore, it was possible to use community structure results to compare land use groups whatever the pipelines. The resulting NMDS ordination highlighted that bacterial community samples were more scattered and discriminated the land use gradient better under BIOCOM-PIPE (Fig. 6a–d). Mantel tests indicated that all microbial structures were strongly correlated, with r values ranging between 0.87 and 0.94 and *P* value < 0.01, except with the ASV definition (Additional file 9: Table S1). The permutational multivariate analysis of variance using distance matrices showed a significant difference between land use types whatever the pipeline (ADONIS, *P* value < 0.001) (Fig. 6). More precisely, all three pipelines highlighted significant differences between crop systems and grasslands, crop systems and forests, and crop systems and “others”, as well as between grasslands and forests (Additional file 10: Table S2). The ASV definition demonstrated the lowest differences between land uses (Additional file 10: Table S2). BIOCOM-PIPE distinguished significant differences between grasslands and vineyards (adjusted *P* value < 0.05) (Additional file 10: Table S2). Furthermore, significant differences and identical hierarchy between land use groups were observed for the distance-to-centroid variance with all pipelines (Fig. 6e–h).

### Taxonomic composition of the real dataset

**Fig. 7:**
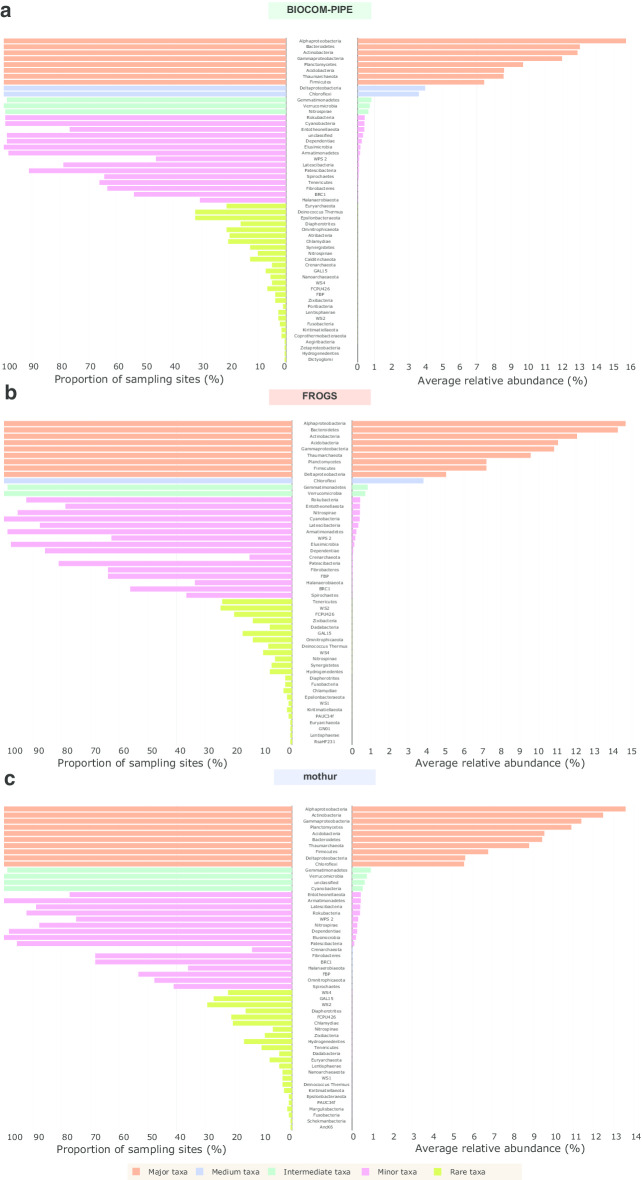
Representativeness of the soil bacteria and archaea from an RMQS subsample according to **a** BIOCOM-PIPE, **b** FROGS, and **c** mothur. Left: proportion of sampling sites where the phyla were present; right: average relative abundances of the phyla. The five groups were calculated by ascendant hierarchical clustering. ASV definition was equivalent to BIOCOM-PIPE (**a**), as all sequences are taxonomically assigned

Taxonomic assessment results with the representation of phyla in sampling sites and their relative abundances indicated that the pipeline type had little influence (Fig. 7). The total numbers of phyla were around 54, 51 and 52 for BIOCOM-PIPE (similar to ASV definition, as all sequences were affiliated), FROGS and mothur, respectively (unclassified phylum excluded) (Additional file 11: Table S3). Alphaproteobacteria was the most abundant phylum for the three pipelines; as regards the major taxa, the very same phyla were found, but were ranked differently (Fig. 6). Mothur assigned no phylum to the medium group, while all sequences were assigned with FROGS, and 0.31% and 0.62% were left unclassified by BIOCOM-PIPE and mothur, respectively (Fig. 7).

## Discussion

BIOCOM-PIPE is a new pipeline designed to characterize microbial diversity from environmental DNA metabarcoding data. Faced with many tools already available, we chose to work closely with various stakeholders (biologists, computational scientists, statistics experts) to suggest alternative approaches to reduce errors and biases from metabarcoding datasets. This expertise is based on 10 years of work aimed at developing standard operating procedures for molecular preparation and sequencing in close collaboration with the GenoSol platform [47]. BIOCOM-PIPE was designed to remain manageable for both beginners and advanced users. Once the fully completed “project file” and the raw datasets are placed in the appropriate folder, the pipeline can be run directly with all default parameters, or with specific parameters defined by an advanced user.

BIOCOM-PIPE incorporates both existing tools and homemade programs. For example, chimera detection (called the "hunting-recovering" step) is based on the search of non-clustered low-abundance sequences, and checks their reliability based on taxonomic assignment. The chimera formation from complex DNA amplification is not random, as it is a function of many factors such as template abundance, sequence homology, enzyme accuracy, PCR conditions, etc. [26, 48, 49]. Moreover, chimeras are more often generated among richer, phylogenetically diverse samples [50], but co-extract products and inhibitors can also influence chimera formation. A mock community should be used to find the best bioinformatic parameters to minimize errors from the chosen procedure (DNA extraction, PCR amplification, sequencing, etc.), but too stringent filters can lead to the loss of rare microorganisms [26, 48, 49, 51]. Here, FROGS seems to be the more efficient to form the lowest number of OTUs, but it was unable to detect some strains, as one representative sequence per OTU is selected and taxonomically assigned after clustering, leading to biases in community composition [49]. BIOCOM-PIPE and mothur showed better results in terms of taxonomic affiliation. It is noteworthy that BIOCOM-PIPE can be more flexible, as it is possible to optimize the "hunting-recovering" parameters to adapt the stringency of the detection, leading to the deletion of the “best” potential chimera.

Moreover, the latest taxonomic database updates for Archaea, Bacteria and Fungi are also available from SILVA r132, and µgreen-db for photosynthetic microeukaryotes and cyanobacteria. The classical approach for the clustering step is to first align sequences with the reference alignment. We propose to use both RNA structure and sequence similarities with an approach based on a covariance model. Sequence alignment using this method allows for better OTU assignment [52] and reduces computational time and required computer memory [53]. For data potentially containing a high number of homopolymers, global alignment based on the secondary structure showed better sensitivity to detect homopolymer errors. The question of reproducibility and robustness in microbiota analyses is more and more significant in microbial ecology [54]. BIOCOM-PIPE was specifically designed to tackle some of these challenges. Indeed, although the use of a “project file” may appear as a constraint at first sight, users are forced to start the analysis by supplementing it. This will make it easier for supervisors, colleagues or users themselves to choose the parameters of the analysis. Moreover, having a structured folder with all summary results and parameters allows for better archiving.

It is currently very difficult to compare studies carried out with different bioinformatic analyses [2]. To address this issue while providing great flexibility, a post-clustering step using ReClustOR was added to BIOCOM-PIPE, compared to classical pipeline analysis [17]. In order to overcome the problems of OTU stability and reliability posed by the classical clustering methods, this post-clustering step was implemented to improve the quality of the reconstructed OTUs. Moreover, thanks to an available open-reference clustering method, ReClustOR can define and/or enrich a reference OTU database with analyzed projects without carrying out the complete analysis each time new sequences are added. As a consequence, such a post-clustering step is a clear benefit that increases the reproducibility and reliability of results among different clustering analyses, and also between samples and datasets [17].

To highlight BIOCOM-PIPE specificities compared to other pipelines, a subsampled dataset from the RMQS metabarcoding project was used [13]. The key steps to reduce the number of sequences vary between pipelines, especially during the chimera step. At the end of the analysis, BIOCOM-PIPE kept the highest number of sequences for the subsampling step, which made it possible to set this step to 10,000 sequences versus 8000 with FROGS or mothur. A higher number of OTUs was observed with mothur, in line with previous studies [7]. It is worth noting that BIOCOM-PIPE counted the lowest number of singletons thanks in particular to the use of ReClustOR, which allowed for better sequence assignment. Moreover, some sequences considered to be rare in the biosphere by certain pipelines were not [17]. In the same line, BIOCOM-PIPE showed that some OTUs were better represented in the samples, with more shared OTUs. Interestingly, the ASV definition did not show significant differences between land uses, contrary to other clustering methods. This can be explained by the high-diversity of analyzed samples. But, the use of the ASV definition, using dedicated tools such as DADA2 [41], in combination with OTUs computation can be especially informative to detect shifts in the microbial community that the OTU definition has overlooked. Altogether, clustering approaches are clearly complementary to the ASV definition, as different kind of information is given by both methods.

Regarding taxonomic assignment, BIOCOM-PIPE seemed to show a more detailed degree of information with more phyla. Nevertheless, it was somewhat reassuring to find almost the same phyla for the major groups representing 90% of relative abundance. Chloroflexi was not in the major group for BIOCOM-PIPE and FROGS, but in the medium group. Furthermore, Deltaproteobacteria were present in the major group for FROGS and mothur but in the medium group for BIOCOM-PIPE. These differences could be explained by the divergent approaches of taxonomic affiliation used by the pipelines. Another positive aspect, although at first sight the community structure seemed different with variable OTU distances to the centroid, was that Mantel test also showed good correlation for sample paired-comparison between pipelines.

From an ecological point of view, BIOCOM-PIPE discriminated land uses based on OTU richness much better than FROGS and mothur. Indeed, the land use hierarchy due to a gradient of practice intensity was similar to what was previously observed and described [17]. Altogether, even if the results were different depending on the bioinformatic pipeline, the same ecological interpretation was found. Different studies compared various pipelines and also concluded to the same trends [55, 56].

For future improvements, it would be important to add a new correction step based on the gene copy number, which differs between organisms [57]. This method is increasingly used, although controversial due to low predictive accuracy compared with completely sequenced genomes [58], and it has been reported to improve estimates of microbial diversity [59]. Moreover, due to the growing number of complete microbial genomes available, this method can now be more accurate [60].

Another improvement can be achieved by providing an alternative method to characterize fungal communities by analyzing the internal transcribed spacer (ITS). Therefore, it will be necessary to integrate another aligner than Infernal (e.g., MAFFT program [61]) for multiple sequence alignment since no covariance model for fungal ITS sequences exists. Finally, if we keep focusing on linking sequencing preparation and bioinformatic analysis more closely, it would also be interesting to integrate a component to check the validity of sequencing runs and the different pipeline steps. About run effectiveness, an upstream analysis of internal standards with various tests (e.g., richness, community structure, taxonomy) could be used to validate or not the sequenced samples. An internal standard reference database could be created, compared and enriched to highlight a drift. A second checking could be performed for each bioinformatic analysis to verify the proper functioning of the different steps with the previous analysis by using a mock community sample for example. This could be very useful when multiple updates are conducted on various components and by different developers.

## Conclusions

Although many complex questions remain unanswered concerning microbial ecology [62], tremendous advances have been made from a technical point of view, with the constant emergence of new protocols or sequencers, but also better knowledge of microbial diversity. This craze boosted scientists to develop algorithms and tools to analyze metabarcoding data over and over again. BIOCOM-PIPE is an interesting alternative for the scientists who wish to benefit from an efficient and flexible tool. This pipeline is robust, based on longstanding expertise, and can compare large datasets with great ease. Its original features use various rarely used methods or tools, not because there are not effective but likely due to a lack of hindsight. Like any tool, it is constantly evolving and may also contain errors. We thank readers and users for informing us about any errors or suggestions likely to improve this tool.

## Availability and requirements

BIOCOM-PIPE package is available in Zenodo repository (https://doi.org/10.5281/zenodo.3678129).

A virtual machine encompassing the complete pipeline into an UBUNTU system is also available (https://doi.org/10.5281/zenodo.3945964).

All raw datasets for the example are publicly available in the EBI database system (in the Short Read Archive) under project accession No. PRJEB21351.

Project name: BIOCOM-PIPE.

Project home page: Not defined.

Operating system(s): Linux and UNIX.

Programing language: Perl, Python, C, JavaScript, HTML/CSS.

Other requirements: all third-party tools listed in the User Guide.

License: CC.

Any restrictions to use by non-academics: Invention disclosure statement (DI-RV-17-0037).

## Supplementary information

**Additional file 1.** Doc S1. BIOCOM-PIPE user guide.

**Additional file 2.** Figure S1. Folder structure of the BIOCOM-PIPE pipeline.

**Additional file 3.** Figure S2. Folder structure of BIOCOM-PIPE analysis.

**Additional file 4.** Figure S3. Soil texture triangle of the RMQS subsample.

**Additional file 5**. Doc S2. Materials and methods, details.

**Additional file 6.** Figure S4. (A) Number of OTUs detected for the simulated dataset, with various pipelines and clustering thresholds. (B) Number of taxonomic groups detected for each taxonomic level (phylum, class, order, family, genus), with various pipelines and clustering thresholds.

**Additional file 7.** Figure S5. (A) Number of detected chimera for the artificial mock dataset, with various pipelines. For BIOCOM-PIPE, both the Hunting step (H) and the Hunting-Recovering step (R) were given. (B) Number of OTUs (with and without singletons) detected, for the artificial mock dataset, with various pipelines. (C)Taxonomic composition for the artificial mock dataset, with various pipelines. The theoretical value was given as comparison.

**Additional file 8.** Figure S4. Boxplots of Shannon indexes for each land use type of the RMQS subsample. Figure S5. Boxplots of the inverse Simpson indexes for each land use type of the RMQS subsample.

**Additional file 9.** Table S1. Correlations between two “Bray–Curtis” distances of bacterial community composition using Mantel test.

**Additional file 10.** Table S2. Results of pairwise ADONIS testing for the effect of land use type on variation in microbial structure. ADONIS signif. codes: 0 ‘***’; 0.001 ‘**’; 0.01 ‘*’; 0.05 ‘.’.

**Additional file 11.** Table S3. Number of phyla per pipeline for each group.

## Data Availability

All raw data are publicly available in the EBI database system under project accession PRJEB21351 for the RMQS dataset. The raw dataset used as example is available in the EBI database system under project accession number PRJEB14258. The associated demo input files and expected output files from the pipeline are publicly available in the Zenodo repository: 10.5281/zenodo.3947784.

## Abbreviations

ReClustOR: A re-clustering tool using an open reference
RNA: Ribonucleic acid
OTU: Operational taxonomic unit
RMQS: réseau de mesures de la qualité des sols
NMDS: Non-metric multidimensional scaling
PERMANOVA: Permutational multivariate analysis of variance

## Footnotes

1 The GenoSol platform initially developed GnS-PIPE, devoted to the conservation and analysis of the genetic resources of soil microbial communities [46].
