# Supplementary material for: BIOCOM-PIPE: a new user-friendly metabarcoding pipeline for the characterization of microbial diversity from 16S, 18S and 23S rRNA gene amplicons
Source: BMC Bioinformatics. 2020 Oct 31;21:492. doi: 10.1186/s12859-020-03829-3 (PMC7603665; doi:10.1186/s12859-020-03829-3)
Supplement: Supplementary file 1 — Additional file 1. Doc S1. BIOCOM-PIPE user guide. [file 12859_2020_3829_MOESM1_ESM.pdf]

BIOCOM-PIPE User’s Guide

A new user-friendly metabarcoding pipeline for the characterization of microbial diversity from 16S, 18S, 23S rRNA genes amplicons

Zenodo DOI : 10.5281/zenodo.3678129

Version 1.19.0; Jan 2020

Christophe DJEMIEL and Sébastien TERRAT
for the BIOCOM-PIPE development team

Contents

Contents iii

List of Figures v

1 Introduction 1

1.1 What is metabarcoding ? . . . 1

2 Simplified description of the modules 3

2.1 PRINSEQ . . . 3
2.2 FLASH . . . 4
2.3 EVAL_QUAL . . . 5
2.4 PREPROCESS_MIDS . . . 6
2.5 RANDOM_PREPROCESS_MIDS . . . 6
2.6 PREPROCESSING . . . 7
2.7 QUALITY_CLEANING . . . 8
2.8 RANDOM_PREPROCESS . . . 9
2.9 LENGTH_RANGE_PREPROCESS . . . 10
2.10 RAW_INFERNAL_ALIGNMENT . . . 10
2.11 FILTERING_RAW_ ALIGNMENT . . . 11
2.12 RAW_CLUSTERING . . . 12
2.13 HUNTING . . . 14
2.14 RECOVERING . . . 14
2.15 RANDOM_CLEANED . . . 16
2.16 TAXONOMY . . . 16
2.17 CLEAN_INFERNAL_ALIGNMENT . . . 18
2.18 CLEAN_CLUSTERING . . . 19
2.19 COMPUTATION . . . 21
2.20 GLOBAL_ANALYSIS . . . 22
2.21 RECLUSTOR . . . 23
2.22 UNIFRAC_ANALYSIS . . . 25

3 Installation 27

3.1 Quick installation instructions . . . 27
3.1.1 System requirements . . . 27

3.1.2 INSTALLATION OF BIOCOM-PIPE 27
3.1.3 INSTALLATION OF THIRD-PARTY TOOLS 27

4 Tutorial **35**
4.1 Dataset 35
4.2 Creating a pipeline analysis 35
4.2.1 Preparation files 35
4.2.2 Launch analysis 36
4.2.3 Create figures 37
4.3 How we can use the recent/ custom database ? 41
4.3.1 Databases organization 41
4.3.2 Addition of a new database organization 42
4.3.3 Program files that can be impacted by a new/custom database integration 42

5 Some other topics **45**
5.1 How do I cite 45
5.2 How do I report a bug? 45

Bibliography **47**

List of Figures

2.1 Example of homopolymers 12
2.2 Schematic view of classical greedy clustering and ReClustOR approaches. 24

4.1 Example of a project file 35
4.2 Example of a Input.txt generated 36
4.3 Local website - Home page 37
4.4 Local website - Parameters page 37
4.5 Local website - Information for Prinseq tool 38
4.6 Local website - Diagram of a hierarchical directory tree of your pipeline. 38
4.7 Local website - Summary files page 38
4.8 Local website - Evaluation of quality from Summary files page 39
4.9 Local website - Rank abundance graphs from alpha-diversity page . . . 39
4.10 Local website - Unifrac tree graphs from beta-diversity page 40
4.11 Local website - Taxonomy page 40
4.12 Databases organization 41

Introduction 1

1.1 What is metabarcoding ?

Meta-barcoding (or targeted meta-genomics) is a technique based on a PCR approach targeting a DNA fragment common to all the organisms studied (commonly called a « molecular marker »). The latter can be a gene making it possible to determine the family ties between organisms (the gene is then considered as a taxonomic marker), or a gene with a specific function (we are then interested in the functional potential of the communities studied, ie is a functional marker).

Simplified description of the modules

2

2.1 PRINSEQ

```
###PRINSEQ###
Step to do [yes-no]:        no
Lowest quality score tolerated for the trimming from the 3'-end of the read [0-40]:        30
Lowest quality score tolerated for the trimming from the 5'-end of the read [0-40]:        30
Minimum Length threshold tolerated to keep reads (default: 30):        30
Number of ambiguities (N's) tolerated (default: 1):        1
Sliding window size used to calculate quality score [1-7]:        7
Step size used to move the sliding window [1-10]:        1
//
```

This step is based on a third-party-tool called PRINSEQ [Schmieder and Edwards, 2011]. This tool is dedicated to filter sequence datasets (from 454 or ILLUMINA sequencing technologies) to remove sequence copies, short or long sequences, sequences with N's, low-quality sequences, trim sequences and much more.

As indicated in the Input.txt file, this step needs several input parameters given by the user. The first and the second ones are used to define the quality score thresholds for both 3' and 5' trimming-end of the sequences (corresponding to *-trim_qual_right* and *-trim_qual_left* options in PRINSEQ). If the evaluated quality score is below this defined threshold, the sequence will be truncated by the PRINSEQ program. The third parameter defined the minimum length of the sequence to keep them after trimming (corresponding to the *-min_len* option in PRINSEQ). If the sequence length (after trimming) is below this threshold, the sequence itself will be deleted. The fourth parameter indicated the number of ambiguities tolerated in the sequences (corresponding to the *-ns_max_n* option in PRINSEQ). The two last parameters are used to define the sliding window size used to calculate quality score on sequences, and also the step size used to move the sliding window (corresponding to *-trim_qual_window* and *-trim_qual_step* options in PRINSEQ).

This step, after sequence analysis, will produce a summary file for each sequence file (or paired-end files) in the Summary_files folder with all details (number of kept sequences, deleted sequences, trimmed sequences, etc.). Moreover, a FASTQ file (or two if paired-end files were treated) will be stored in the Result_files/PRINSEQ/ folder containing cleaned sequences. It is noteworthy that the BIOCOM_PIPE program will automatically detect if paired-end files are given or not, to treat them efficiently for further steps (and more particularly for the FLASH step if needed).

What do you need?

This step was developed in the PerlLib::FastQTreatment.pm library, but also needs Miscellaneaous.pm library, File::Copy library installed, and also the prinseq-lite.pl program in the folder Data/Tools/PRINSEQ/.

2.2 FLASH

```
###FLASH###
Step to do [yes-no]:        no
Minimum overlap length between two reads (default: 10) [10-100]:        10
Maximum overlap length between two reads (default: 65) [10-100]:        65
Maximum allowed ratio between the number of mismatched base pairs and the overlap length (default: 0.04) [0-100]:        0.04
//
```

This step is based on a third-party-tool called FLASH [Magoč and Salzberg, 2011]. FLASH (Fast Length Adjustment of SHort reads) is a very accurate and fast tool to merge paired-end sequences (from ILLUMINA sequencing technologies for example) that were generated from DNA fragments whose lengths are shorter than the length of sequences.

As indicated in the Input.txt file, this step needs several input parameters given by the user. The first and the second ones are used to define the minimum required overlap length between two reads (corresponding to *-m* option in FLASH) and the maximum required overlap length between two reads (corresponding to *-M* option in FLASH), both used to provide a confident overlap. It is noteworthy that overlaps longer than the maximum threshold is still considered as good overlaps, but the mismatch ratio (explained after) is calculated over this threshold, rather than the true overlap length. On the contrary, a sequence with an overlap below the minimum threshold will be deleted. The third parameter is used to define the 'ratio' (corresponding to *-x* option in FLASH). This ratio is the maximum allowed ratio of the number of mismatches and the overlap length. An overlap with mismatch ratio higher than the set value is considered incorrect overlap and mates will not be merged. Any occurrence of an ambiguity (or 'N') in any read is ignored and not counted towards the mismatches or overlaps length. For example, for an overlap of 10 bases, with a ratio defined to (0.04), 0.4 bases of mismatches are tolerated (rounded down), so 0 mismatch. For an overlap larger than 65 bases (for example 100 bases), the number of mismatches is computed using the maximum required overlap length defined (by default: 65 bases). So, 65 x 0.04 = 2.6 bases (rounded down), so 2 bases. To conclude, even with overlaps longer than the maximum threshold, the number of mismatches will be computed with the defined values, increasing the stringency of the merging.

This step, after sequence analysis, will produce a summary file for each sequence file (or paired-end files) in the Summary_files folder with all details (number of merged sequences, deleted sequences, etc.). Moreover, a FASTQ file will be stored in the Result_files/FLASH/ folder containing cleaned sequences.

What do you need?

This step was developed in the PerlLib::FastQTreatment.pm library, but also needs Miscellaneaous.pm library, File::Copy library installed, and also the flash program in the folder Data/Tools/FLASH/.

2.3 EVAL_QUAL

```
###EVAL_QUAL###
Step to do [yes-no]:        no
Length:        300
//
```

This step is close to the FastQC program dedicated to the quality control of high throughput sequence data. Based on the quality information given by the sequencing technology (from files .qual, .sff, or .fastq), it provide a simple summary of quality to do some control checks on raw sequence datasets.

As indicated in the Input.txt file, this step needs one input parameter given by the user: the length threshold of analyzed sequences. If sequences are shorter than this threshold, they will not be considered by the program.

This step, as output, produce a summary file for each file (or paired-end files) in the Summary_files folder. This file will describe the quality of analyzed sequences (minimum, maximum, median and average) using a window size of 25 bases, but also the number of sequences with quality score between 40 and 35, between 35 and 30, between 30 and 25, or below 25.

What do you need?

This step was developed in the PerlLib::ManagingQuality.pm library, but also needs the Miscellaneaous.pm library.

2.4 PREPROCESS_MIDS

```
###PREPROCESS_MIDS###
Step to do [yes-no]:         no
Filename/MID/Sample:         IIYLCB301/MID131/MID131/CRTL_G4
Filename/MID/Sample:         IIYLCB301/MID168/MID131/Naiz2013_002
Filename/MID/Sample:         IIYLCB301/MID129/MID131/Naiz2013_004
//
```

This step is also known as the demultiplexing step, done with high-throughput sequencing technologies, like 454 or Illumina. It takes as input many format files (.fasta, .fna, .sff, or .fastq. files), and need the complete description of each library. More precisely, as input, the user must describe each sample in the library with one line containing several elements separated by "/" characters: the name of the library, the name (or the sequence) of the Multiplex Identifier (or MID) used in the 5' position, the name (or the sequence) of the MID used in the 3' position, and finally the chosen ample name for further analyses. No special characters (e.g. spaces, ":", etc.) are allowed for sample names.

A summary file is produced at the end of this step in the Summary_files folder. This file describes the total number of sequences analyzed from the library, and the number of sequences associated to each MID, and consequently to each sample. This step produced also a FASTA file and a quality file for each sample in the dedicated subfolder Result_files/PREPROCESSING_MIDS. As multiple libraries can be analyzed, several libraries can be treated in parallel, and the number of samples to be treated is not limited. However, the unicity of sample names and used MIDs are checked by the program for each library, and the program will be stopped if needed.

What do you need?

This step was developed in the PerlLib::Demultiplexing.pm library (more details inside the code itself), and needs also the Miscellaneaous.pm library.

2.5 RANDOM_PREPROCESS_MIDS

```
###RANDOM_PREPROCESS_MIDS###
Step to do [yes-no]:         no
Number of reads in the subset:         300
//
```

This step was developed to allow the user to homogenize the treated dataset from various sequences files (.fasta, .fna, .sff, or .fastq). More precisely, it takes as input the number of sequences needed for each sample. Based on this input number, the

program will select randomly the needed number of sequences for each independent sample. If the initial number of sequences in a sample is below the wanted number of sequences, the program will copy the file without random selection.

A summary file is produced at the end of this step in the Summary_files folder. This file describes for each sample if the random selection was realized or not. This step produced also a FASTA file (and a quality file if needed) for each sample in the dedicated subfolder Result_files/RANDOM_PREPROCESSING_MIDS.

Note: only one random selection is authorized by the pipeline (this step can be done on samples after demultiplexing, after preprocessing or after all filters), as several subsampling steps can bias the analysis. So, if the user chose several random steps, the main program will terminate the analysis and indicate to the user the problem.

What do you need?

This step was developed in the PerlLib::RandomSelection.pm library (more details inside the code itself), and needs also the Miscellaneaous.pm library and the List::Util 'shuffle'.

2.6 PREPROCESSING

```
###PREPROCESSING###
Step to do [yes-no]:        no
Minimum Length threshold:        300
Number of ambiguities (N's) tolerated:        0
Forward primer:        CAGCMGCYGCNGTAANAC
Reverse primer:        CCGYCAATTCMTTTRAGT
Number of differences tolerated in your forward primer sequence [0-2]:        0
Number of differences tolerated in your reverse primer sequence [0-2]:        0
Stringency [High, Medium or Low]:        Medium
//
```

This step is dedicated to a first filtering or raw sequences (.fasta, .fna, and .qual or .fastq files) based on several parameters defined by the user. Indeed, the raw sequences can be filtered and deleted based on (i) their length, (ii) their number of ambiguities (Ns) and (iii) their primer(s) sequence(s). More precisely, this step takes several input parameters:

- The minimum length threshold of the sequence to keep them after preprocessing.
- The number of ambiguities (or 'N') tolerated in the sequences by the user. If a sequence contains more ambiguities, it will be deleted for further analyses.

- The sequences of the primer set used (forward and reverse primer). These sequences are based on the IUPAC code and can manage all degenerated bases. The order of the primers will be used by the main program to determine the direction of the sequence and to reverse-complement it if needed.
- The potential number of errors (mutation, deletion, insertion) in the sequences of the primers (forward and reverse) tolerated by the user.
- The analysis stringency required by the user (HIGH, MEDIUM or LOW). More precisely, this parameter indicates to the main program how the primer research will be done. The HIGH stringency keeps only sequences harboring perfect and complete sequences of both primers (considering previous parameters given by the user). The MEDIUM stringency keeps sequences harboring perfect and complete sequence of proximal primer, but the distal primer can be incomplete (with perfect matches of the incomplete sequence). Finally, the LOW stringency keeps sequences harboring at least a perfect and complete sequence of proximal primer, with a length higher than the defined threshold of the user. For example, the user chose the LOW stringency, each sequence will be evaluated first with a HIGH stringency, then a MEDIUM, and finally with a LOW stringency. So, each sequence will be cleaned of primer sequences if those sequences are found.

A summary file is produced at the end of this step in the Summary_files folder. This file describes for each sample how many sequences have been deleted by each parameter, and the number of sequences at the end of the analysis. This step produced also a FASTA file (and a quality file if needed) for each sample in the dedicated subfolder Result_files/PREPROCESSING.

What do you need?

This step was developed in the PerlLib::Preprocessing.pm library (more details inside the code itself), and needs also the Miscellaneaous.pm library.

2.7 QUALITY_CLEANING

```
###QUALITY_CLEANING###
Step to do [yes-no]:        no
Lowest quality score tolerated [0-40] (default: 0):        15
Lowest average quality score tolerated [0-40] (default: 0):        0
//
```

This step was developed to allow the user to clean his dataset (from .fasta and .qual files) based on the quality of the sequences using two parameters. The first parameter defined the lowest quality score tolerated on a sequence. If a base in a sequence

harbors a quality below this threshold, the sequence is deleted. The second parameter defined the lowest average quality score tolerated by the user on sliding window of 25 bases (used to calculate the average quality score). The sliding window is then moved on the sequence from one base. As previously, if sequence harbors an average quality below this threshold, the sequence is deleted.

A summary file is produced at the end of this step in the Summary_files folder. This file describes for each sample how many sequences have been deleted, and the number of sequences at the end of the analysis. This step produced also a FASTA file and a quality file for each sample in the dedicated subfolder Result_files/ QUALITY_CLEANING.

What do you need?

This step was developed in the Prog_Perl::Quality_cleaning.pl program (more details inside the code itself), and needs also the List::Util 'qw(min sum)' library.

2.8 RANDOM_PREPROCESS

```
###RANDOM_PREPROCESS###
Step to do [yes-no]:        no
Number of reads in the subset:        200
//
```

This step was developed to allow the user to homogenize the treated dataset from various sequences files (.fasta, .fna, .sff, or .fastq). More precisely, it takes as input the number of sequences needed for each sample. Based on this input number, the program will select randomly the needed number of sequences for each independent sample. If the initial number of sequences in a sample is below the wanted number of sequences, the program will copy the file without random selection.

A summary file is produced at the end of this step in the Summary_files folder. This file describes for each sample if the random selection was realized or not. This step produced also a FASTA file (and a quality file if needed) for each sample in the dedicated subfolder Result_files/RANDOM_PREPROCESS.

Note: only one random selection is authorized by the pipeline (this step can be done on samples after demultiplexing, after preprocessing or after all filters), as several subsampling steps can bias the analysis. So, if the user chose several random steps, the main program will terminate the analysis and indicate to the user the problem.

What do you need?

This step was developed in the PerlLib::RandomSelection.pm library (more details inside the code itself), and needs also the Miscellaneaous.pm library and the List::Util 'shuffle'.

2.9 LENGTH_RANGE_PREPROCESS

```
###LENGTH_RANGE_PREPROCESS###
Step to do [yes-no]:        no
Length:        300
//
```

This simple step was developed to evaluate the length distribution of sequences from the dataset. One parameter is needed, the minimum length of the sequences analyzed by the program.

This step will produce a file containing the number of sequences based on length ranges of the dataset in the Summary_files folder.

What do you need?

This step was developed in the Prog_Perl/Length_range_preproces_1.5.pl program (more details inside the code itself).

2.10 RAW_INFERNAL_ALIGNMENT

```
###RAW_INFERNAL_ALIGNMENT###
Step to do [yes-no]:        no
Covariance model used [bacteria, archaea, algae or fungi]:        bacteria
//
```

This step is dedicated to the definition of a global alignment against dedicated structures of rRNA (16S for bacteria and/or archaea, 18S for fungi, and 23S for plastidial microeukaryotic organisms) using the Infernal alignment program ('INFERence of RNA ALignment', v1.1.1) [Nawrocki and Eddy, 2013]. As input, the user must choose which group of microorganisms is studied, to use the best structure. Infernal was run chosen as a structural model allow for divergent but valid sequences to persist because stricter methods may incorrectly discard some sequences [Lynch and Neufeld, 2015]. Moreover, this alignment provides a more intuitive handling of sequencing errors, such as homopolymer errors that are easily detected thanks to the secondary-structure aware aligner. Therefore, such homopolymer errors can be

easily managed and ignored during the clustering step (see RAW_CLUSTERING or CLEAN_CLUSTERING steps for details).

This step produced a file containing aligned sequences (FASTA or STOCKHOLM format) for each sample in the dedicated subfolder Result_files/ RAW_INFERNAL_ALIGNMENT.

What do you need?

This step was developed in the PerlLib::InfernalAlignment.pm library (more details inside the code itself) and needs also the 'POSIX' library and the third-party-tool Infernal installed (V1.1.1), with direct launching of some commands like 'cmalign' or 'esl-alimerge'. Moreover, this program needs also the structures stored in the Prog_Perl/Matrix/ subfolder, and the Miscellaneaous.pm library.

2.11 FILTERING_RAW_ ALIGNMENT

```
###FILTERING_RAW_ALIGNMENT###
Step to do [yes-no]:        yes
//
```

This step is dedicated to the filtering of sequences based on the quality of alignment against the chosen rRNA structure (16S for bacteria and/or archaea, 18S for fungi, and 23S for plastidial microeukaryotic organisms) using the Infernal alignment program ('INFERence of RNA ALignment', v1.1.1) [Nawrocki and Eddy, 2013]. No parameter is needed by the user. Here, Infernal results were evaluated as a structural model allow for divergent but valid sequences to persist because stricter methods may incorrectly discard some sequences [Lynch and Neufeld, 2015]. Moreover, this alignment allows the recognition of sequences that are not considered as rRNA.

More precisely, the Infernal program give details about each aligned sequence:

Idx	seq_name	length	cm_from	cm_to	trunc	bit_sc	avg_pp	band_calc	alignment(sec)	total	mem(Mb)
A	I1HGXD1A	550	67	68	3'	-50.41	0.911	0.38	0.70	1.08	115.04
B	I1JCBQR0	371	474	835	5'&3'	422.03	1.000	0.09	0.01	0.11	5.93

These details are used to define if the sequence harbors a good structure or not. Here, the program checks if the 'bit_sc' value is positive (indicating the quality of the entropy of the alignment) or not. If the value is negative, the program considers that this sequence was not aligned (sequence A) against the chosen rRNA structure and therefore, the program will delete this sequence (keep sequence B, and delete sequence A).

A summary file is produced at the end of this step in the Summary_files folder. This file describes for each sample how many sequences were deleted. This step produced also

two files: one containing cleaned aligned sequences (FASTA or STOCKHOLM format) for each sample in the dedicated subfolder Result_files/ FILTER_RAW_ALIGNMENT, and the second file containing the details of the deleted sequences quality alignments, also in the dedicated subfolder Result_files/ FILTER_RAW _ALIGNMENT.

What do you need?

This step was developed in the Perl-Lib::FilteringInfernalAlignment.pm library (more details inside the code itself) and needs also the 'POSIX' library and the third-party-tool Infernal installed (V1.1.1), with direct launching of some commands like 'cmalign' or 'esl-alimerge'. Moreover, this program needs also the structures stored in the Prog_Perl/Matrix/ subfolder, and the Miscellaneaous.pm library.

2.12 RAW_CLUSTERING

```
###RAW_CLUSTERING###/Users/cdjemiel/Downloads/RMQS-bacteria/BIOCOM-PIPE_Paper/figures/
Step to do [yes-no]:        no
Ignoring homopolymers differences [yes-no]:        yes
Ignoring distances at the beginning of sequences [yes-no]:        yes
Maximum percentage of dissimilarity for clustering (%):        5.0
Step size for each cluster (%):        5.0
//
```

This step of analysis was developed to cluster analyzed sequences into OTUs using the globally aligned sequences with Infernal [Nawrocki and Eddy, 2013]. This program needs four parameters. The first parameter indicates if homopolymer differences will be considered as true differences or not. Homopolymer stretches can be problematic for several sequencing technologies (e.g. 454, IonTorrent, MinION, etc.). These sequencing platforms suffer from the inaccuracy in detecting the length of homopolymers repeats of the same nucleotide, so our program, using the global alignment, can detect such stretches (see example 2.1).

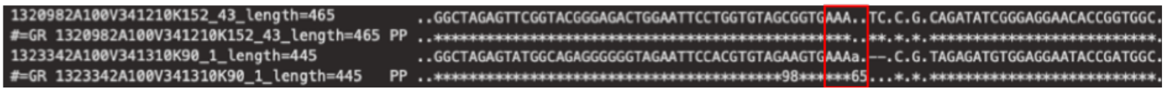

Fig. 2.1 – **Example of homopolymers**

Here, two sequences are compared by the clustering program after global alignment with Infernal. As Infernal is based also on the secondary structure of the rRNA, it can easily detect insertion-deletion errors originated from the sequencing step. As one of the two sequences has a high occurrence (here, 43 occurrences, indicated in its ID), the difference is ignored by the clustering program. Indeed, the sequence with

43 occurrences (after strict dereplication) can be considered as a "true" sequence. The difference with the other sequence (with only one occurrence) will be ignored during the clustering process. However, such differences cannot be detected on singleton sequences (if the two sequences have only one occurrence), as we are unable to confirm if there is a 'true biological' difference or not. Ignoring homopolymer differences can drastically reduce the number of defined OTUs (between 5 to 20%, depending on the origin of the sequencing, and the quality of the sequencing).

The second parameter allow the user to avoid the differences between two sequences harboring length differences. So, the program will consider only common parts of the sequences, and ignore specific parts of the analyzed sequences. The third and fourth parameters defined the maximum percentage of dissimilarity tolerated to create OTUs, and also the step size (if needed) to realize several clustering with different dissimilarity percentages. For example, if the maximum is defined to 5.0% de with a step size of 1.0%, the program will create a clustering with a threshold of 1% of dissimilarity, another with 2.0%, another with 3.0%, another with 4.0% and finally a last with 5.0% of threshold.

First of all, dereplicated sequences will be organized by decreasing abundance, and then they will be clustered based on the threshold chosen by the user. The clustering process is a is a greedy incremental clustering algorithm developed in C (introduced in PERL by the Inline specific module). The first sequence from the dataset becomes the seed of the first OTU (this first seed is also the most abundant sequence, and potentially the truest one). Then, the distance between the seed and each remaining sequence is compared. If the distance of the query to the seed sequence is equal or below a given threshold then it is assigned to that cluster. Otherwise, a new cluster is defined with that sequence as the seed.

A summary file is produced at the end of this step in the Summary_files folder. This file describes for each sample how many OTUs were defined. This step produced also a clustering file in the dedicated subfolder Result_files/RAW_CLUSTERING.

What do you need?

This step was developed in the PerlLib::Clustering.pm library (more details inside the code itself) and needs also the Miscellaneaous.pm and ClusteringCFunctions.pm libraries, and also the 'Inline C' library installed.

2.13 HUNTING

```
###HUNTING###
Step to do [yes-no]:        no
Chosen clustering percentage of dissimilarity for the hunting step (%):        5.0
//
```

This specific step of BIOCOM-PIPE called the HUNTING is dedicated to the analysis of clusters defined after the RAW_CLUSTERING step. More precisely, this step will delete rare OTUs from each sample independently, if the OTU is composed of less than 0,01% of sequences (e.g. single sequence for a sample with 10,000 sequences reads), that can be considered as 'chimeras' sequences. However, this is true only if the OTU is composed of different sequences (e.g. two different sequences for a sample with 20,000 sequences), but not composed of sequences with replicates (e.g. one different sequence of two replicated for a sample with 20,000 sequences). The only input parameter defined by the user is the clustering similarity threshold to, do the analysis.

A summary file is produced at the end of this step in the Summary_files folder to define how many sequences have been deleted for each sample. This step produced also two FASTA files in the dedicated subfolder Result_files/HUNTING, one with deleted sequences and the other with kept sequences.

It is noteworthy that this step is more efficient in association with the next step called the RECOVERING. These two specific steps are dedicated to the deletion of potential 'chimera' sequences.

What do you need?

This step was developed in the Prog_Perl/ Hunt_ssingleton_1.6.pl program (more details inside the code itself) and needs also the POSIX package.

2.14 RECOVERING

```
###RECOVERING###
Step to do [yes-no]:        yes
Organism studied [bacteria, fungi, or algae]:        fungi
Database used for the taxonomic assignment [RDP (bacteria only), or Silva (bacteria fungi or algae)]:        Silva
Taxonomic level checked [domain, phylum, class, order, family or genus]:        phylum
Confidence estimates threshold [0-100] :        85
Keep Taxonomy file for deleted reads [yes-no]:        yes
Number of cores or processors used for each sample to do the BLAST analysis [1-32]:        1
//
```

This specific step of BIOCOM-PIPE called the RECOVERING is dedicated to the analysis of clusters deleted by the HUNTING step (see details for this specific step XXX). Indeed,

the HUNTING step put away generally many OTUs from each independent sample. However, some organisms can be under-represented in specific samples, but not considered as ‘chimeras”. To avoid the deletion of these ‘rare’ organisms, we added this RECOVERING step. This step is dedicated to the comparison of these sequences to the database of known sequences with a known taxonomy.

To launch this analysis, the user must give as input several parameters:

- The organism studied (bacteria, fungi or algae) to choose the best suited database to analyze the sequences,
- The chosen origin of the database (RDP or SILVA),
- The taxonomic level to define if the sequences is ‘known’ or ‘unknown’ at this taxonomic level (*phylum, class, order, family or genus*). For example, the *phylum* level will be less stringent than the genus level.
- The similarity (or confidence) threshold to consider if the sequence is affiliated or not to known sequences of the chosen database,
- The user can choose to keep or not the taxonomic results of analyzed sequences into a specific file in the subfolder Result_files/RECOVERING.
- The last input parameter is the number of cores or processors for each sample to realize the comparison (using USEARCH or BLAST) if needed.

A summary file is produced at the end of this step in the Summary_files folder to define how many sequences have been ‘recovered’ and reintroduced to each sample. This step produced also a FASTA file in the dedicated subfolder Result_files/RECOVERING, with cleaned sequences.

What do you need?

This step was developed in the Prog_Perl/ Taxo_recovery3.0.pl program (more details inside the code itself) and needs also the package File::Copy. This PERL program needs also the access to several third-party tools, such as the JAVA Multiclassifier.jar (stored in the folder Data/Tools/RDP_Multiclassifier/), usearch (stored in the folder Data/Tools/USEARCH/) and blastn. This program needs also the access to specific databases (Data/Databases/BACTERIA/SILVA/, Data/Databases/ALGAE/SILVA or Data/Databases/FUNGI/SILVA) and PERL programs like Bacteria_SILVA_Taxonomy_X.X.pl, Fungi_SILVA_Taxonomy_X.X.pl and Algae_SILVA_Taxonomy_X.X.pl.

2.15 RANDOM_CLEANED

```
###RANDOM_CLEANED###
Step to do [yes-no]:        no
Number of reads in the subset:        200
//
```

This step was developed to allow the user to homogenize the treated dataset from various sequences files (.fasta, .fna, .sff, or .fastq). More precisely, it takes as input the number of sequences needed for each sample. Based on this input number, the program will select randomly the needed number of sequences for each independent sample. If the initial number of sequences in a sample is below the wanted number of sequences, the program will copy the file without random selection.

A summary file is produced at the end of this step in the Summary_files folder. This file describes for each sample if the random selection was realized or not. This step produced also a FASTA file (and a quality file if needed) for each sample in the dedicated subfolder Result_files/RANDOM_CLEANED.

Note: only one random selection is authorized by the pipeline (this step can be done on samples after demultiplexing, after preprocessing or after all filters), as several subsampling steps can bias the analysis. So, if the user chose several random steps, the main program will terminate the analysis and indicate to the user the problem.

What do you need?

This step was developed in the PerlLib::RandomSelection.pm library (more details inside the code itself), and needs also the Miscellaneaous.pm library and the List::Util 'shuffle'.

2.16 TAXONOMY

```
###TAXONOMY###
Step to do [yes-no]:        yes
Organism studied [bacteria, or fungi]:        bacteria
Database used for the taxonomic assignment [RDP (bacteria only), or Silva (bacteria and fungi)]:        Silva
Confidence estimates threshold [0-100] :        80
Number of cores or processors used for each sample to do the BLAST analysis [1-32]:        1
//
```

This step (TAXONOMY) was developed to allow the user to assign taxonomically the analyzed against a specific database and dedicated tools chosen by the user. Indeed, several curated databases are available in the BIOCOM-PIPE pipeline (such as SILVA or RDP), associated to third-party tools (i.e. the RDP Multiclassifier, USEARCH or BLASTn) to compare efficiently analyzed sequences to these databases. To launch this step of analysis, the use must give as input several parameters:

- The studied organism name (bacteria, fungi or archaea) to choose the best suited database,
- The origin of the used database (RDP for bacteria and archaea, or SILVA bacteria, archaea or fungi),
- The version of the database chosen for SILVA (R114 or R132 for example),
- The similarity (or confidence) threshold to consider if the sequence is affiliated or not to known sequences of the chosen database,
- The last input parameter is the number of cores or processors for each sample to realize the comparison (using USEARCH or BLAST) if needed.

It is noteworthy that the BLASTn approach is used only with the SILVA database (for fungi, version R114), and USEARCH for other databases and/or versions, except the RDP database, where its specific tool is implemented (RDP MultiClassifier).

Several summary files are produced at the end of this step in the Summary_files folder. More precisely, for each taxonomic level (phylum, class, order, family or genus), a specific tabular file will list the number of sequences for each sample with known taxonomy. This step produced also a taxonomy file for each sample in the dedicated subfolder Result_files/TAXONOMY. Finally, some automatic output graphs are produced to visualize the sample distributions (pies, heatmaps, barplots, etc.), also stored in the subfolder Summary_files/ Figures.

What do you need?

This step was developed in the PerlLib:Taxonomy library (more details inside the code itself). This PERL program needs also the access to several third-party tools, such as the JAVA Multiclassifier.jar (stored in the folder Data/Tools/RDP_Multiclassifier/), usearch (stored in the folder Data/Tools/USEARCH/) and blastn. This program needs also the access to specific databases (Data/Databases/BACTERIA/SILVA/, Data/-Databases/ALGAE/SILVA or Data/Databases/FUNGI/SILVA) and PERL programs like Bacteria_SILVA_Taxonomy_X.X.pl, Fungi_SILVA_Taxonomy_X.X.pl and Algae_SILVA_Taxonomy_X.X.pl. Moreover, some other programs are essential, such as the PERL program Prog_Perl/Taxo_treatment_2.6.pl (more details inside the code itself) using data from some sources (Data/Databases/BACTERIA/RDP/Structures/, Data/Databases/BACTERIA/SILVA/Structures/ and Data/Databases/FUNGI/SILVA/Structures/ and Data/Databases/ALGAE/SILVA/Structures). Finally, the graphical outputs are generated using the PYTHON program Prog_Python/Taxo_graph_computations_2.1.py. This program needs also some specific libraries like matplotlib, sys, os, random, math and the folder Library (inside the Prog_Python/Library/ folder).

2.17 CLEAN_INFERNAL_ALIGNMENT

```
###CLEAN_INFERNAL_ALIGNMENT###
Step to do [yes-no]:        no
Covariance model used [bacteria, archaea, algae or fungi]:        bacteria
//
```

This step is dedicated to the definition of a global alignment against dedicated structures of rRNA (16S for bacteria and/or archaea, 18S for fungi, and 23S for plastidial microeukaryotic organisms) using the Infernal alignment program ('INFERence of RNA ALignment', v1.1.1) [Nawrocki and Eddy, 2013]. As input, the user must choose which group of microorganisms is studied, to use the best structure. Infernal was run chosen as a structural model allow for divergent but valid sequences to persist because stricter methods may incorrectly discard some sequences [Lynch and Neufeld, 2015]. Moreover, this alignment provides a more intuitive handling of sequencing errors, such as homopolymer errors that are easily detected thanks to the secondary-structure aware aligner. Therefore, such homopolymer errors can be

easily managed and ignored during the clustering step (see RAW_CLUSTERING or CLEAN_CLUSTERING steps for details).

This step produced a file containing aligned sequences (FASTA or STOCKHOLM format) for each sample in the dedicated subfolder Result_files/ RAW_INFERNAL_ALIGNMENT.

What do you need?

This step was developed in the PerlLib::InfernalAlignment.pm library (more details inside the code itself) and needs also the 'POSIX' library and the third-party-tool Infernal installed (V1.1.1), with direct launching of some commands like 'cmalign' or 'esl-alimerge'. Moreover, this program needs also the structures stored in the Prog_Perl/Matrix/ subfolder, and the Miscellaneaous.pm library.

2.18 CLEAN_CLUSTERING

```
###CLEAN_CLUSTERING###
Step to do [yes-no]:        no
Ignoring homopolymers differences [yes-no]:        yes
Ignoring distances at the beginning of sequences [yes-no]:        yes
Maximum percentage of dissimilarity for clustering (%):        5.0
Step size for each cluster (%):        5.0
Rank-abundance curves computation [yes-no]:        yes
//
```

This step of analysis was developed to cluster analyzed sequences into OTUs using the globally aligned sequences with Infernal [Nawrocki and Eddy, 2013]. This program needs four parameters. The first parameter indicates if homopolymer differences will be considered as true differences or not. Homopolymer stretches can be problematic for several sequencing technologies (e.g. 454, IonTorrent, MinION, etc.). These sequencing platforms suffer from the inaccuracy in detecting the length of homopolymers repeats of the same nucleotide, so our program, using the global alignment, can detect such stretches (see example 2.1).

Here, two sequences are compared by the clustering program after global alignment with Infernal. As Infernal is based also on the secondary structure of the rRNA, it can easily detect insertion-deletion errors originated from the sequencing step. As one of the two sequences has a high occurrence (here, 43 occurrences, indicated in its ID), the difference is ignored by the clustering program. Indeed, the sequence with 43 occurrences (after strict dereplication) can be considered as a "true" sequence. The difference with the other sequence (with only one occurrence) will be ignored during the clustering process. However, such differences cannot be detected on singleton sequences (if the two sequences have only one occurrence), as we are unable

to confirm if there is a 'true biological' difference or not. Ignoring homopolymer differences can drastically reduce the number of defined OTUs (between 5 to 20%, depending on the origin of the sequencing, and the quality of the sequencing).

The second parameter allow the user to avoid the differences between two sequences harboring length differences. So, the program will consider only common parts of the sequences, and ignore specific parts of the analyzed sequences. The third and fourth parameters defined the maximum percentage of dissimilarity tolerated to create OTUs, and also the step size (if needed) to realize several clustering with different dissimilarity percentages. For example, if the maximum is defined to 5.0% de with a step size of 1.0%, the program will create a clustering with a threshold of 1% of dissimilarity, another with 2.0%, another with 3.0%, another with 4.0% and finally a last with 5.0% of threshold.

First of all, dereplicated sequences will be organized by decreasing abundance, and then they will be clustered based on the threshold chosen by the user. The clustering process is a is a greedy incremental clustering algorithm developed in C (introduced in PERL by the Inline specific module). The first sequence from the dataset becomes the seed of the first OTU (this first seed is also the most abundant sequence, and potentially the truest one). Then, the distance between the seed and each remaining sequence is compared. If the distance of the query to the seed sequence is equal or below a given threshold then it is assigned to that cluster. Otherwise, a new cluster is defined with that sequence as the seed.

A summary file is produced at the end of this step in the Summary_files folder. This file describes for each sample how many OTUs were defined. This step produced also a clustering file in the dedicated subfolder Result_files/RAW_CLUSTERING.

What do you need?

This step was developed in the PerlLib::Clustering.pm library (more details inside the code itself) and needs also the Miscellaneaous.pm and ClusteringCFunctions.pm libraries, and also the 'Inline C' library installed.

2.19 COMPUTATION

```
###COMPUTATION###
Step to do [yes-no]:        no
Chosen clustering step for analysis [Raw, Clean, Both]:        Raw
Chosen percentage of dissimilarity for clustering (%):        5.0
Determination of rarefaction curve(s) [yes-no]:        yes
Determination of rank-abundance curve(s) [yes-no]:        yes
Computation of the full bias corrected Chao1 richness estimator [yes-no]:        yes
Computation of the ACE richness estimator [yes-no]:        yes
Computation of bootstrap estimate, and shannon and simpson indexes [yes-no]:        yes
//
```

The COMPUTATION step of analysis is based on the OTUs definition obtained previously either by the RAW or CLEAN_CLUSTERING. This step computes several indices and graphical outputs to evaluate and describe the microbial community composition. The COMPUTATION step needs several input parameters from the user:

- The chosen clustering to analyze (the RAW_CLUSTERING, the CLEAN_CLUSTERING or both),
- The dissimilarity percentage used to compute the clustering,
- If rarefaction and/or rank-abundance curves are computed and drawn or not (two parameters, one for each type of curves),
- The mathematical computation of classical richness indices (Chao1 for example),
- The mathematical computation of other richness indices such as ACE,
- The mathematical computation of other indices (bootstrap, Shannon, Simpson, evenness).

Several summary files are produced at the end of this step in the Summary_files folder with indices computed (Indices.txt) but also if needed the descriptive curves in PDF in the subfolder Curves_data.

What do you need?

This step was developed in the PerlLib::ProgramLaunchers::ComputationLauncher.pm library (more details inside the code itself). This program will use some PYTHON programs (Computation_rc_estimators_3.1.py and Curves_drawing_1.7.py from the folder Prog_Python), and need also some external libraries (sys, random, math, decimal, gmpy, os, matplotlib and Library (Prog_Python/Library/).

2.20 GLOBAL_ANALYSIS

```
###GLOBAL_ANALYSIS###
Step to do [yes-no]:        no
Chosen clustering step for analysis [Raw, Clean, Both]:        Raw
Chosen percentage of dissimilarity for the treated clustering (%):        5.0
Defining the taxonomic assignment of all OTUs in the global matrix [yes-no]:        yes
Confidence estimates (RDP) or similarity percentage (SILVA) threshold [0-100] :        80
Realization of a phylogenetic tree and an ID mapping file for UNIFRAC analysis (time-consuming step !!) [yes-no]:        no
Selection of the most abundant read to represent each OTU for UNIFRAC analysis (tree and mapping file) [yes-no]:        no
//
```

The GLOBAL_ANALYSIS step was computed to define a global matrix of OTUs regarding the complete dataset of samples analyzed. To do this, the GLOBAL_ANALYSIS needs results obtained previously either by the RAW or CLEAN_INFERNAL_ALIGNMENT step, the RAW or CLEAN_CLUSTERING step and potentially the TAXONOMY step. Several important analyses can be realized by the GLOBAL_ANALYSIS, such as the definition of a global matrix of OTUs for all analyzed samples, the taxonomical assignment of these OTUs, the computation of a global phylogenetic tree (using aligned sequences from the INFERNAL_ALIGNMENT step and FastTree) and an ID mapping file, essential for the UNIFRAC_ANALYSIS step.

The GLOBAL_ANALYSIS step needs several input parameters from the user:

- The chosen clustering to analyze (the RAW_CLUSTERING, the CLEAN_CLUSTERING or both), that will help the BIOCOM-PIPE to check if all previous steps have been computed or not,
- The dissimilarity percentage used to compute the clustering,
- If the taxonomic assignment of OTU sis realized or not. If the user chose to compute this taxonomy, the TAXONOMY step will be essential, and the BIOCOM-PIPE will check if this step was computed or not,
- The similarity (or confidence) threshold to consider if the sequence is affiliated or not to known sequences of the chosen database,
- If the definition of a phylogenetic tree (using aligned sequences from the INFERNAL_ALIGNMENT step and the third-party tool FastTree) in Newick format and an associated ID mapping file was done or not,
- And, if this phylogenetic tree is defined using all sequences from the analysis, or if only one representative sequence is used for each OTU of the global matrix.

Several summary files are produced at the end of this step in the Summary_files folder, all of them stored in the Global_analysis subfolder. First, two global OTUs matrices are computed, one with samples as lines, and the other with samples as columns, and potentially (if the user requested this analysis) the file describing the taxonomic assignment of all OTUs. The two files (phylogenetic tree and ID

mapping file, called respectively the Clean_Tree_Concatenated_reads.fasta and the Clean_ID_mapping.file.txt) essential for the UNIFRAC_ANALYSIS are stored in a subfolder called Unifrac_analysis in the Global_analysis folder.

What do you need?

This step was developed in the Perl-Lib::ProgramLaunchers::GlobAnalysisLauncher.pm library (more details inside the code itself) that launches the Globanalysis_matrix_computation_3.0.pl from the Prog_Perl folder. This PERL program calls also the Globanalysis_tree_computation_2.0.pl PERL program (that needs the third-party tool FastTree). This step requires several other libraries (ManagingDirectories::FindDirectory, PoolingReads::PoolingReads, Dereplication::Dereplication, GlobalAlignment::GlobalAlignment, Clustering::Clustering) and their corresponding dependencies (more details inside the code itself).

2.21 RECLUSTOR

```
###RECLUSTOR###
Step to do [yes-no]: yes
Do you want to define a new database to realize the clustering step [yes-no]: no
Do you want to use an existing database to realize the clustering step [yes-no]: yes
Do you want to enrich the used database with treated read [yes-no]: no
Database name (short name without spaces or special characters): RMQS
Covariance model used [bacteria, archaea, algae or fungi]: bacteria
Chosen percentage of dissimilarity for the clustering (default 5%) [1-100]: 5.0
Ignoring homopolymers differences during the clustering [yes-no]: yes
Ignoring differences at the beginning of sequences [yes-no]: yes
//
```

The RECLUSTOR step is a new post-clustering tool (for RE-CLUSTering method using an open-reference approach) to improve OTU consistency [Terrat *et al.*, 2020]. This new strategy combines two previously described clustering methods. Firstly, a classical clustering method is used to define OTU centroids and create a reference database. Secondly, a closed- or open-reference method (depending on the user's choice) is computed for all sequences which are not considered as OTU centroids. Each sequence is then compared to all centroids using a distance-based greedy clustering method, and assigned to the nearest one, thereby fixing the erroneous assignments of sequences to OTUs (Figure 2.2). For sequences that did not match with any reference sequence (i.e. 'out-sequences'), these ones can be clustered using the de novo method to define new OTUs.

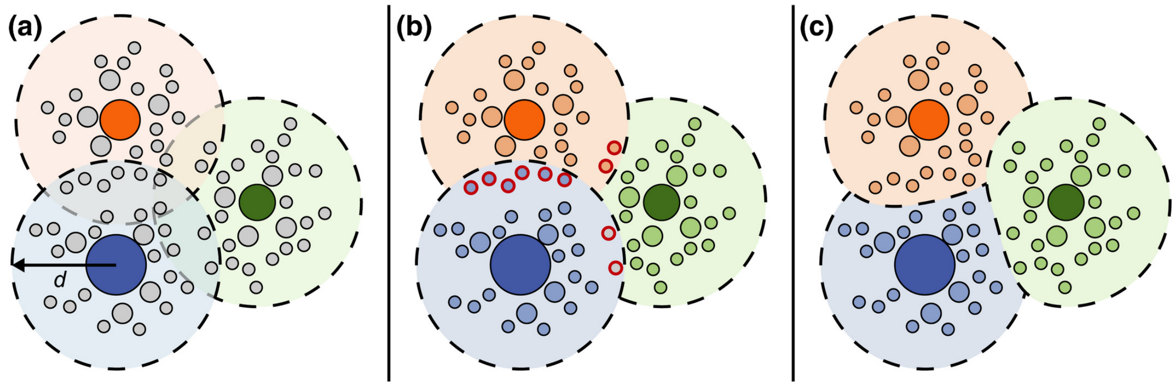

Fig. 2.2 – **Schematic view of classical greedy clustering and ReClustOR approaches.** Reads were represented by closed circles, and their abundance by the circle sizes. The clustering threshold distance (d) was chosen by the user. (a) Three reads were defined as Operational Taxonomic Units (OTUs) seeds (represented in blue, orange and green), with a centroid definition based on abundance. As these three reads were close together, related reads can be placed in different OTUs. (b) With a greedy clustering approach, the OTU formed from the blue read will regroup several reads originated from other OTUs, leading to inaccurately formed OTUs. (c) With ReClustOR, the OTUs seeds were kept as they are more divergent than the chosen distance (d). However, each read was compared to all centroids, and assigned to the closest one, thereby fixing erroneous assignments of reads to OTUs

As the GLOBAL_ANALYSIS, the RECLUSTOR step needs results obtained previously by the GLOBAL_ANALYSIS, and as a consequence, results from either by the RAW or CLEAN_INFERNAL_ALIGNMENT step, the RAW or CLEAN_CLUSTERING step and potentially the TAXONOMY step. The RECLUSTOR allows the user either (i) to define a new database using the analyzed dataset, and then improve the clustering of the samples and their corresponding sequences, or (ii) to compare the analyzed samples to an already defined database to realize the clustering step (enriching or not the database with new OTUs).

The RECLUSTOR step needs several input parameters from the user:

- If the user wants to define a new database of OTU centroids using the results obtained from the GLOBAL_ANALYSIS,
- If the user wants to compare the analyzed samples to an existing database, to define its OTUs,
- If the user wants to enrich the database with OTUs defined by the de novo method that did not match any reference sequence (i.e. ‘out-sequences’), or not,
- The database name chosen by the user (either to define a new database, or to compare the samples to an existing one),

- The user must choose which group of microorganisms is studied, to use the best structure for the global alignment,
- The dissimilarity threshold used for the clustering steps (database comparison and if needed the de novo method for 'out-sequences'),
- If homopolymer differences will be considered as true differences or not (depending on the sequences origin),
- The final parameter allows the user to avoid the differences between two sequences harboring length differences, if needed.

Several summary files are produced at the end of this step in a specific folder called RECLUSTOR into the Summary_files folder. More precisely, two files are produced: (i) one describing the results obtained after the RECLUSTOR step (called the Clustering_summary_XX.txt file), with the number of OTUs obtained against the database, the number of OTUs from the de novo step, and the corresponding number of sequences associated for each clustering; (ii) and the second is the global OTUs matrix after RECLUSTOR (called Reclustered_OTU_matrix_XX.txt). This step produced also a clustering file (called Clustering_results_XXX.txt) in the dedicated subfolder Result_files/RECLUSTOR describing for each sequence the highest result obtained (against the database or with the de novo step if needed).

What do you need?

This step was developed in the PerlLib::ReClustor.pm library (more details inside the code itself). This program will use some external programs (such as the esl-alimerge of the cmalign INFERNAL programs), some PERL libraries (Miscellaneous.pm, InfernalAlignment.pm, orClustering.pm) and their corresponding dependencies.

2.22 UNIFRAC_ANALYSIS

```
###UNIFRAC_ANALYSIS###
Step to do [yes-no]:        no
Calculate a UniFrac Distance Matrix and apply PCoA and UPGMA [yes-no]:        yes
Compute the Phylogenetic Diversity (PD) of all samples [yes-no]:        yes
Use abundance weigths (select whether the abundance of reads will be used or not for UniFrac tests) [yes-no]:        yes
//
```

The last step available in BIOCOM-PIPE is the UNIFRAC_ANALYSIS. It relies on a Python program developed by Lozupone et Knight, available in their website. This step of analysis is dedicated to the computation of a UniFrac distance matrix between samples, but also Phylogenetic Diversity indices (called also PDs), a dendrogram between samples and a PCoA and a UPGMA with this distance matrix, and finally some statistical tests to check the reliability of the obtained results (and more specifically

on the phylogenetic tree used). This module needs a phylogenetic tree computed by previous steps of Newick format with the third-party tool FastTree, and consequently the use of this previous steps.

The UNIFRAC_ANALYSIS step needs several input parameters from the user:

- If the program needs or not to compute a UniFrac distance matrix between samples, and a PCoA and a UPGMA with this distance matrix,
- If the program needs or not to compute Phylogenetic Diversity (PD) indices,
- If the program considers the abundance of each OTU of the complete dataset, or consider only their presence/absence (weighted matrix or not).

Several summary files are produced at the end of this step in a specific folder called Global_analysis into the Summary_files folder. For example, it will produce files like:

- Phylogenetic_Diversity_distance.txt, with Phylogenetic Diversity (PD) indices,
- Unifrac_Cluster_samples.tre, a dendrogram between samples, based on the UniFrac distance matrix,
- Unifrac_distance_matrix.txt, the UniFrac distance matrix itself,
- Unifrac_PCoA.txt, the PCoA data from the analysis of the distance matrxi computed with UniFrac (axes, weight of these axes, etc...).

What do you need?

This step was developed in the Prog_Python::Unifrac_computation_1.1.py PYTHON program (more details inside the code itself). This program will use some external libraries (sys, types, os, random, math, matplotlib, cogent et Library(Data/Library)).

Installation 3

3.1 Quick installation instructions

3.1.1 System requirements

BIOCOM-PIPE is designed to run on POSIX-compatible platforms, including UNIX, Linux and MacOS/X. We have tested most extensively on Linux because these are the machines we develop on.

3.1.2 INSTALLATION OF BIOCOM-PIPE

After downloading the **BIOCOM-PIPE_vX.X** software (available at this https://zenodo.org/record/1123425), first of all, copy the data from the archive BIOCOM-PIPE_vX.X.tar.gz to a new folder (e.g. /usr/local/BIOCOM-PIPE_vX.X/) or extract and move directly the archive to /usr/local/BIOCOM-PIPE_vX.X/ for example (recommended).

Add BIOCOM-PIPE_vX.X/ folder to system PATH, this will enable you to run BIOCOM-PIPE software and databases from within any folder on your computer. For Ubuntu users, you can do so by editing the /etc/environment file or for Debian users, you can do so by editing the /etc/profile file. Open the file in edit mode and enter the following at the end (replace path_to_BIOCOM-PIPE_vX.X_folder with the real path).

```
user@biocomPipe:~$> PATH=~/path_to_BIOCOM-PIPE_vX.X_folder:${PATH}
```

It is recommended that this step be made for all users.

To install the third-party tools, it is necessary to have permissions. Open a terminal window and enter :

```
user@biocomPipe:~$> sudo chmod -R ugo+rwx path_to_BIOCOM-PIPE_vX.X/
```

3.1.3 INSTALLATION OF THIRD-PARTY TOOLS

Nota bene: The open-source third-party tools are available into BIOCOM-PIPE_vX.X/Data/ThirdPartyTools/ folder.

3.1.3.1 GCC COMPILER AND MAKE

A GCC compiler is necessary, it is recommend to install one, for example « gcc »:

```
user@biocomPipe:~$> sudo apt-get install gcc
```

Make tool is necessary, it is recommend to install one, for example « make »:

```
user@biocomPipe:~$> sudo apt-get install make
```

3.1.3.2 INFERNAL (v1.1.1)

This tool is available and can be downloaded from the following address click here on the website http://eddylab.org/infernal/ but is also given directly in BIOCOMPIPE_vX.X/Data/ThirdPartyTools/INFERNAL/ folder.

After retrieve and extract the archive infernal-1.1.1.tar.gz, move to infernal-1.1.1/easel/ folder which contains many sub-applications required.

```
user@biocomPipe:~$> tar -zxvf infernal-1.1.1.tar.gz
user@biocomPipe:~$> cd infernal-1.1.1/easel/
```

Once there, follow the software instructions (read the INSTALL file). It is recommended to use the root mode with sudo for the following commands:

```
user@biocomPipe:~$> ./configure
user@biocomPipe:~$> make
user@biocomPipe:~$> make check
user@biocomPipe:~$> make install
```

This will install the small tools needed for the Infernal tool.

Next step, go back to infernal-1.1.1/ folder and proceed as above to install the main tools (always with root privileges).

```
user@biocomPipe:~$> cd ..
user@biocomPipe:~$> ./configure
user@biocomPipe:~$> make
user@biocomPipe:~$> make check
user@biocomPipe:~$> make install
```

To check if it has been correctly installed, enter to the terminal console:

```
user@biocomPipe:~$> cd
user@biocomPipe:~$> esl-alimerge -h
user@biocomPipe:~$> cmalign -h
```

If these commands return the help, it is works if not that means there seems to be a problem during the installation. The infernal-1.1.1/ folder initially created is no longer necessary, and can be deleted if you wish.

```
user@biocomPipe:~$> rm -R infernal-1.1.1/
```

3.1.3.3 FastTree (v2.1.10)

The binary of this tool is available and can be downloaded from the following address click here but is also given directly in BIOCOM-PIPE_vX.X/Data/ThirdPartyTools/FastTree/ folder. However, it is recommended to build the FastTree.c file (available at this url click here) with your distribution in order to be adjust to your environment. Move the FastTree.c file to FastTree/ folder.

```
user@biocomPipe:~$>
    mv FastTree.c BIOCOM-PIPE_vX.X/Data/Tools/FastTree/
```

It is recommended to use the root mode with sudo for the following commands:

```
user@biocomPipe:~$> cd BIOCOM-PIPE_vX.X/Data/Tools/FastTree/
user@biocomPipe:~$> gcc -DOPENMP -fopenmp -O3 -finline-functions
    -funroll-loops -Wall -o FastTreeMP-2.1.10 FastTree.c -lm
```

Special care, the output file should be called FastTreeMP-2.1.10, if not, BIOCOM-PIPE will not be able to retrieve it. The FastTree.C file can be deleted if you wish. The binary must be in the FastTree/ folder.

3.1.3.4 PrinSeq (v0.20.4)

This PERL tool is available and can be downloaded from the following address click here but is also given directly in BIOCOM-PIPE_vX.X/Data/ThirdPartyTools/PRINSEQ/ folder. Extrat the archive, then copy the prinseq-lite.pl file from prinseq-lite-0.20.4/ and paste into PRINSEQ/ folder.

```
user@biocomPipe:~$> tar -zxvf prinseq-lite-0.20.4.tar.gz
user@biocomPipe:~$> cp prinseq-lite-0.20.4/prinseq-lite.pl
    BIOCOM-PIPE_vX.X/Data/Tools/PRINSEQ/
```

The prinseq-lite-0.20.4/ folder can be deleted if you wish because only the prinseq-lite.pl is necessary.

3.1.3.5 FLASH (v1.2.11)

The source package of this tool is available and can be downloaded from the following address: click here but is also given directly in BIOCOM-PIPE_vX.X/Data/ThirdPartyTools/FLASH/ folder. Attention was paid to the fact that several external librairies are required, in particular zlib1g-dev which must be install before. Extract the archive, then move to the FLASH-1.2.11/ folder newly created and it is recommended to use the root mode with sudo for the following commands:

```
user@biocomPipe:~$> tar -zxvf  FLASH-1.2.11.tar.gz
user@biocomPipe:~$> cd FLASH-1.2.11/
user@biocomPipe:~$> make
```

3.1.3.6 RDP MULICLASSIFIER (v1.0)

This tool is available and can be downloaded from the following address: click here but is also given directly in BIOCOM-PIPE_vX.X/Data/ThirdPartyTools/RDP_Multiclassifier/ folder. This JAVA tool required Java Runtime Environment. Therefore we need to install JRE

```
user@biocomPipe:~$> sudo apt-get install default-jre
```

Then move MultiClassifier.jar file into RDP_Multiclassifier/ folder.

```
user@biocomPipe:~$> mv
    BIOCOM-PIPE_vX.X/Data/ThirdPartyTools/RDP_Multiclassifier/MultiCla
     BIOCOM-PIPE_vX.X/Data/Tools/RDP_Multiclassifier/
```

3.1.3.7 USEARCH (v6.0.307 or higher)

This tool is available and can be downloaded from the following address click here. There are two different versions of this program, one free Usearch (32-bit) and one paying Usearch (64-bit) (click here). The paying version is more interesting but too prohibitive. The free version is completely usable. In any case, the binary must be move to BIOCOM-PIPE_vX.X/Data/Tools/USEARCH/ folder and must be called « usearch ».

3.1.3.8 BLAST (ncbi-blast+ v2.6.0-1 or higher)

In BIOCOM-PIPE, this tool is used to compare sequences with FUNGI database. To install this tool, it is recommended to use the root mode with sudo for the following commands:

```
user@biocomPipe:~$> sudo apt-get install ncbi-blast+
```

3.1.3.9 PERL (v5 or higher) and et related modules

Most programs developed into BIOCOM-PIPE are written in PERL language. It is therefore necessary to have PERL installed and various modules. To know the modules that are already installed, open a terminal window and enter :

```
user@biocomPipe:~$> cpan -l
```

Here is a list of software to be install:

- File::ShareDir (v1.104 or higher)
- File::ShareDir::Install (v0.11 or higher)
- Inline:: (v0.80 or higher)
- Inline::C (v0.78 or higher)
- Math::Int64 (v0.54 or higher)
- Module::CAPIMaker (v0.91 or higher)
- Parse::RecDescent (v1.967015 or higher)
- Pegex (v0.64 or higher)
- Text::Template (v1.46 or higher)

These modules can be download directly from the CPAN website (click here) and manually installed or can be installed via CPAN interface from terminal window:

```
user@biocomPipe:~$> sudo cpan
user@biocomPipe:~$> cpan [1]> install (module name)
```

It is very strongly recommended to install the modules by the CPAN interface for software dependencies more easily recoverable reasons.

3.1.3.10 Python (v2.7) and et related modules

A number of script for BIOCOM-PIPE have been developed in PYTHON language. It is therefore necessary to have PYTHON installed and various modules.

```
user@biocomPipe:~$> sudo apt-get install python
```

Several packages are required:

```
user@biocomPipe:~$> sudo apt-get install python-dev.
user@biocomPipe:~$> sudo apt-get install pkg-config
user@biocomPipe:~$> sudo apt-get install python-pip
user@biocomPipe:~$> sudo apt-get install libfreetype6-dev
user@biocomPipe:~$> sudo apt-get install libgmp3-dev
```

To finish, several external librairies are required:

- matplotlib (v1.4.3)
- gmpy (v1.17 or higher)
- numpy
- cogent (v1.9 or higher)
- configparser

To proceed to the installation of these external librairies, it is recommended to use the « pip » command:

```
user@biocomPipe:~$> sudo pip install gmpy
user@biocomPipe:~$> sudo pip install numpy
user@biocomPipe:~$> sudo pip install cogent
user@biocomPipe:~$> sudo pip install configparser
user@biocomPipe:~$> sudo pip install 'matplotlib==1.4.3'
```

Be careful: It is very important that matplotlib must be install with the 1.4.3 version to be correctly run into BIOCOM-PIPE.

The BIOCOM-PIPE_vX.X contains many PERL, Python and C scripts. In order for all these scripts to be executed by any user, the execution rights must be modified within the BIOCOM-PIPE_vX.X folder. Open a terminal window and enter :

```
user@biocomPipe:~$> sudo chmod -R ugo+rwx path_to_BIOCOM-PIPE_vX.X/
```

Tutorial

4

4.1 Dataset

To perform this tutorial, we used a dataset of fungal amplicons (18S rRNA) from soil samples and accessible with the accession number PRJEB14258 and refers to the publication [Sadet-Bourgeteau *et al.*, 2018].

4.2 Creating a pipeline analysis

4.2.1 Preparation files

You must create in your analysis folder (example_analysis):

1. a **Raw_data** folder in order to put your sequence files.

2. a **Project_files** folder to put your **project.csv file** (see figure 4.1) containing the essential information (and a follow-up of your project) for the creation of the Input.txt file (contains the default pipeline steps).

	A	B	C	D	E	F	G	H	I	J	K
1	PROJECT	LIBRARY_NAME	LIBRARY_NAME_RECEIVED	SAMPLE_NAME	MID_F	MID_R	TARGET	PRIMER_F	PRIMER_R	SEQUENCE_PRIMER_F	SEQUENCE_PRIMER_R
2	biocomPipeTuto	HZH-4	HZH-4	Colmar2012_B1T105	MID057	MID057	18S	FR1	FF390	CGATAACGAACGAGACCT	ANCCATTCAATCGGTANT
3	biocomPipeTuto	HZH-4	HZH-4	Colmar2012_B2T102	MID092	MID092	18S	FR1	FF390	CGATAACGAACGAGACCT	ANCCATTCAATCGGTANT
4	biocomPipeTuto	HZH-4	HZH-4	Colmar2012_B2T103	MID129	MID129	18S	FR1	FF390	CGATAACGAACGAGACCT	ANCCATTCAATCGGTANT
5	biocomPipeTuto	HZH-4	HZH-4	Colmar2012_B4T103	MID007	MID007	18S	FR1	FF390	CGATAACGAACGAGACCT	ANCCATTCAATCGGTANT
6	biocomPipeTuto	HZH-4	HZH-4	Colmar2012_B4T106	MID056	MID056	18S	FR1	FF390	CGATAACGAACGAGACCT	ANCCATTCAATCGGTANT
7	biocomPipeTuto	HZH-4	HZH-4	Colmar2012_SN1T4	MID091	MID091	18S	FR1	FF390	CGATAACGAACGAGACCT	ANCCATTCAATCGGTANT
8	biocomPipeTuto	HZH-4	HZH-4	Colmar2012_SN1T6	MID132	MID132	18S	FR1	FF390	CGATAACGAACGAGACCT	ANCCATTCAATCGGTANT
9	biocomPipeTuto	HZH-4	HZH-4	Feuch2012_106	MID161	MID161	18S	FR1	FF390	CGATAACGAACGAGACCT	ANCCATTCAATCGGTANT
10	biocomPipeTuto	HZH-4	HZH-4	Feuch2012_107	MID021	MID021	18S	FR1	FF390	CGATAACGAACGAGACCT	ANCCATTCAATCGGTANT
11	biocomPipeTuto	HZH-4	HZH-4	Feuch2012_109	MID058	MID058	18S	FR1	FF390	CGATAACGAACGAGACCT	ANCCATTCAATCGGTANT
12	biocomPipeTuto	HZH-4	HZH-4	Feuch2012_110	MID035	MID035	18S	FR1	FF390	CGATAACGAACGAGACCT	ANCCATTCAATCGGTANT
13	biocomPipeTuto	HZH-4	HZH-4	Feuch2012_206	MID055	MID055	18S	FR1	FF390	CGATAACGAACGAGACCT	ANCCATTCAATCGGTANT
14	biocomPipeTuto	HZH-4	HZH-4	Feuch2012_209	MID093	MID093	18S	FR1	FF390	CGATAACGAACGAGACCT	ANCCATTCAATCGGTANT
15	biocomPipeTuto	HZH-4	HZH-4	Feuch2012_409	MID010	MID010	18S	FR1	FF390	CGATAACGAACGAGACCT	ANCCATTCAATCGGTANT
16											

Fig. 4.1 – Example of a project file
It must be named project.csv and all the columns must be completed for all samples

Here are the command lines to perform.

```
user@biocomPipe:~$> mkdir example_analysis
user@biocomPipe:~$> cd example_analysis
user@biocomPipe:~$> mkdir Raw_data Project_files
user@biocomPipe:~$> BIOCOM-PIPE-v1.19 --prep
```

This step consists of:

- Delete all files, subdirectories and all contents from Project_files/. If the project.csv file does not exist, a template file is created and must be completed. If the project.csv exits, the file is kept.

- Check if the raw data in Raw_data/ folder are decrompressed; otherwise it does.

- Create the Input.txt file from the project.csv file in the root of folder pipeline.

If you open the **Input.txt** file you can see all steps by default to run your analysis (see figure 4.2).

```
###PRINSEQ###
Step to do [yes-no]:    yes
Lowest quality score tolerated for the trimming from the 3'-end of the read [0-40]:     30
Lowest quality score tolerated for the trimming from the 5'-end of the read [0-40]:     30
Minimum Length threshold tolerated to keep reads (default: 30): 30
Number of ambiguities (N's) tolerated (default: 1
Sliding window size used to calculate quality score [1-7]:      7
Step size used to move the sliding window [1-10]:       1
//
###FLASH###
Step to do [yes-no]:    yes
Minimum overlap length between two reads (default: 10) [10-100]:        10
Maximum overlap length between two reads (default: 65) [10-100]:        65
Maximum allowed ratio between the number of mismatched base pairs and the overlap length (default: 0.04) [0-100]:       0.04
//
###EVAL_QUAL###
Step to do [yes-no]:    yes
Length: 300
//
###PREPROCESS_MIDS###
Step to do [yes-no]:    yes
Filename/MID_F/MID_R/Sample:    HZH-4/MID057/MID057/Colmar2012_B1T105
Filename/MID_F/MID_R/Sample:    HZH-4/MID092/MID092/Colmar2012_B2T102
Filename/MID_F/MID_R/Sample:    HZH-4/MID129/MID129/Colmar2012_B2T103
Filename/MID_F/MID_R/Sample:    HZH-4/MID007/MID007/Colmar2012_B4T103
Filename/MID_F/MID_R/Sample:    HZH-4/MID056/MID056/Colmar2012_B4T106
Filename/MID_F/MID_R/Sample:    HZH-4/MID091/MID091/Colmar2012_SN1T4
Filename/MID_F/MID_R/Sample:    HZH-4/MID132/MID132/Colmar2012_SN1T6
Filename/MID_F/MID_R/Sample:    HZH-4/MID161/MID161/Feuch2012_106
Filename/MID_F/MID_R/Sample:    HZH-4/MID021/MID021/Feuch2012_107
Filename/MID_F/MID_R/Sample:    HZH-4/MID058/MID058/Feuch2012_109
Filename/MID_F/MID_R/Sample:    HZH-4/MID035/MID035/Feuch2012_110
Filename/MID_F/MID_R/Sample:    HZH-4/MID055/MID055/Feuch2012_206
Filename/MID_F/MID_R/Sample:    HZH-4/MID093/MID093/Feuch2012_209
Filename/MID_F/MID_R/Sample:    HZH-4/MID010/MID010/Feuch2012_409
//
```

Fig. 4.2 – Example of a Input.txt generated

4.2.2 Launch analysis

To start the analysis corresponds to the different steps present in the Input.txt file, just launch the following command. It is possible to specify the number of cores to be used for the steos designed to be parallelized.

```
user@biocomPipe:~$> nohup BIOCOM-PIPE-v1.19 --launch --cores 10 &
```

It is advisable to launch this step in **nohup** allowing to launch the pipeline which will remain active even after the logout of the user who initiated it.

4.2.3 Create figures

This step allows you to generate a local website to synthesize the results of the main steps with interactive tables and graphs from the raw files in the **Summary_files** folder. It also allows you to compress all the files in the **Result_files** folder in order to save on reducing the size of the files.

To do this just run the following command

```
user@biocomPipe:~$> BIOCOM-PIPE-v1.19 --graphs
```

Some screenshots to illustrate the website.

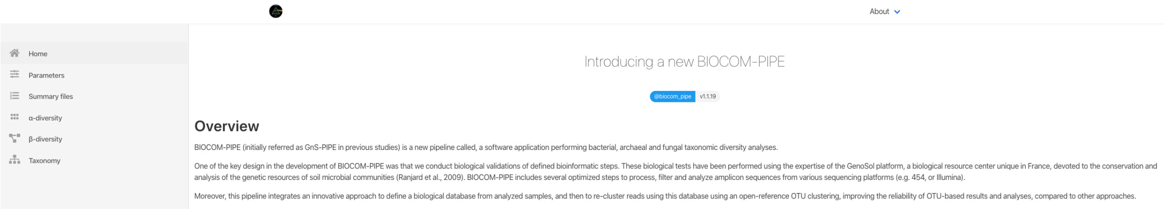

Fig. 4.3 – Local website - Home page

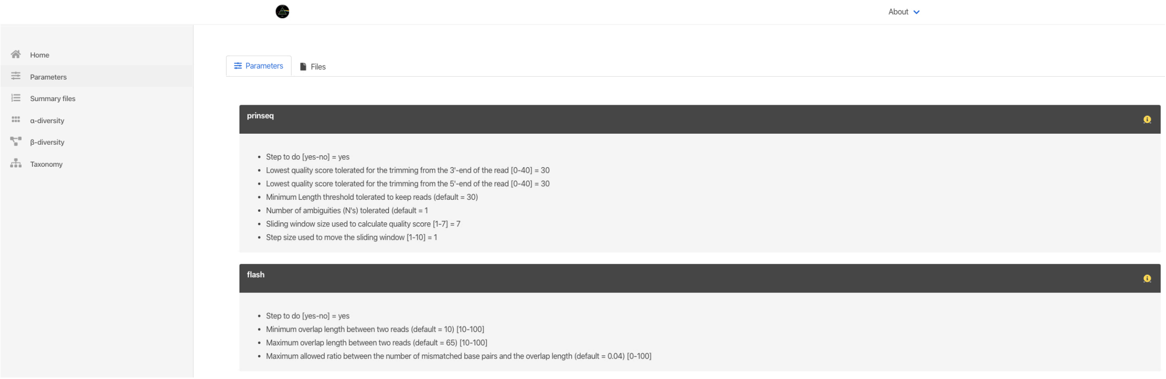

Fig. 4.4 – Local website - Parameters page

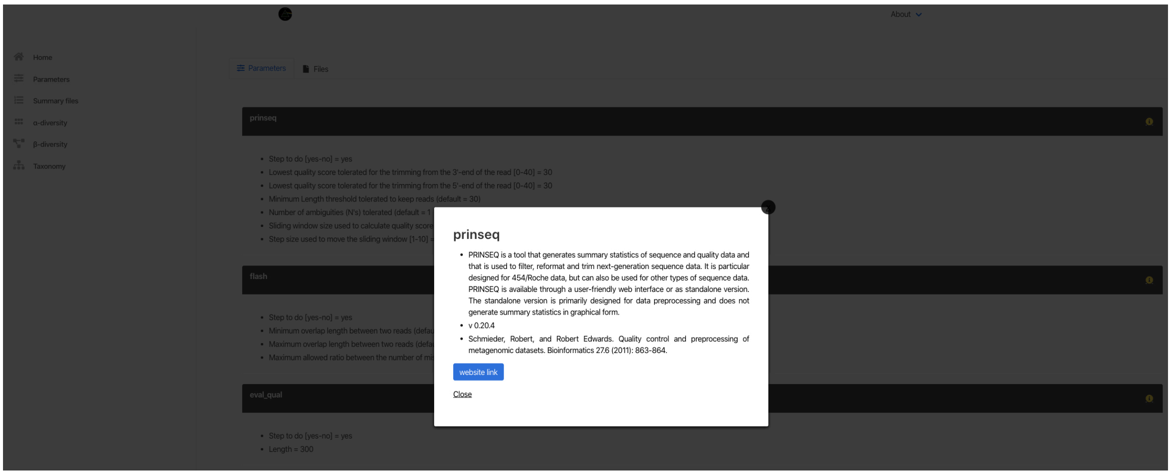

Fig. 4.5 – Local website - Information for Prinseq tool

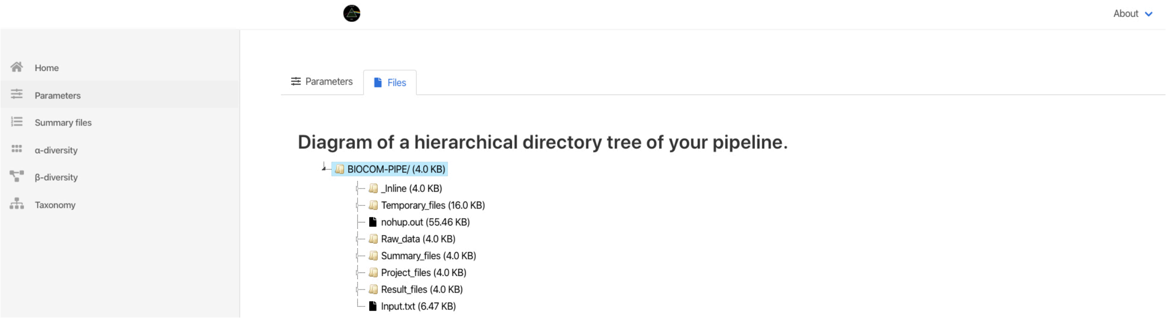

Fig. 4.6 – Local website - Diagram of a hierarchical directory tree of your pipeline.

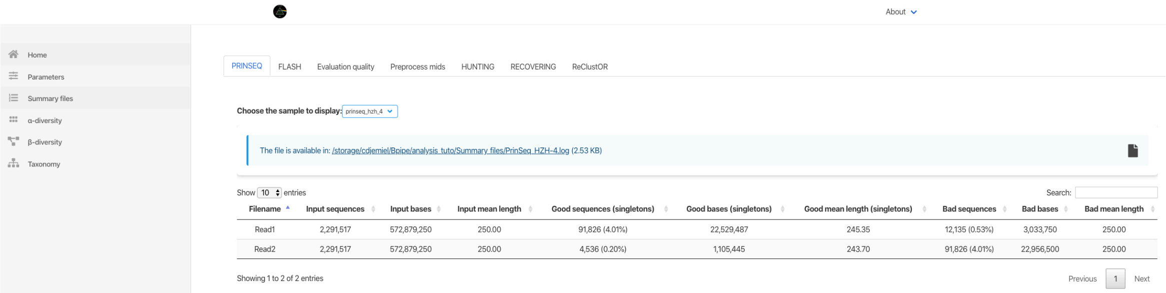

Fig. 4.7 – Local website - Summary files page

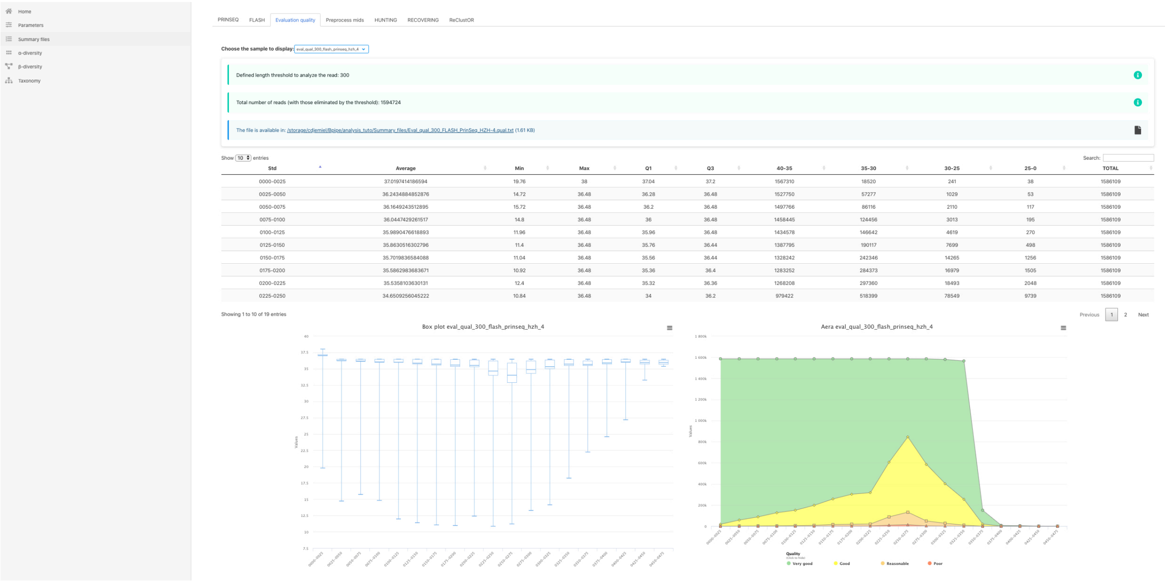

Fig. 4.8 – **Local website - Evaluation of quality from Summary files page**

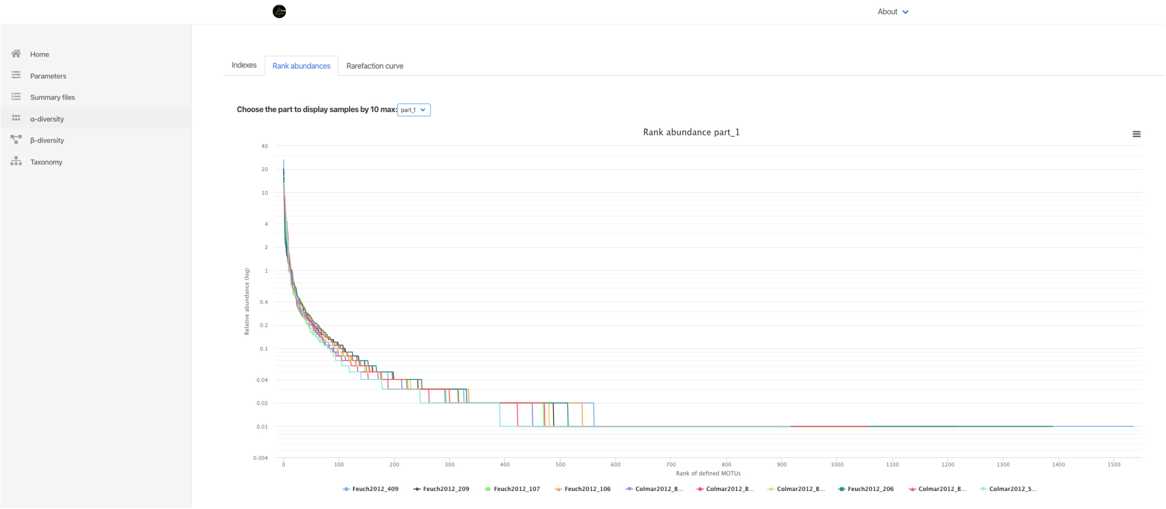

Fig. 4.9 – **Local website - Rank abundance graphs from alpha-diversity page**

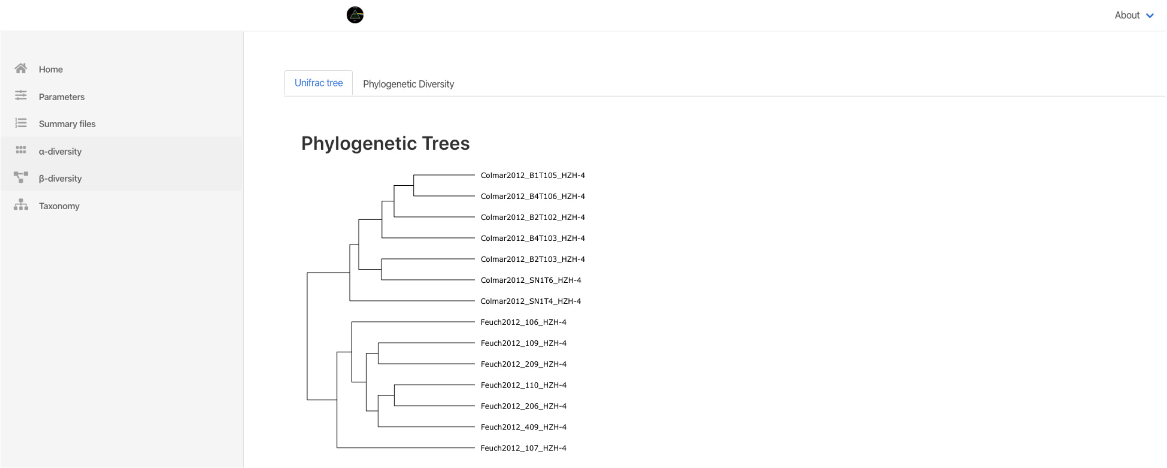

Fig. 4.10 – **Local website - Unifrac tree graphs from beta-diversity page**

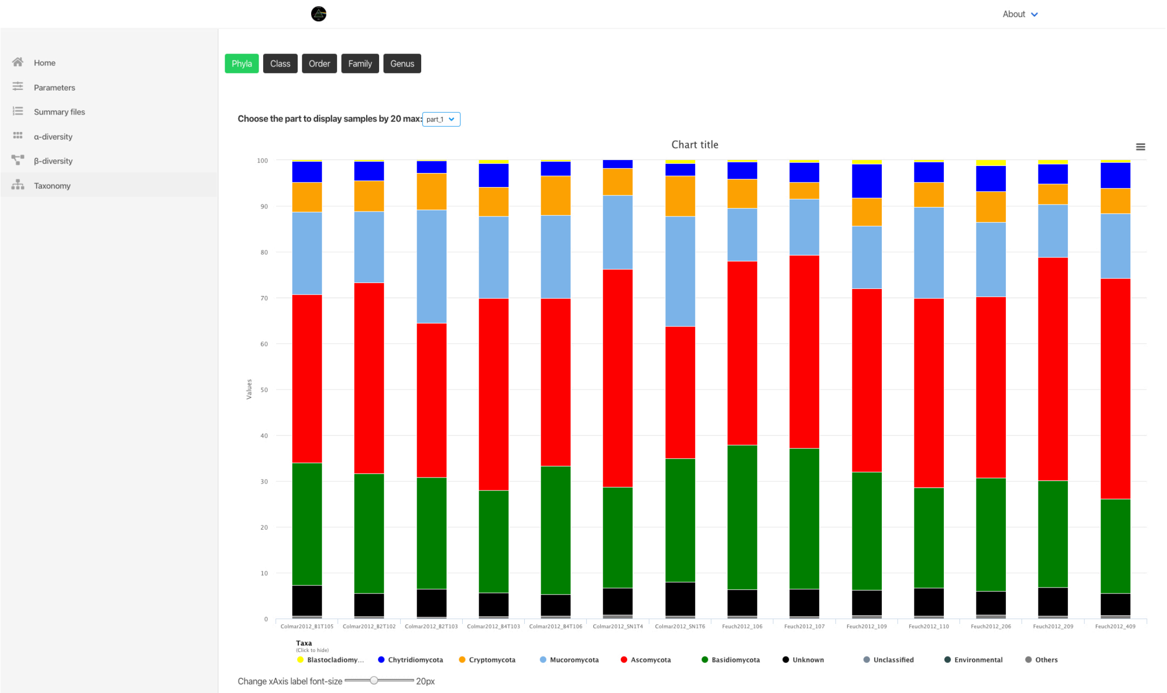

Fig. 4.11 – **Local website - Taxonomy page**
Click on a taxonomic rank to display the relative abundance of samples.

4.3 How we can use the recent/ custom database ?

4.3.1 Databases organization

All databases currently available for BIOCOM-PIPE are stored in the Data/Databases/ folder, by organisms, database origin and versions, into specific sub-folders (see the 4.12).

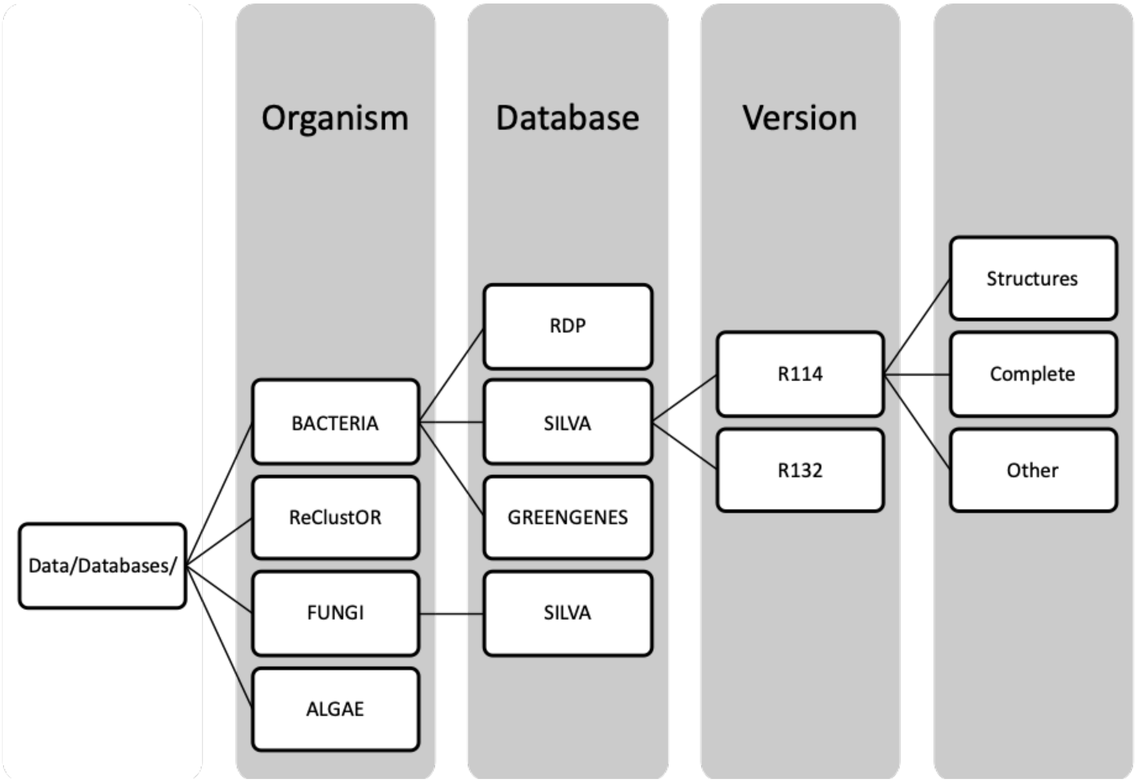

Fig. 4.12 – Databases organization

Into each "Version" folder, three folders can be found. The Stuctures folder contains, for each taxonomic level (phylum, class, order, family, genus) tabulated files describing, for each group found (e.g. for each order) its complete lineage (separated by tabulations). For example, for the Sulfolobales order:

```
Archaea Crenarchaeota Thermoprotei Sulfolobales
```

These files will be used during the treatment of taxonomic results, to obtain a result file identical for all analyses, whatever the organism detected. This can be helpful when comparing several datasets independently analyzed.

The two other folders store the sequence files, after formatting (by BLAST or USEARCH). The FASTA files have to be formatted specifically, to be read by the USEARCH or BLAST programs.

Example:

```
>CJX396637.1.948_Eukaryota(superkingdom);Opisthokonta(no_rank);Nucletmycea
(no_rank);Fungi(kingdom);Dikarya(subkingdom);Basidiomycota(phylum);
Agaricomycotina(subphylum);Agaricomycetes(class);Polyporales(order);
Phanerochaetaceae(family);Phanerochaete(genus);;uncultured_eukaryote
```

The first information in the descriptive line containing the complete lineage of the sequence (and the indicated taxonomic levels into parenthesis), separated by « ; » characters. The `>[C]JX396637.1.948_` information is the ID of the sequence, separated from its lineage by a « _ » character. The $[C]$ characters (for Complete) is added to indicate that all taxonomic levels (phylum, class, order, family, genus) have been described for this sequence. However, a $[I]$ for 'Incomplete' or $[E]$ for « Environmental » can be added to the descriptive line.

The database file can be split into sub files if you chose to use the USEARCH free program. All files have to be formatted using this command line:

```
user@biocomPipe:~$>
    usearch -makeudb_usearch file -output file.udb -wordlength 8
```

Their names must contain only the « .udb » suffix to be detected and used.

4.3.2 Addition of a new database organization

If the end-user wants to add a new database, or a newer version of an existing one, some modifications have to be done. For example, we want to add a new release (example: R138) of SILVA (for Bacteria). First, a new folder is created (called « R138 ») into the Version folder of the SILVA database (see Figure 4.12). Then, the needed folders are created and filled with formatted files, as described previously. Finally, some programs have to be modified to efficiently use this new database (see third part below).

4.3.3 Program files that can be impacted by a new/custom database integration

Several PERL programs and libraries have to be checked and potentially modified if the end-user wants to add a new database.

- Taxonomy.pm (PerlLib folder),
- Algae_SILVA_Taxonomy_1.1.pl (Prog_perl folder),
- Bacteria_GREENGENES_Taxonomy_2.5.pl (Prog_perl folder),
- Bacteria_SILVA_Taxonomy_2.2.pl (Prog_perl folder),
- Fungi_SILVA_Taxonomy_4.0.pl (Prog_perl folder),
- Taxo_recovery_3.2.pl (Prog_perl folder),
- Taxo_treatment_2.8.pl (Prog_perl folder).

For example, for the addition of a new release (R138) of SILVA (for Bacteria), the Bacteria_SILVA_Taxonomy_2.2.pl, have to be modified to integrate the new version of SILVA (based on the $folder_DB and $version variables). The same modification must be realized into the Taxo_treatment_2.8.pl program (based on $folder_struct and $version variables).

And if newer versions of these programs are developed (integrating the R138 version of SILVA) by the end-user, the Taxo_recovery_3.3.pl program and the Taxonomy.pm library must be modified accordingly, as these files call the previous ones.

Some other topics 5

5.1 How do I cite

If you would like to cite a paper, please cite the BIOCOM-PIPE 19.0 application note in *BMC Bioinformatics*:
BIOCOM-PIPE: a new user-friendly metabarcoding pipeline for the characterization of microbial diversity from 16S, 18S and 23S rRNA gene amplicons.

5.2 How do I report a bug?

Email us, at sebastien.terrat@inrae.fr.

Bibliography

Lynch, Michael DJ and Josh D Neufeld (2015). « Ecology and exploration of the rare biosphere ». In: *Nature Reviews Microbiology* 13.4, pp. 217–229 (cit. on pp. 10, 11, 18).

Magoč, Tanja and Steven L Salzberg (2011). « FLASH: fast length adjustment of short reads to improve genome assemblies ». In: *Bioinformatics* 27.21, pp. 2957–2963 (cit. on p. 4).

Nawrocki, Eric P and Sean R Eddy (2013). « Infernal 1.1: 100-fold faster RNA homology searches ». In: *Bioinformatics* 29.22, pp. 2933–2935 (cit. on pp. 10–12, 18, 19).

Sadet-Bourgeteau, Sophie, Sabine Houot, Samuel Dequiedt, *et al.* (2018). « Lasting effect of repeated application of organic waste products on microbial communities in arable soils ». In: *Applied soil ecology* 125, pp. 278–287 (cit. on p. 35).

Schmieder, Robert and Robert Edwards (2011). « Quality control and preprocessing of metagenomic datasets ». In: *Bioinformatics* 27.6, pp. 863–864 (cit. on p. 3).

Terrat, Sébastien, Christophe Djemiel, Corentin Journay, *et al.* (2020). « ReClustOR: a reclustering tool using an open-reference method that improves operational taxonomic unit definition ». In: *Methods in Ecology and Evolution* 11.1, pp. 168–180 (cit. on p. 23).
