# Supplementary figures and images for: BIOCOM-PIPE: a new user-friendly metabarcoding pipeline for the characterization of microbial diversity from 16S, 18S and 23S rRNA gene amplicons
Source: BMC Bioinformatics. 2020 Oct 31;21:492. doi: 10.1186/s12859-020-03829-3 (PMC7603665; doi:10.1186/s12859-020-03829-3)

# Additional File 2 : Figure S1

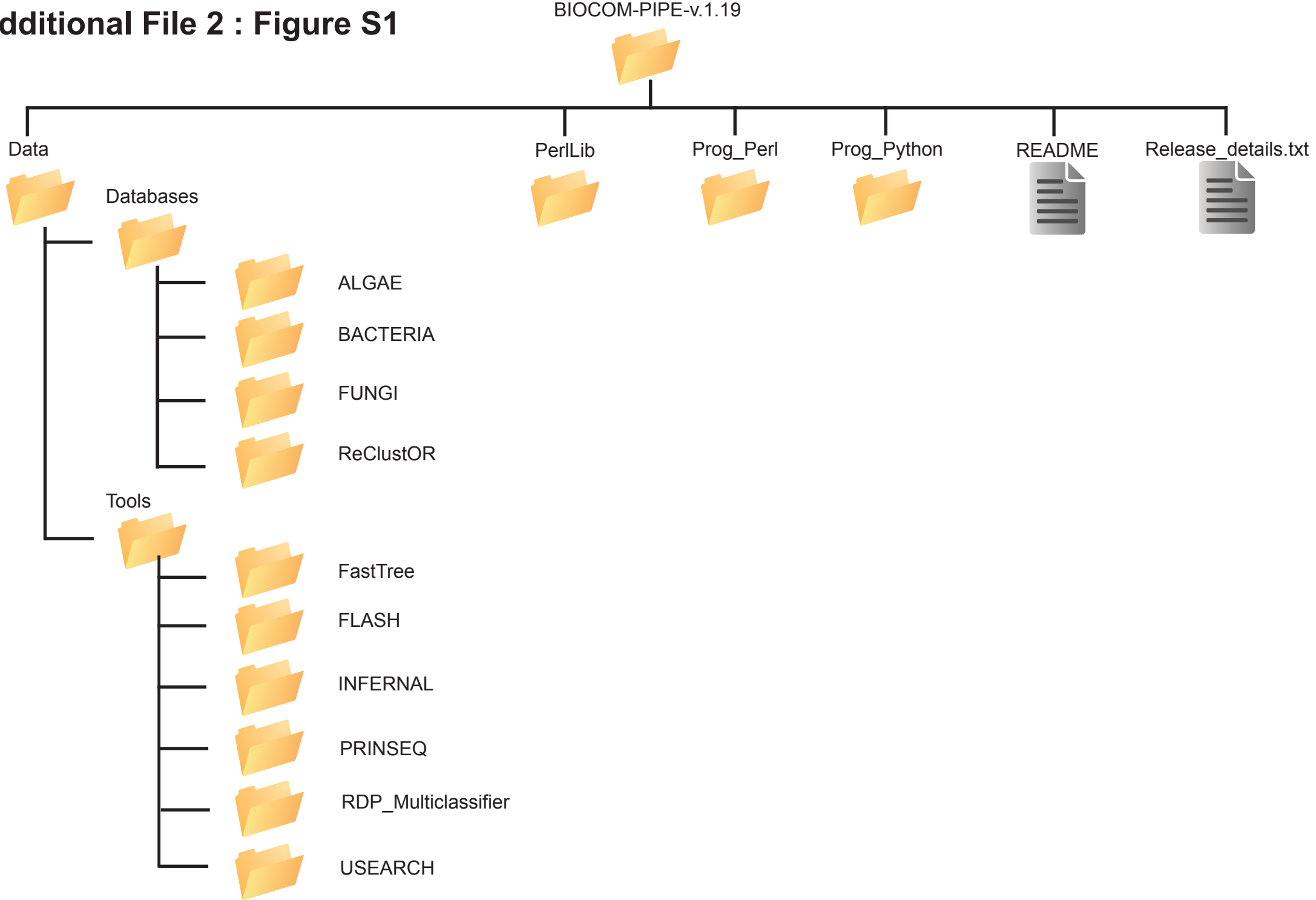

Supplement: Supplementary file 2 — Additional file 2. Figure S1. Folder structure of the BIOCOM-PIPE pipeline. [file 12859_2020_3829_MOESM2_ESM.pdf]

# Additional File 4 : Figure S3

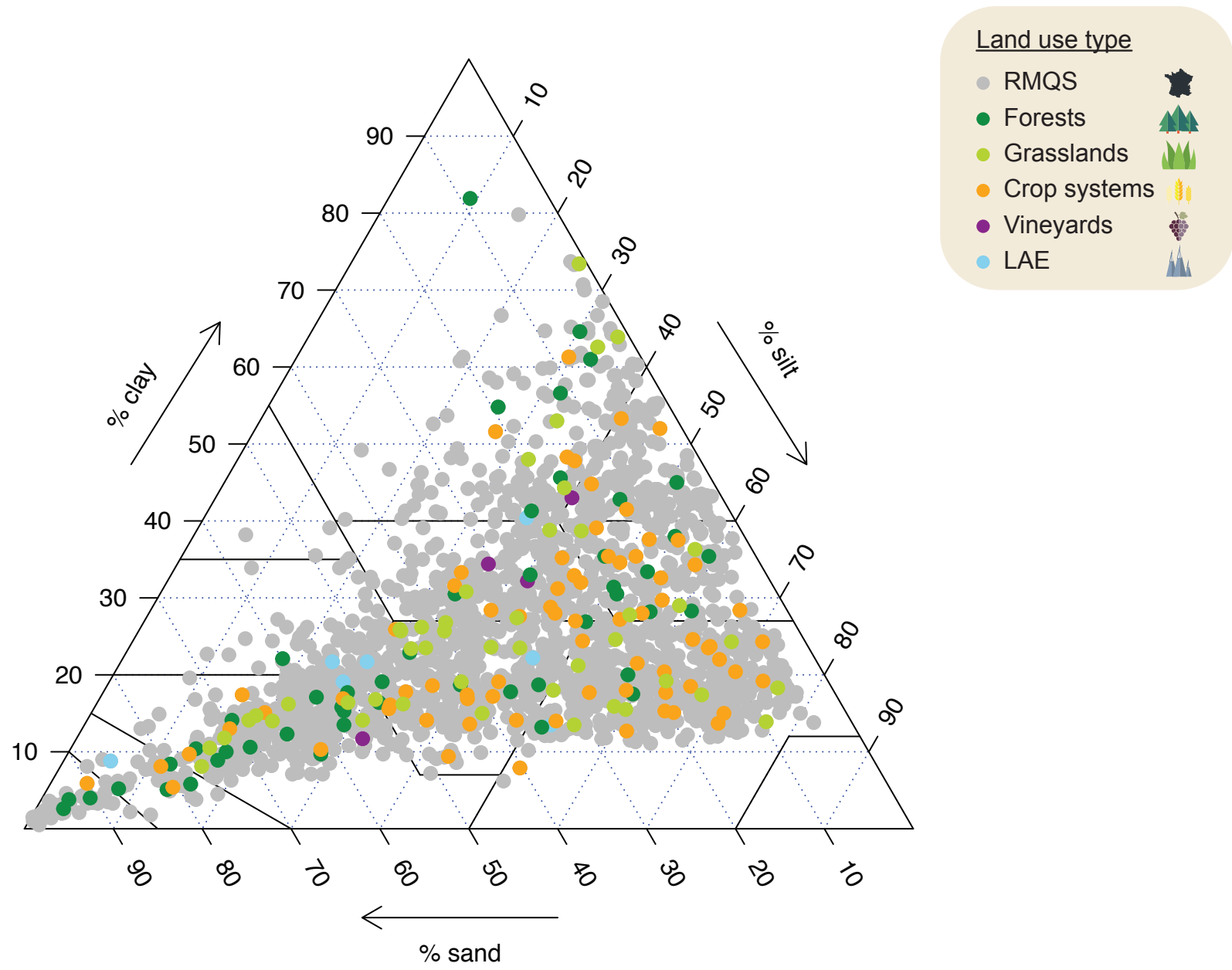

Supplement: Supplementary file 4 — Additional file 4. Figure S3. Soil texture triangle of the RMQS subsample. [file 12859_2020_3829_MOESM4_ESM.pdf]

**A****Number of detected OTUs**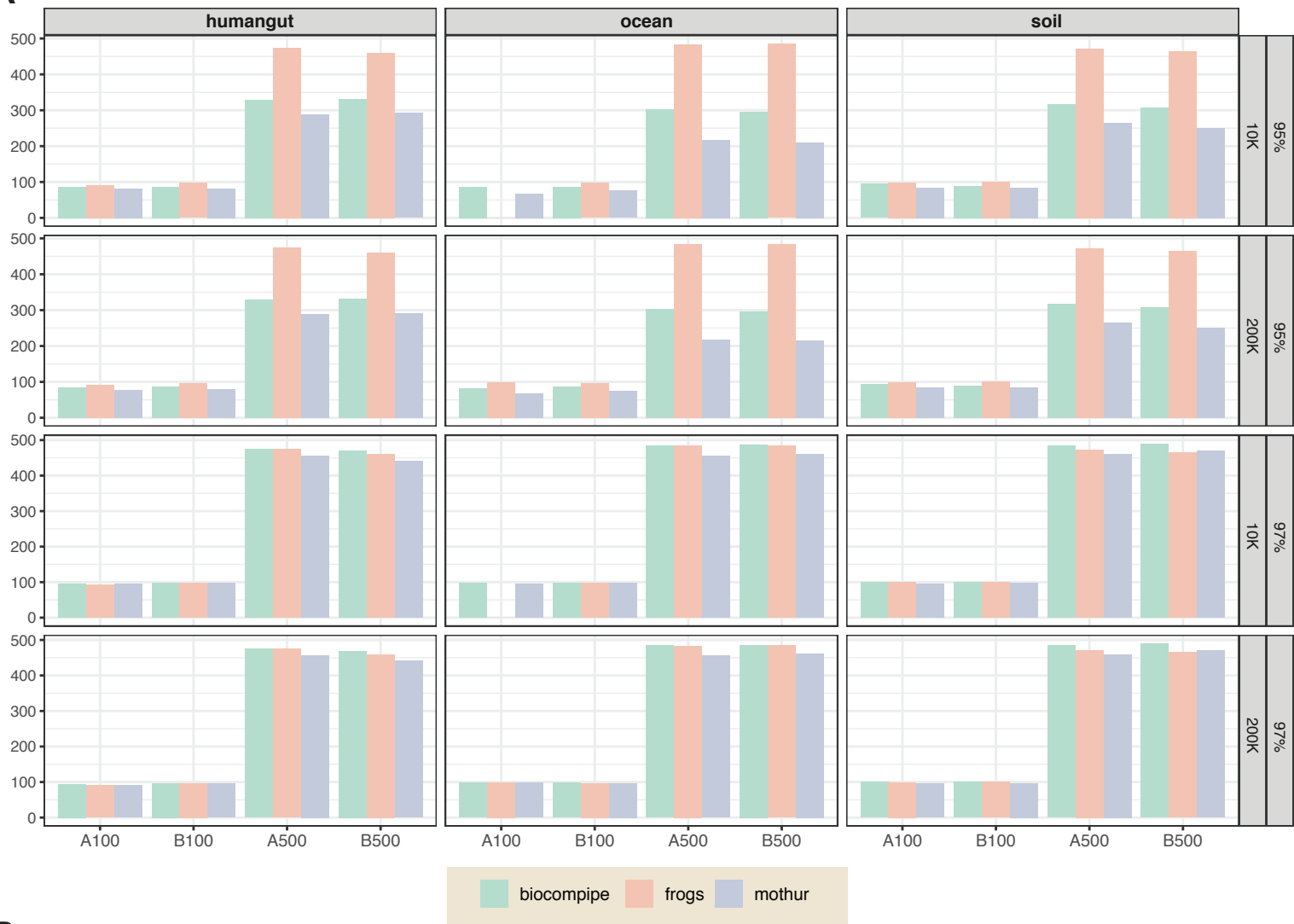**B****Number of detected organisms at the chosen level**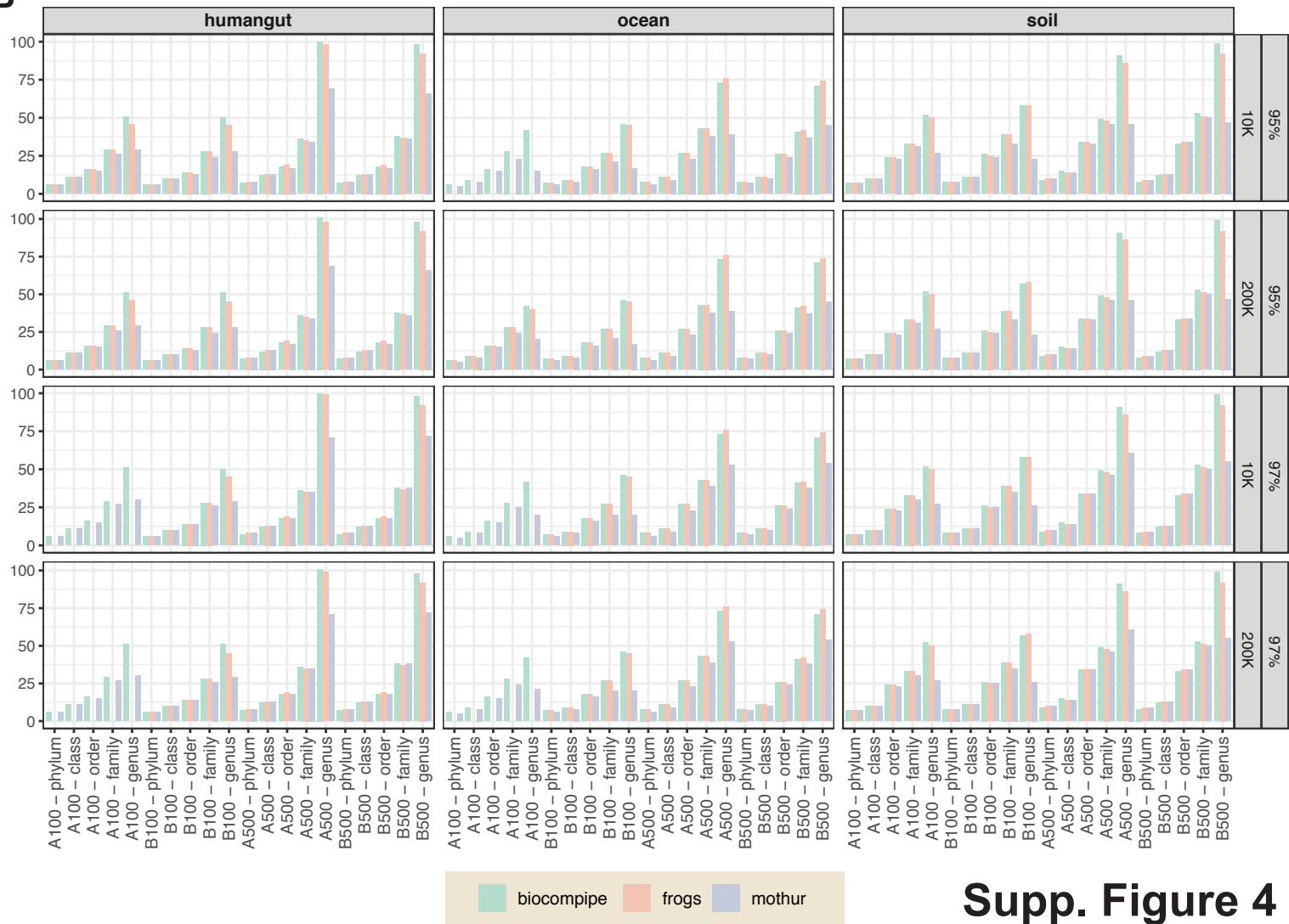**Supp. Figure 4**

Supplement: Supplementary file 6 — Additional file 6. Figure S4. (A) Number of OTUs detected for the simulated dataset, with various pipelines and clustering thresholds. (B) Number of taxonomic groups detected for each taxonomic level (phylum, class, order, family, genus), with various pipelines and clustering thresholds. [file 12859_2020_3829_MOESM6_ESM.pdf]

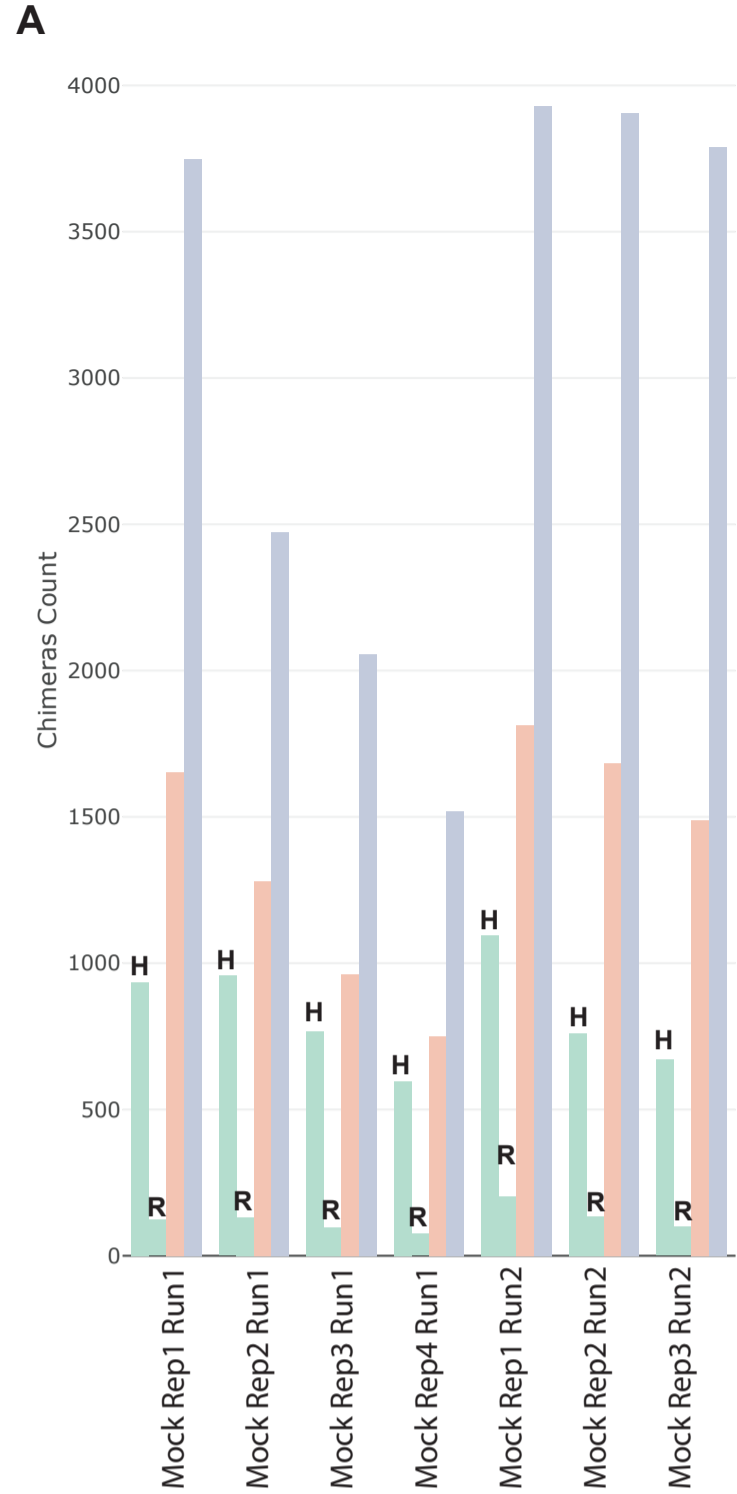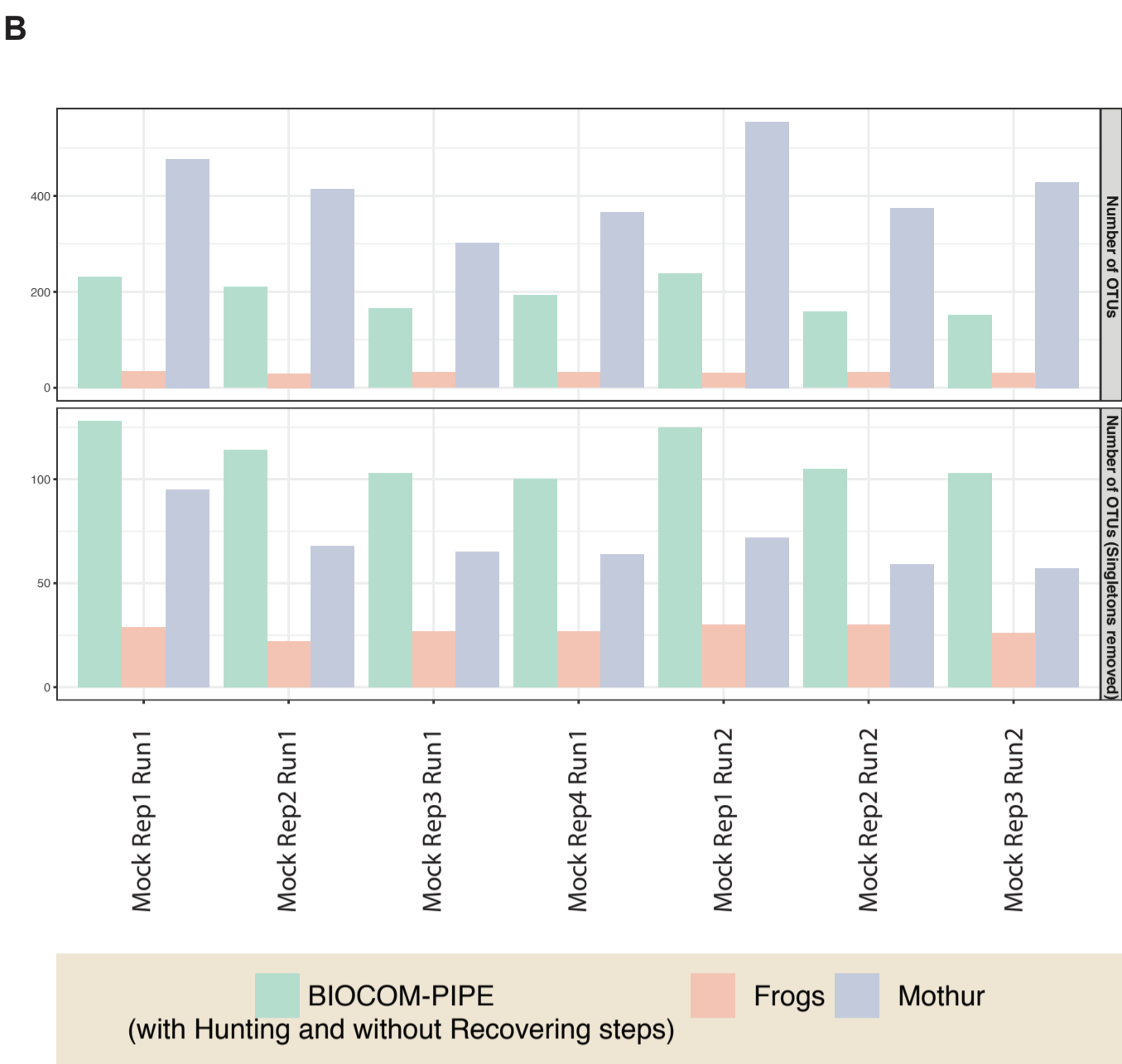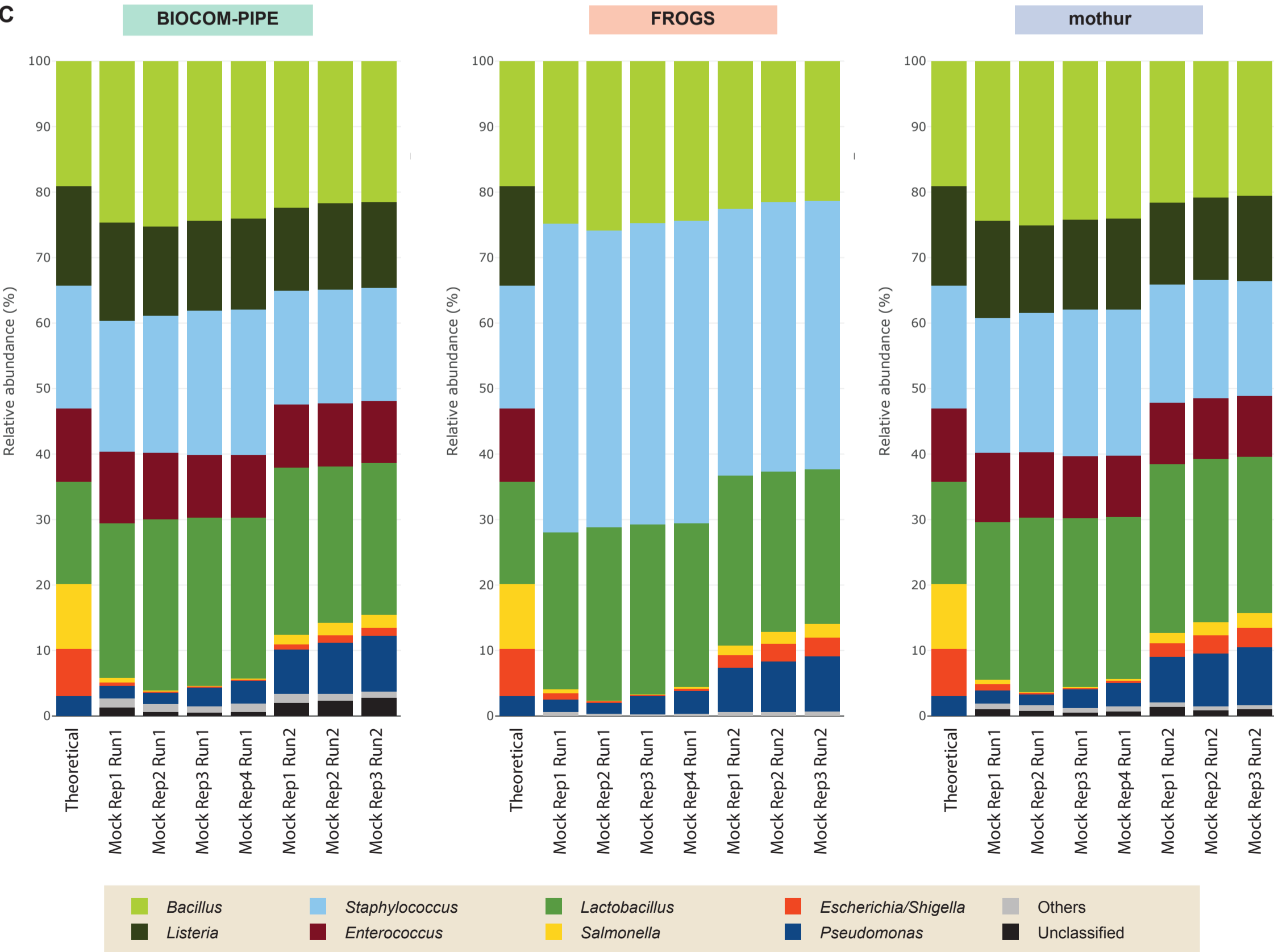

Supp. Figure 5

Supplement: Supplementary file 7 — Additional file 7. Figure S5. (A) Number of detected chimera for the artificial mock dataset, with various pipelines. For BIOCOM-PIPE, both the Hunting step (H) and the Hunting-Recovering step (R) were given. (B) Number of OTUs (with and without singletons) detected, for the artificial mock dataset, with various pipelines. (C)Taxonomic composition for the artificial mock dataset, with various pipelines. The theoretical value was given as comparison. [file 12859_2020_3829_MOESM7_ESM.pdf]
