# Supplementary material for: BIOCOM-PIPE: a new user-friendly metabarcoding pipeline for the characterization of microbial diversity from 16S, 18S and 23S rRNA gene amplicons
Source: BMC Bioinformatics. 2020 Oct 31;21:492. doi: 10.1186/s12859-020-03829-3 (PMC7603665; doi:10.1186/s12859-020-03829-3)

Additional File 3 : Figure S2

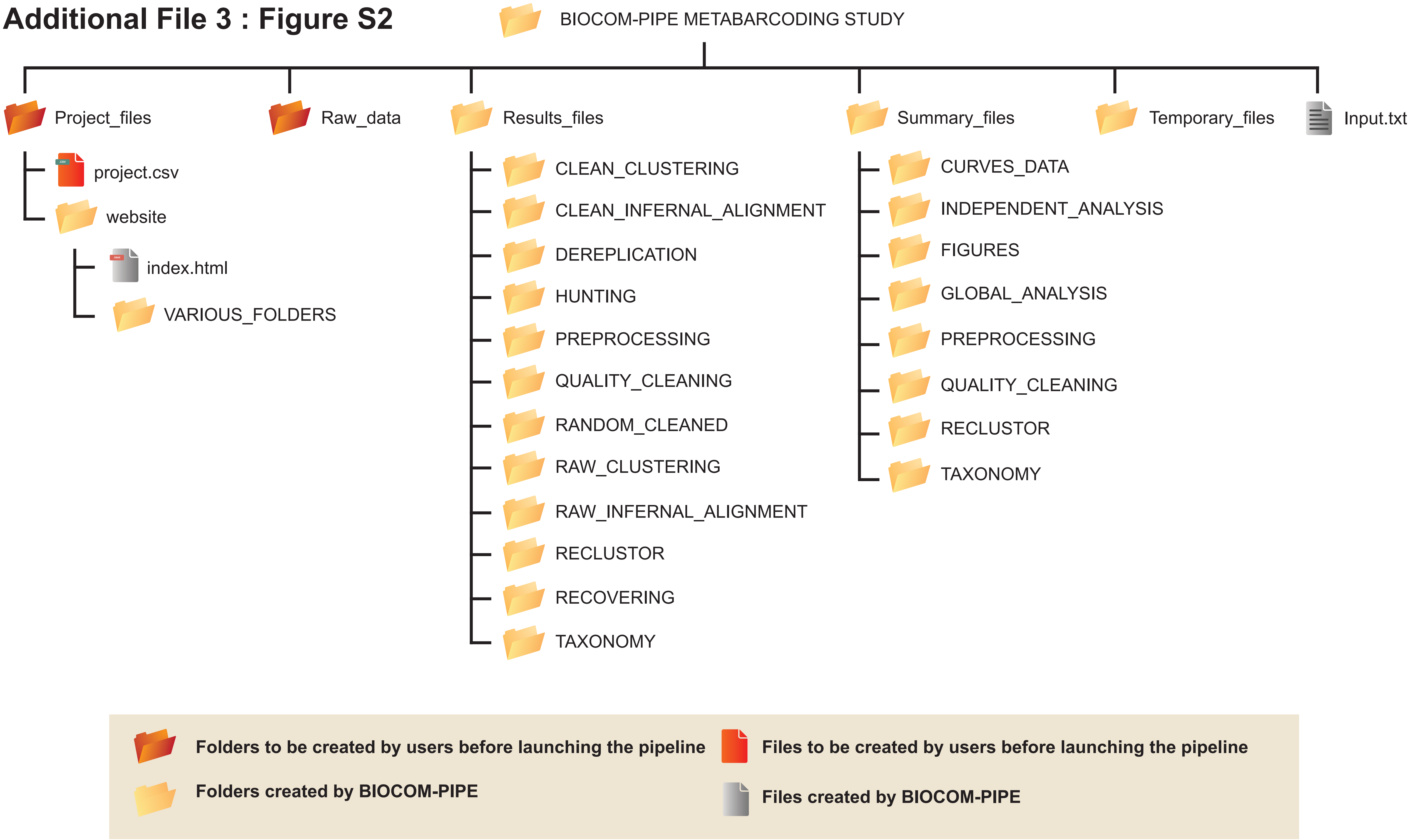

Supplement: Supplementary file 3 — Additional file 3. Figure S2. Folder structure of BIOCOM-PIPE analysis. [file 12859_2020_3829_MOESM3_ESM.pdf]
