# Supplementary material for: BIOCOM-PIPE: a new user-friendly metabarcoding pipeline for the characterization of microbial diversity from 16S, 18S and 23S rRNA gene amplicons
Source: BMC Bioinformatics. 2020 Oct 31;21:492. doi: 10.1186/s12859-020-03829-3 (PMC7603665; doi:10.1186/s12859-020-03829-3)
Supplement: Supplementary file 5 — Additional file 5. Doc S2. Materials and methods, details. [file 12859_2020_3829_MOESM5_ESM.docx]

BIOCOM-PIPE: a new user-friendly metabarcoding pipeline for the characterization of microbial diversity from 16S, 18S and 23S rRNA gene amplicons

Christophe Djemiel^1^, Samuel Dequiedt^1^, Battle Karimi^1^, Aurélien Cottin, Thibault Girier, Yassin El Djoudi, Patrick Wincker, Samuel Mondy, Nicolas Chemidlin Prévost-Bouré^1^, Pierre-Alain Maron^1^, Lionel Ranjard^1^, Sébastien Terrat^1,2^

**Corresponding author**

Correspondence to Sébastien TERRAT

sebastien.terrat@inra.fr

1. Parameters of pipelines
   1. BIOCOM-PIPE

###PRINSEQ###

Step to do [yes-no]: no

Lowest quality score tolerated for the trimming from the 3'-end of the read [0-40]: 30

Lowest quality score tolerated for the trimming from the 5'-end of the read [0-40]: 30

Minimum Length threshold tolerated to keep reads (default: 30): 30

Number of ambiguities (N's) tolerated (default: 1

Sliding window size used to calculate quality score [1-7]: 7

Step size used to move the sliding window [1-10]: 1

//

###FLASH###

Step to do [yes-no]: no

Minimum overlap length between two reads (default: 10) [10-100]: 10

Maximum overlap length between two reads (default: 65) [10-100]: 65

Maximum allowed ratio between the number of mismatched base pairs and the overlap length (default: 0.04) [0-100]: 0.04

//

###EVAL_QUAL###

Step to do [yes-no]: no

Length: 300

//

###PREPROCESS_MIDS###

Step to do [yes-no]: yes

//

###RANDOM_PREPROCESS_MIDS###

Step to do [yes-no]: no

Number of reads in the subset: 10000

//

###PREPROCESSING###

Step to do [yes-no]: yes

Minimum Length threshold: 350

Number of ambiguities (N's) tolerated: 0

Forward primer: CAGCMGCYGCNGTAANAC

Reverse primer: CCGYCAATTCMTTTRAGT

Number of differences tolerated in your forward primer sequence [0-2]: 2

Number of differences tolerated in your reverse primer sequence [0-2]: 2

Stringency [High, Medium or Low]: High

//

###QUALITY_CLEANING###

Step to do [yes-no]: no

Lowest quality score tolerated [0-40] (default: 0): 12

Lowest average quality score tolerated [0-40] (default: 0): 30

//

###RANDOM_PREPROCESS###

Step to do [yes-no]: no

Number of reads in the subset: 10000

//

###LENGTH_RANGE_PREPROCESS###

Step to do [yes-no]: no

Length: 350

//

###RAW_INFERNAL_ALIGNMENT###

Step to do [yes-no]: yes

Covariance model used [bacteria, archaea, algae or fungi]: bacteria

//

###FILTERING_RAW_ALIGNMENT###

Step to do [yes-no]: no

Quality alignment threshold (read percentage not aligned to the structure, default:10%) [1-99]: 10

//

###RAW_CLUSTERING###

Step to do [yes-no]: yes

Ignoring homopolymers differences [yes-no]: yes

Ignoring distances at the beginning of sequences [yes-no]: yes

Maximum percentage of dissimilarity for clustering (%): 5.0

Step size for each cluster (%): 5.0

//

###HUNTING###

Step to do [yes-no]: yes

Chosen clustering percentage of dissimilarity for the hunting step (%): 5.0

//

###RECOVERING###

Step to do [yes-no]: yes

Organism studied [bacteria, or fungi]: bacteria

Database used for the taxonomic assignment [RDP (bacteria only), or Silva (bacteria, fungi or algae)]: Silva

Database chosen Silva version for the taxonomic assignment of bacteria (R114 or R132, by default R132)]: R132

Taxonomic level checked [domain, phylum, class, order, family or genus]: phylum

Confidence estimates threshold [0-100]: 90

Keep Taxonomy file for deleted reads [yes-no]: yes

Number of cores or processors used for each sample to do the BLAST analysis [1-32]: 1

//

###RANDOM_CLEANED###

Step to do [yes-no]: yes

Number of reads in the subset: 8000

//

###TAXONOMY###

Step to do [yes-no]: yes

Organism studied [bacteria, or fungi]: bacteria

Database used for the taxonomic assignment [RDP (bacteria only), or Silva (bacteria and fungi)]: Silva

Database chosen Silva version for the taxonomic assignment of bacteria (R114 or R132, by default R132)]: R132

Confidence estimates threshold [0-100]: 80

Number of cores or processors used for each sample to do the BLAST analysis [1-32]: 1

//

###CLEAN_INFERNAL_ALIGNMENT###

Step to do [yes-no]: yes

Covariance model used [bacteria, archaea, algae or fungi]: bacteria

//

###CLEAN_CLUSTERING###

Step to do [yes-no]: yes

Ignoring homopolymers differences [yes-no]: yes

Ignoring distances at the beginning of sequences [yes-no]: yes

Maximum percentage of dissimilarity for clustering (%): 5.0

Step size for each cluster (%): 5.0

Rank-abundance curves computation [yes-no]: yes

//

###COMPUTATION###

Step to do [yes-no]: yes

Chosen clustering step for analysis [Raw, Clean, Both]: Clean

Chosen percentage of dissimilarity for clustering (%): 5.0

Determination of rarefaction curve(s) [yes-no]: yes

Determination of rank-abundance curve(s) [yes-no]: yes

Computation of the full bias corrected Chao1 richness estimator [yes-no]: yes

Computation of the ACE richness estimator [yes-no]: yes

Computation of bootstrap estimate, and shannon and simpson indexes [yes-no]: yes

//

###GLOBAL_ANALYSIS###

Step to do [yes-no]: yes

Chosen clustering step for analysis [Raw, Clean, Both]: Clean

Chosen percentage of dissimilarity for the treated clustering (%): 5.0

Defining the taxonomic assignment of all OTUs in the global matrix [yes-no]: yes

Confidence estimates (RDP) or similarity percentage (SILVA) threshold [0-100]: 80

Realization of a phylogenetic tree and an ID mapping file for UNIFRAC analysis (time-consuming step !!) [yes-no]: yes

Selection of the most abundant read to represent each OTU for UNIFRAC analysis (tree and mapping file) [yes-no]: yes

//

###RECLUSTOR###

Step to do [yes-no]: yes

Do you want to define a new database to realize the clustering step [yes-no]: yes

Do you want to use an existing database to realize the clustering step [yes-no]: no

Do you want to enrich the used database with treated read [yes-no]: no

Database name (short name without spaces or special characters): RMQS_SUB_8000

Covariance model used [bacteria, archaea, algae or fungi]: bacteria

Chosen percentage of dissimilarity for the clustering (default 5%) [1-100]: 5.0

Ignoring homopolymers differences during the clustering [yes-no]: yes

Ignoring differences at the beginning of sequences [yes-no]: yes

//

###UNIFRAC_ANALYSIS###

Step to do [yes-no]: yes

Calculate a UniFrac Distance Matrix and apply PCoA and UPGMA [yes-no]: yes

Compute the Phylogenetic Diversity (PD) of all samples [yes-no]: yes

Use abundance weigths (select whether the abundance of reads will be used or not for UniFrac tests) [yes-no]: yes

//

- 1. mothur’s

mothur > align.seqs(fasta=subdata_rmqs.unique.fasta,reference=silva.rmqs_bacteria.fasta,outputdir=.,processors=20)

mothur > summary.seqs(fasta=subdata_rmqs.unique.align,count=subdata_rmqs.unique.count_table,processors=20)

mothur > screen.seqs(fasta=subdata_rmqs.unique.align,count=subdata_rmqs.unique.count_table,summary=subdata_rmqs.unique.summary,maxambig=0,minlength=350,maxlength=378,maxhomop=7,start=13875,end=27659,outputdir=.)

mothur > summary.seqs(fasta=subdata_rmqs.unique.good.align,count=subdata_rmqs.unique.good.count_table)

mothur > count.groups(count=subdata_rmqs.unique.good.count_table)

mothur > screen.seqs(fasta=subdata_rmqs.unique.align,count=subdata_rmqs.unique.count_table,summary=subdata_rmqs.unique.summary,maxambig=0,minlength=350,maxlength=378,maxhomop=7,optimize=start-end-minlength,criteria=90,outputdir=.)

mothur > summary.seqs(fasta=subdata_rmqs.unique.good.align,count=subdata_rmqs.unique.good.count_table)

mothur > filter.seqs(fasta=subdata_rmqs.unique.good.align,vertical=T,trump=.)

mothur > unique.seqs(fasta=subdata_rmqs.unique.good.filter.fasta,count=subdata_rmqs.unique.good.count_table)

mothur > summary.seqs(fasta=subdata_rmqs.unique.good.filter.unique.fasta,count=subdata_rmqs.unique.good.filter.count_table)

mothur > count.groups(count=subdata_rmqs.unique.good.filter.count_table)

mothur > pre.cluster(fasta=subdata_rmqs.unique.good.filter.unique.fasta,count=subdata_rmqs.unique.good.filter.count_table,diffs=3)

mothur > summary.seqs(fasta=subdata_rmqs.unique.good.filter.unique.precluster.fasta,count=subdata_rmqs.unique.good.filter.unique.precluster.count_table)

mothur > chimera.vsearch(fasta=subdata_rmqs.unique.good.filter.unique.precluster.fasta,count=subdata_rmqs.unique.good.filter.unique.precluster.count_table,dereplicate=t)

mothur > remove.seqs(fasta=subdata_rmqs.unique.good.filter.unique.precluster.fasta,accnos=subdata_rmqs.unique.good.filter.unique.precluster.denovo.vsearch.accnos)

mothur > summary.seqs(fasta=subdata_rmqs.unique.good.filter.unique.precluster.pick.fasta,count=subdata_rmqs.unique.good.filter.unique.precluster.denovo.vsearch.pick.count_table)

mothur > classify.seqs(fasta=subdata_rmqs.unique.good.filter.unique.precluster.pick.fasta,count=subdata_rmqs.unique.good.filter.unique.precluster.denovo.vsearch.pick.count_table,reference=silva.nr_v132.pcr.align,taxonomy=silva.pcr_sub.tax,cutoff=80,outputdir=.)

mothur > dist.seqs(fasta=subdata_rmqs.unique.good.filter.unique.precluster.pick.fasta,cutoff=0.05,processors=20)

mothur > cluster(column=subdata_rmqs.unique.good.filter.unique.precluster.pick.dist,count=subdata_rmqs.unique.good.filter.unique.precluster.denovo.vsearch.pick.count_table,cutoff=0.05)

mothur > classify.otu(list=subdata_rmqs.unique.good.filter.unique.precluster.pick.opti_mcc.list,count=subdata_rmqs.unique.good.filter.unique.precluster.denovo.vsearch.pick.count_table,taxonomy=subdata_rmqs.unique.good.filter.unique.precluster.pick.pcr_sub.wang.taxonomy,label=0.05)

mothur > make.shared(list=subdata_rmqs.unique.good.filter.unique.precluster.pick.opti_mcc.list,count=subdata_rmqs.unique.good.filter.unique.precluster.denovo.vsearch.pick.count_table,label=0.05)

mothur > sub.sample(shared=subdata_rmqs.unique.good.filter.unique.precluster.pick.opti_mcc.shared,size=8000)

mothur > sub.sample(constaxonomy=subdata_rmqs.unique.good.filter.unique.precluster.pick.opti_mcc.0.05.cons.taxonomy,shared=subdata_rmqs.unique.good.filter.unique.precluster.pick.opti_mcc.shared,size=8000,outputdir=.)

mothur > count.seqs(shared=subdata_rmqs.unique.good.filter.unique.precluster.pick.opti_mcc.0.05.subsample.shared)

mothur > summary.tax(taxonomy=subdata_rmqs.unique.good.filter.unique.precluster.pick.opti_mcc.0.05.cons.subsample.good.taxonomy,count=subdata_rmqs.unique.good.filter.unique.precluster.pick.opti_mcc.0.05.subsample.count_table,threshold=80)

mothur > list.otulabels(shared=subdata_rmqs.unique.good.filter.unique.precluster.pick.opti_mcc.0.05.subsample.shared)

- 1. FROGS

**FROGS Clustering swarm**

## Application

Software :/galaxydata/galaxy-prod/my_tools/FROGS/app/clustering.py (version : r3.0-1.4)

Command : /galaxydata/galaxy-prod/my_tools/FROGS/app/clustering.py --nb-cpus 16 --distance 3 --input-fasta /galaxydata/galaxy-prod/my_files/000/406/dataset_406905.dat --input-count /galaxydata/galaxy-prod/my_files/000/406/dataset_406908.dat --output-biom /galaxydata/galaxy-prod/my_job_working_directory/000/248/248316/galaxy_dataset_480134.dat --output-fasta /galaxydata/galaxy-prod/my_job_working_directory/000/248/248316/galaxy_dataset_480133.dat --output-compo /galaxydata/galaxy-prod/my_job_working_directory/000/248/248316/galaxy_dataset_480135.dat --denoising

########################################################################################################

# Sort pre-clusters by abundancies. (sortAbundancies.py version : 1.5.0)

Command:

sortAbundancies.py --size-separator ';size=' --input-file /galaxydata/galaxy-prod/my_files/000/406/dataset_406905.dat --output-file /galaxydata/galaxy-prod/my_job_working_directory/000/248/248316/1561964199.36_50524_dataset_406905_sorted.fasta

repalce N tags by A. in: /galaxydata/galaxy-prod/my_job_working_directory/000/248/248316/1561964199.36_50524_dataset_406905_sorted.fasta out : /galaxydata/galaxy-prod/my_job_working_directory/000/248/248316/1561964199.36_50524_dataset_406905_sorted_NtoA.fasta

########################################################################################################

# Clustering sequences. (swarm version : 2.1.1)

Command:

swarm --differences 1 --threads 16 --log /galaxydata/galaxy-prod/my_job_working_directory/000/248/248316/1561964199.36_50524_dataset_406905_denoising_log.txt --output-file /galaxydata/galaxy-prod/my_job_working_directory/000/248/248316/1561964199.36_50524_dataset_406905_denoising_composition.txt /galaxydata/galaxy-prod/my_job_working_directory/000/248/248316/1561964199.36_50524_dataset_406905_sorted_NtoA.fasta

########################################################################################################

# Extracts seeds sequences to produce the seeds fasta. (extractSwarmsFasta.py version : 1.4.1)

Command:

extractSwarmsFasta.py --input-fasta /galaxydata/galaxy-prod/my_job_working_directory/000/248/248316/1561964199.36_50524_dataset_406905_sorted_NtoA.fasta --input-swarms /galaxydata/galaxy-prod/my_job_working_directory/000/248/248316/1561964199.36_50524_dataset_406905_denoising_composition.txt --output-fasta /galaxydata/galaxy-prod/my_job_working_directory/000/248/248316/1561964199.36_50524_dataset_406905_denoising_seeds.fasta

########################################################################################################

# Sort pre-clusters by abundancies. (sortAbundancies.py version : 1.5.0)

Command:

sortAbundancies.py --size-separator '_' --input-file /galaxydata/galaxy-prod/my_job_working_directory/000/248/248316/1561964199.36_50524_dataset_406905_denoising_resizedSeeds.fasta --output-file /galaxydata/galaxy-prod/my_job_working_directory/000/248/248316/1561964199.36_50524_dataset_406905_denoising_sortedSeeds.fasta

########################################################################################################

# Clustering sequences. (swarm version : 2.1.1)

Command:

swarm --differences 3 --threads 16 --log /galaxydata/galaxy-prod/my_job_working_directory/000/248/248316/1561964199.36_50524_dataset_406905_swarm_log.txt --output-file /galaxydata/galaxy-prod/my_job_working_directory/000/248/248316/1561964199.36_50524_dataset_406905_swarmD3_composition.txt /galaxydata/galaxy-prod/my_job_working_directory/000/248/248316/1561964199.36_50524_dataset_406905_denoising_sortedSeeds.fasta

########################################################################################################

# Converts swarm output to abundance file (format BIOM). (swarm2biom.py version : 1.4.0)

Command:

swarm2biom.py --clusters-file /galaxydata/galaxy-prod/my_job_working_directory/000/248/248316/galaxy_dataset_480135.dat --count-file /galaxydata/galaxy-prod/my_files/000/406/dataset_406908.dat --output-file /galaxydata/galaxy-prod/my_job_working_directory/000/248/248316/galaxy_dataset_480134.dat

########################################################################################################

# Extracts seeds sequences to produce the seeds fasta. (extractSwarmsFasta.py version : 1.4.1)

Command:

extractSwarmsFasta.py --input-fasta /galaxydata/galaxy-prod/my_job_working_directory/000/248/248316/1561964199.36_50524_dataset_406905_denoising_sortedSeeds.fasta --input-swarms /galaxydata/galaxy-prod/my_job_working_directory/000/248/248316/1561964199.36_50524_dataset_406905_swarmD3_composition.txt --output-fasta /galaxydata/galaxy-prod/my_job_working_directory/000/248/248316/1561964199.36_50524_dataset_406905_final_seeds.fasta

repalce A tags by N. in: /galaxydata/galaxy-prod/my_job_working_directory/000/248/248316/1561964199.36_50524_dataset_406905_final_seeds.fasta out : /galaxydata/galaxy-prod/my_job_working_directory/000/248/248316/galaxy_dataset_480133.dat

Epilog : job finished at Mon Jul 1 09:33:01 CEST 2019

**FROGS Clusters stat**

## Application

Software :/galaxydata/galaxy-prod/my_tools/FROGS/app/clusters_stat.py (version : r3.0-3.0)

Command : /galaxydata/galaxy-prod/my_tools/FROGS/app/clusters_stat.py --input-biom /galaxydata/galaxy-prod/my_files/000/406/dataset_406910.dat --output-file /galaxydata/galaxy-prod/my_job_working_directory/000/248/248319/galaxy_dataset_480143.dat

########################################################################################################

# Hierarchical classification on observation proportions. (biomTools.py version : 0.10.1)

Command:

biomTools.py hclassification --distance-method braycurtis --linkage-method average --input-file /galaxydata/galaxy-prod/my_files/000/406/dataset_406910.dat --output-file /galaxydata/galaxy-prod/my_job_working_directory/000/248/248319/1561967044.32_31842_HClassif.newick > /galaxydata/galaxy-prod/my_job_working_directory/000/248/248319/1561967044.32_31842_HClassif_log.txt

########################################################################################################

# Writes by abundance the number of clusters. (biomTools.py version : 0.10.1)

Command:

biomTools.py obsdepth --input-file /galaxydata/galaxy-prod/my_files/000/406/dataset_406910.dat --output-file /galaxydata/galaxy-prod/my_job_working_directory/000/248/248319/1561967044.32_31842_depths.tsv

**FROGS Remove chimera**

## Application

Software :/galaxydata/galaxy-prod/my_tools/FROGS/app/remove_chimera.py (version : r3.0-7.0)

Command : /galaxydata/galaxy-prod/my_tools/FROGS/app/remove_chimera.py --nb-cpus 12 --input-fasta /galaxydata/galaxy-prod/my_files/000/406/dataset_406909.dat --non-chimera /galaxydata/galaxy-prod/my_job_working_directory/000/248/248320/galaxy_dataset_480144.dat --summary /galaxydata/galaxy-prod/my_job_working_directory/000/248/248320/galaxy_dataset_480146.dat --input-biom /galaxydata/galaxy-prod/my_files/000/406/dataset_406910.dat --out-abundance /galaxydata/galaxy-prod/my_job_working_directory/000/248/248320/galaxy_dataset_480145.dat

########################################################################################################

# Removes PCR chimera by samples. (parallelChimera.py version : 0.7.1 [vsearch v2.6.2_linux_x86_64])

Command:

parallelChimera.py --lenient-filter --nb-cpus 12 --sequences /galaxydata/galaxy-prod/my_files/000/406/dataset_406909.dat --biom /galaxydata/galaxy-prod/my_files/000/406/dataset_406910.dat --non-chimera /galaxydata/galaxy-prod/my_job_working_directory/000/248/248320/galaxy_dataset_480144.dat --out-abundance /galaxydata/galaxy-prod/my_job_working_directory/000/248/248320/galaxy_dataset_480145.dat --summary /galaxydata/galaxy-prod/my_job_working_directory/000/248/248320/1561967414.27_53551_galaxy_dataset_480144.dat_summary.tsv --log-file /galaxydata/galaxy-prod/my_job_working_directory/000/248/248320/1561967414.27_53551_galaxy_dataset_480144.dat_tmp.log

Adapt for all samples

## Vsearch command: vsearch --uchime_denovo /galaxydata/galaxy-prod/my_job_working_directory/000/248/248320/1561967414.64_53554_100007C18_B024.fasta.tmp --nonchimeras /galaxydata/galaxy-prod/my_job_working_directory/000/248/248320/1561967414.64_53554_100007C18_B024.fasta --uchimeout /galaxydata/galaxy-prod/my_job_working_directory/000/248/248320/1561967414.64_53554_100007C18_B024.fasta.log

**FROGS Affiliation OTU**

## Application

Software: affiliation_OTU.py (version: r3.0-2.0)

Command: /galaxydata/galaxy-prod/my_tools/FROGS/app/affiliation_OTU.py --reference /galaxydata/galaxy_bank/FROGS_galaxy_databanks/16S/silva_132/silva_132_16S.fasta --input-biom /galaxydata/galaxy-prod/my_files/000/406/dataset_406921.dat --input-fasta /galaxydata/galaxy-prod/my_files/000/406/dataset_406920.dat --output-biom /galaxydata/galaxy-prod/my_job_working_directory/000/252/252753/galaxy_dataset_487793.dat --summary /galaxydata/galaxy-prod/my_job_working_directory/000/252/252753/galaxy_dataset_487794.dat --nb-cpus 30 --java-mem 20

Nb seq : 335690

########################################################################################################

# blast taxonomic affiliation (blastn version : 2.2.30+)

Command:

blastn -num_threads 30 -task megablast -word_size 38 -max_target_seqs 500 -outfmt '6 qseqid sseqid pident length mismatch gapopen qstart qend sstart send evalue bitscore qlen' -query /galaxydata/galaxy-prod/my_job_working_directory/000/252/252753/1562856547.3_51077_dataset_406920.dat_FROGS_full_length -out /galaxydata/galaxy-prod/my_job_working_directory/000/252/252753/1562856547.3_51077_1562856547.3_51077_dataset_406920.dat_FROGS_full_length.blast -db /galaxydata/galaxy_bank/FROGS_galaxy_databanks/16S/silva_132/silva_132_16S.fasta

########################################################################################################

# Add Blast and/or RDP affiliation to biom (addAffiliation2biom.py version : 2.3.0)

Command:

addAffiliation2biom.py -f /galaxydata/galaxy_bank/FROGS_galaxy_databanks/16S/silva_132/silva_132_16S.fasta -i /galaxydata/galaxy-prod/my_files/000/406/dataset_406921.dat -o /galaxydata/galaxy-prod/my_job_working_directory/000/252/252753/galaxy_dataset_487793.dat -b /galaxydata/galaxy-prod/my_job_working_directory/000/252/252753/1562856547.3_51077_1562856547.3_51077_dataset_406920.dat_FROGS_full_length.blast

**FROGS Filters**

## Application

Software: filters.py (version: r3.0-3.2)

Command: /galaxydata/galaxy-prod/my_tools/FROGS/app/filters.py --nb-cpus 1 --input-biom /galaxydata/galaxy-prod/my_files/000/413/dataset_413874.dat --input-fasta /galaxydata/galaxy-prod/my_files/000/406/dataset_406920.dat --output-fasta /galaxydata/galaxy-prod/my_job_working_directory/000/253/253038/galaxy_dataset_488464.dat --output-biom /galaxydata/galaxy-prod/my_job_working_directory/000/253/253038/galaxy_dataset_488465.dat --excluded /galaxydata/galaxy-prod/my_job_working_directory/000/253/253038/galaxy_dataset_488466.dat --summary /galaxydata/galaxy-prod/my_job_working_directory/000/253/253038/galaxy_dataset_488467.dat --min-blast-identity 0.98 --min-blast-coverage 0.95

########################################################################################################

# Updates fasta file based on sequence in biom file. (biomFastaUpdate.py version : 1.0.1)

Command:

biomFastaUpdate.py --input-biom /galaxydata/galaxy-prod/my_job_working_directory/000/253/253038/galaxy_dataset_488465.dat --input-fasta /galaxydata/galaxy-prod/my_files/000/406/dataset_406920.dat --output-file /galaxydata/galaxy-prod/my_job_working_directory/000/253/253038/galaxy_dataset_488464.dat --log /galaxydata/galaxy-prod/my_job_working_directory/000/253/253038/1562961825.77_12996_update_fasta_log.txt

**FROGS Affiliations stat**

## Application

Software: affiliations_stat.py (version: r3.0-3.1)

Command: /galaxydata/galaxy-prod/my_tools/FROGS/app/affiliations_stat.py --input-biom /galaxydata/galaxy-prod/my_files/000/414/dataset_414453.dat --output-file /galaxydata/galaxy-prod/my_job_working_directory/000/253/253039/galaxy_dataset_488468.dat --rarefaction-ranks Class Order Family Genus Species --taxonomic-ranks Domain Phylum Class Order Family Genus Species --multiple-tag blast_affiliations --tax-consensus-tag blast_taxonomy --identity-tag perc_identity --coverage-tag perc_query_coverage

########################################################################################################

# Writes by sample the rarefaction data for rank(s) 2, 3, 4, 5, 6. (biomTools.py version : 0.10.1)

Command:

biomTools.py rarefaction --input-file /galaxydata/galaxy-prod/my_files/000/414/dataset_414453.dat --output-file-pattern /galaxydata/galaxy-prod/my_job_working_directory/000/253/253039/1562962974.08_87765_rarefaction_rank_##RANK##.tsv --taxonomy-key "blast_taxonomy" --step-size 324 --ranks 2 3 4 5 6

########################################################################################################

# Produces a taxonomy tree with counts by sample. (biomTools.py version : 0.10.1)

Command:

biomTools.py treeCount --input-file /galaxydata/galaxy-prod/my_files/000/414/dataset_414453.dat --taxonomy-key "blast_taxonomy" --output-enewick /galaxydata/galaxy-prod/my_job_working_directory/000/253/253039/1562962974.08_87765_taxCount.enewick --output-samples /galaxydata/galaxy-prod/my_job_working_directory/000/253/253039/1562962974.08_87765_taxCount_ids.tsv

**FROGS Abundance normalisation**

## Application

Software: normalisation.py (version: r3.0-8.0)

Command: /galaxydata/galaxy-prod/my_tools/FROGS/app/normalisation.py --input-biom /galaxydata/galaxy-prod/my_files/000/414/dataset_414453.dat --input-fasta /galaxydata/galaxy-prod/my_files/000/414/dataset_414452.dat --num-reads 8000 --output-biom /galaxydata/galaxy-prod/my_job_working_directory/000/253/253042/galaxy_dataset_488478.dat --output-fasta /galaxydata/galaxy-prod/my_job_working_directory/000/253/253042/galaxy_dataset_488477.dat --summary-file /galaxydata/galaxy-prod/my_job_working_directory/000/253/253042/galaxy_dataset_488479.dat

########################################################################################################

# Random sampling in each sample. (biomTools.py version : 0.10.1)

Command:

biomTools.py sampling --nb-sampled 8000 --input-file /galaxydata/galaxy-prod/my_files/000/414/dataset_414453.dat --output-file /galaxydata/galaxy-prod/my_job_working_directory/000/253/253042/galaxy_dataset_488478.dat

########################################################################################################

# Update fasta file based on sequence in biom file (biomFastaUpdate.py version : 1.0.1)

Command:

biomFastaUpdate.py --input-biom /galaxydata/galaxy-prod/my_job_working_directory/000/253/253042/galaxy_dataset_488478.dat --input-fasta /galaxydata/galaxy-prod/my_files/000/414/dataset_414452.dat --output-file /galaxydata/galaxy-prod/my_job_working_directory/000/253/253042/galaxy_dataset_488477.dat --log /galaxydata/galaxy-prod/my_job_working_directory/000/253/253042/1563045288.26_82963_tmp_fasta_update.log
