# Supplementary material for: BIOCOM-PIPE: a new user-friendly metabarcoding pipeline for the characterization of microbial diversity from 16S, 18S and 23S rRNA gene amplicons
Source: BMC Bioinformatics. 2020 Oct 31;21:492. doi: 10.1186/s12859-020-03829-3 (PMC7603665; doi:10.1186/s12859-020-03829-3)

Supp. Figure 6

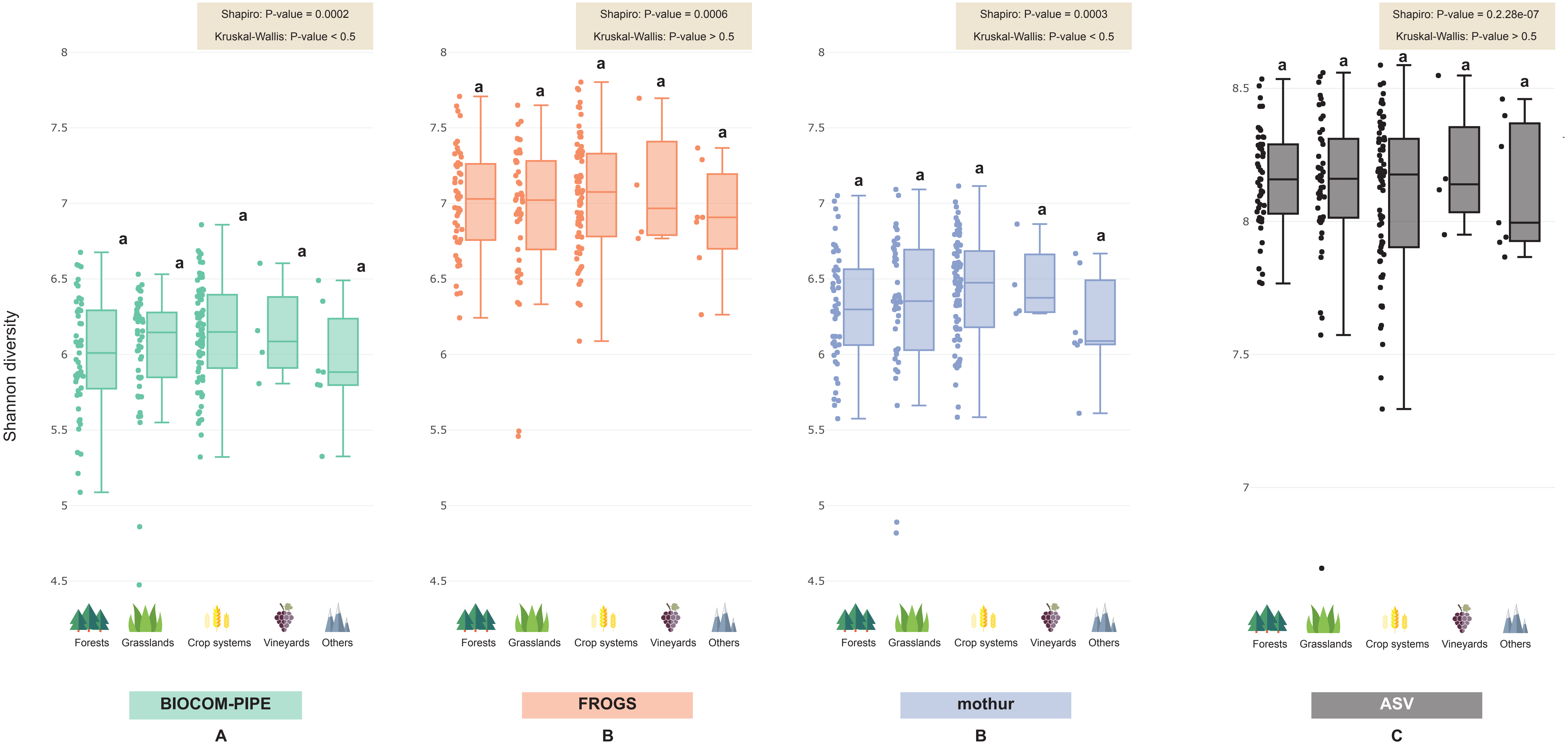

Supp. Figure 7

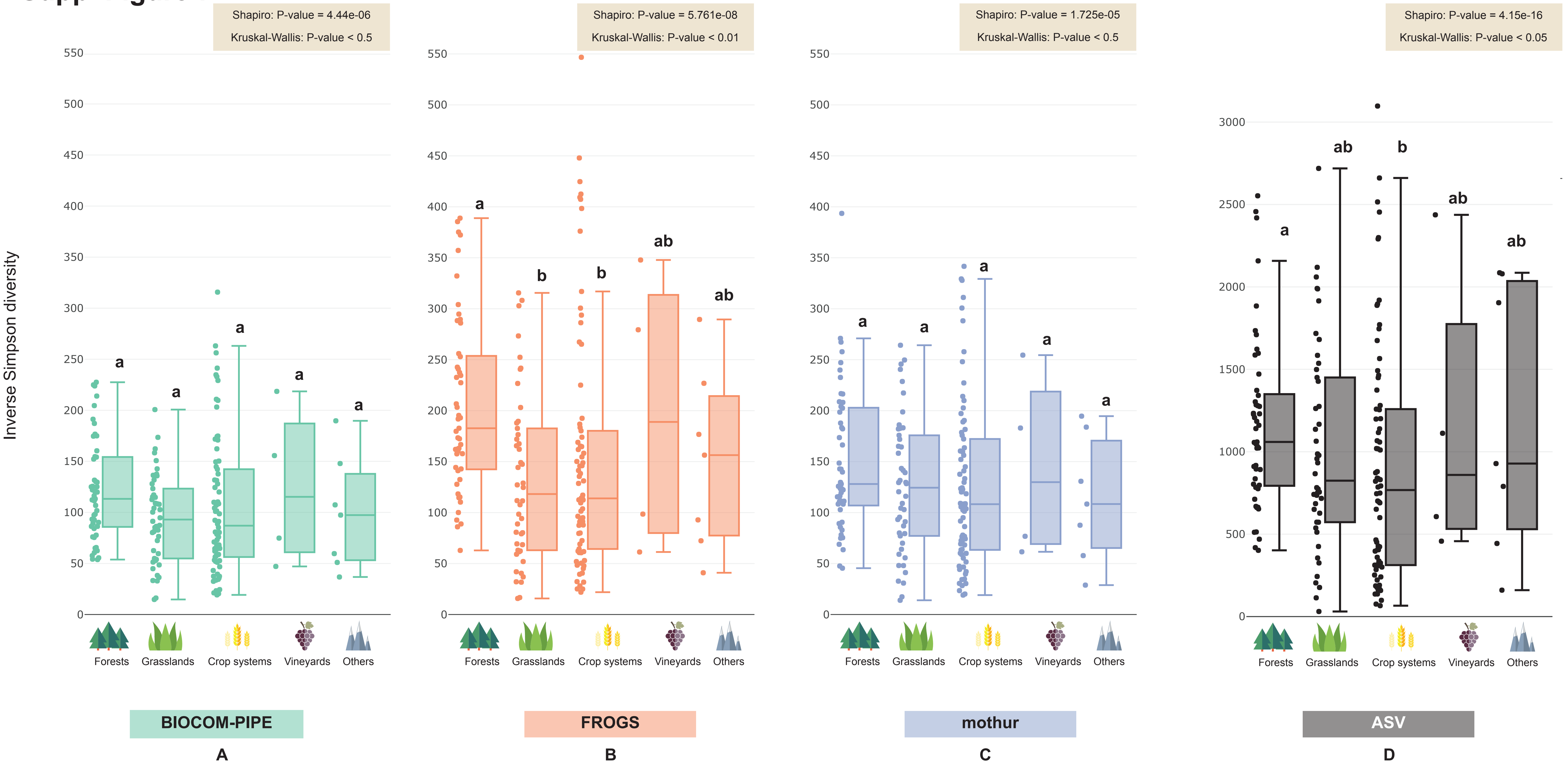

Supplement: Supplementary file 8 — Additional file 8. Figure S4. Boxplots of Shannon indexes for each land use type of the RMQS subsample. Figure S5. Boxplots of the inverse Simpson indexes for each land use type of the RMQS subsample. [file 12859_2020_3829_MOESM8_ESM.pdf]
